# Oxidative hydrolysis of aliphatic bromoalkenes: scope study and reactivity insights

**DOI:** 10.3762/bjoc.20.111

**Published:** 2024-06-03

**Authors:** Amol P Jadhav, Claude Y Legault

**Affiliations:** 1 Department of Chemistry, Centre in Green Chemistry and Catalysis, Université de Sherbrooke, Québec J1K 2R1, Canada

**Keywords:** bromoalkenes, bromoketones, hypervalent iodine, oxidative hydrolysis, Ritter-type

## Abstract

We have developed an operationally simple method for the synthesis of dialkyl α-bromoketones from bromoalkenes by utilizing a hypervalent iodine-catalyzed oxidative hydrolysis reaction. This catalytic process provides both symmetrical and unsymmetrical dialkyl bromoketones with moderate yields across a broad range of bromoalkene substrates. Our studies also reveal the formation of Ritter-type side products by an alternative reaction pathway.

## Introduction

Organic synthesis heavily relies on oxidative transformations to facilitate chemical reactions. One popular method for achieving these transformations is using redox-active metals, inspired by Nature's metalloproteins. However, using toxic and expensive metals is not always practical, making alternative oxidative methodologies more appealing. Enter hypervalent iodine reagents – a leading metal-free choice for oxidation reactions. These robust and low-toxicity reagents have gained popularity due to their commercial availability [1–5] and versatility for phenolic dearomatizations, oxidative annulations, fragmentations, and oxidative rearrangements [6–11]. In particular, iodine(III) reagents have been proven effective for a wide range of oxidative transformations, cementing their position as a go-to option for organic chemists.

**Scheme 1:**
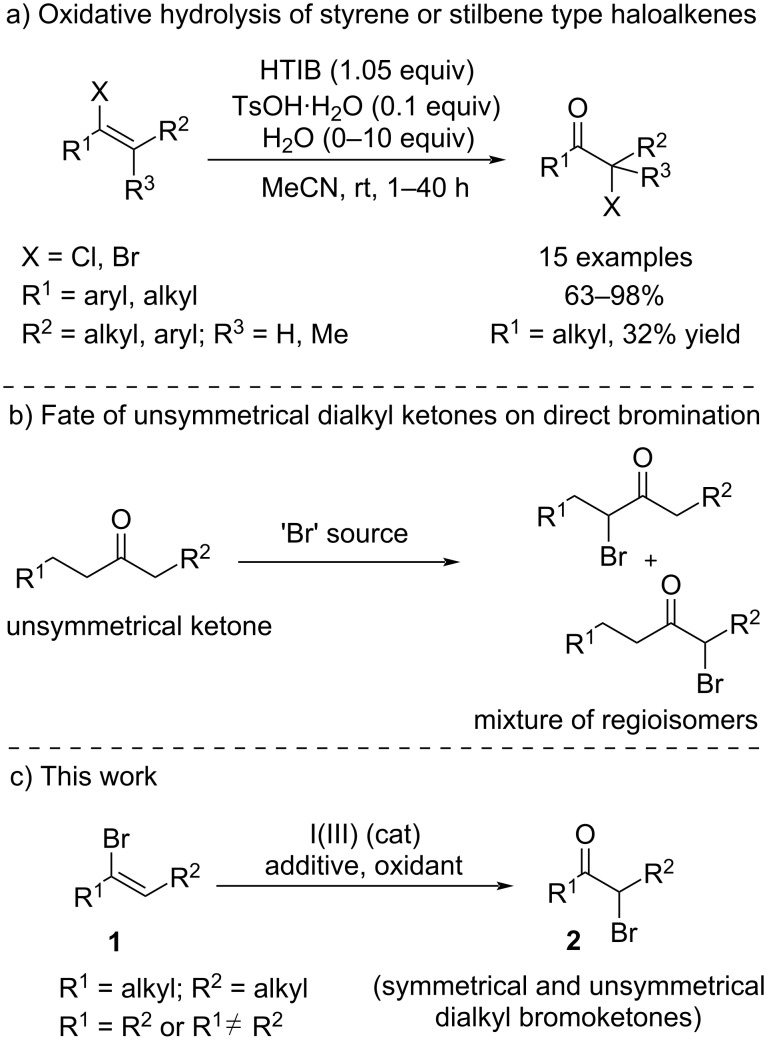
(a) Oxidative hydrolysis of styrene or stilbene type haloalkenes. (b) Fate of unsymmetrical dialkyl ketones on direct bromination. (c) This work.

Based on our continued interest in iodine(III)-mediated chemistry, we have explored numerous strategies in oxidative transformations such as direct α-tosyloxylation of ketones [12–14], and the oxidation of enol esters [15–16], to access α-functionalized ketones. We recently developed the oxidative contraction of 3,4-dihydropyranones to access polysubstituted γ-butyrolactones [17]. In 2015 we demonstrated that [hydroxy(tosyloxy)iodo]benzene (HTIB) could be used to convert chloro- and bromoalkenes into their corresponding α-halo ketone products in usually very high yields (Scheme 1a) [18–19]. However, the haloalkenes used in this previous study as α-substituted ketone precursors were limited to either styryl analogs or stilbene type haloalkenes, with the only exception of 1-bromocycloheptene as fully aliphatic substituted substrate which resulted in a low yield of the desired product. Also, this method involved using a stoichiometric amount of HTIB for the transformation.

α-Haloketones are 1,2-difunctionalized synthons which are very versatile and essential building blocks for their role in the synthesis of heterocyclic compounds [20–22]. Particularly dialkyl bromoketones have been utilized in natural product synthesis [23–25], also as a precursor to reactive oxyallyl cation intermediates [26–28], and for their photochemical reactions [29]. However, the direct halogenation of unsymmetrical ketones for the synthesis of dialkyl bromoketones would result in a mixture of regioisomers given the presence of enolizable protons on each side of the ketone (Scheme 1b). Recently Toy et al. have disclosed the selective synthesis of unsymmetrical α-haloketones by reductive halogenation of an α,β-unsaturated ketones using external halide source [30]. We envisioned that dialkyl bromoalkenes **1** could be used as enol analogs with an improvement in reaction conditions in the presence of I(III) reagents to directly get both symmetrical and unsymmetrical dialkyl bromoketones **2** (Scheme 1c). Recent methods have been reported to access bromoalkenes such as **1** from easily accessible substrates, making the approach even more appealing [31–32].

## Results and Discussion

Given its low volatility, we initiated our studies by testing the reactivity of (*E*/*Z*)-1,8-diphenyl-4-bromooct-4-ene (**1a**) with HTIB (1.1 equiv) and cat. TsOH·H_2_O (0.2 equiv) in acetonitrile. These reaction conditions afforded the desired product **2a** in moderate yield (51%), along with 21% mixture of regioisomers **3a** and **3a’** obtained from Ritter-type reaction of **1a** with CH_3_CN in the presence of HTIB (Table 1, entry 1).

**Table 1:** Conditions screening (without oxidant)^a^.

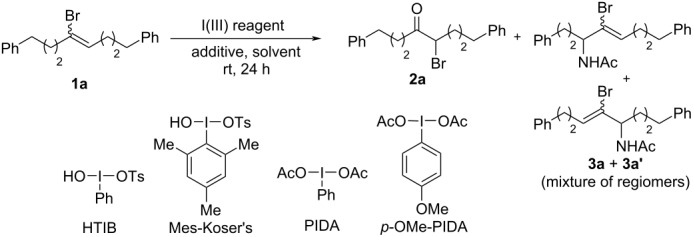					

Entry	Solvent	HVI source (equiv)	Additive (equiv)	**2a** [%]^b^	**3a** + **3a'** [%]^b,c^

1	CH_3_CN	HTIB (1.1)	TsOH·H_2_O (0.2)	51	21
2	CH_3_CN	Mes-Koser's (1.1)	TsOH·H_2_O (0.2)	36	18
3	CH_3_CN	PIDA (1.1)	TsOH·H_2_O (2.0)	42	28
4	CH_3_CN	*p*-OMe-PIDA (1.1)	TsOH·H_2_O (2.0)	50	18
5^d^	CH_3_CN	PIDA (1.1)	MsOH (2.0)	46	28
6^d^	CH_3_CN	PIDA (1.1)	HNTf_2_ (2.0)	35	trace
7	CH_2_Cl_2_	HTIB (1.1)	TsOH·H_2_O (0.2)	25	na
8	EtOAc	HTIB (1.1)	TsOH·H_2_O (0.2)	NR	na
9	DMA	HTIB (1.1)	TsOH·H_2_O (0.2)	NR	na
10	HFIP	HTIB (1.1)	TsOH·H_2_O (1.1)	0	na

^a^Unless otherwise stated 0.1 mmol of **1a** was used with 0.1 M conc. of solvent. ^b^NMR yield determined by ^1^H NMR of the crude reaction mixture using an internal standard. ^c^Combined yield of regioisomers. ^d^5.0 equiv H_2_O were added to the reaction.

We explored the influence of different variables to counteract the formation of **3a** and **3a’**. We first envisioned that the use of the more hindered, mesityl-derived Koser’s reagent, could drastically influence the formation of the side-products. Unfortunately, its use resulted in a drop of the yield for the desired α-bromoketone (Table 1, entry 2). In situ generation of Koser-like reagent by addition of excess TsOH·H_2_O (2.0 equiv) to either PIDA or *p*-OMe-PIDA did not further improve the yield for α-bromoketone (Table 1, entries 3 and 4). We envisioned that altering the iodonium intermediate counterion by replacing TsOH with either MsOH or HNTf_2_ as an acid additive (2.0 equiv) could influence the formation of **3a**/**3a’**. The use of these acids in the presence of PIDA did not show any significantly altering reaction outcome (Table 1, entries 5 and 6). We then replaced acetonitrile with dichloromethane to completely prevent the formation of **3a**/**3a’**. Unfortunately, while it eliminated the side products, it further limited the yield for α-bromoketone, whereas no reactivity was seen when EtOAc and DMA were used as solvents (Table 1, entries 7–9). The use of HFIP led to complete conversion of **1a**, but no observation of the desired product **2a** (Table 1, entry 10).

We then explored catalytic conditions for the generation of the iodine(III) reagent. Remarkably, when catalytic PhI (0.2 equiv) was employed for in situ generation of Koser’s reagent by using *m*-CPBA (1.2 equiv) as an oxidant, almost similar results were obtained (Table 2, entry 1) with those obtained by stoichiometric use of HTIB. Attempt to perform the reaction using a catalytic amount of 2-iodobenzoic acid (0.2) under similar oxidizing conditions resulted in slightly diminished yield for the desired *α*-bromoketone (Table 2, entry 2). Notably, the direct use of HTIB as the catalyst, with a catalytic amount of TsOH·H_2_O (0.2 equiv each), in the presence of *m*-CPBA (1.2 equiv) proved to be the most superior conditions (59% NMR yield, Table 2, entry 3). To rule out the possibility of direct involvement of *m*-CPBA in the oxidative hydrolysis reaction, **1a** was reacted in the absence of any hypervalent iodine source, which resulted in a significant decrease in the yield of **2a** (Table 2, entries 4 and 5). Importantly, when analogous chloroalkene (*E*/*Z*)-1,8-diphenyl-4-chlorooct-4-ene (**1a’**) was tested as a substrate under optimal conditions (without H_2_O or with 5 equiv H_2_O), no reactivity was seen at all, presumably due to the stronger inductive effect of the chlorine (Table 2, entry 6).

**Table 2:** Conditions screening (with oxidant)^a^.

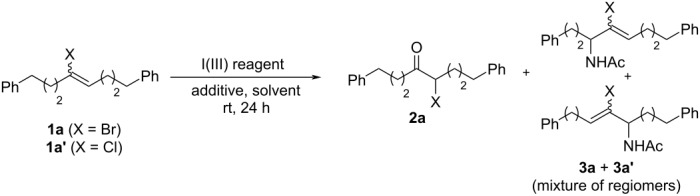						

Entry	Solvent	Catalyst (equiv)	Additive (equiv)	Oxidant (equiv)	**2a** [%]^b^	**3a** + **3a'** [%]^b,c^

1	CH_3_CN	PhI (0.2)	TsOH·H_2_O (0.2)	*m-*CPBA (1.2)	51	23
2	CH_3_CN	2-I-PhCO_2_H (0.2)	TsOH·H_2_O (0.2)	*m-*CPBA (1.2)	41	22
3	CH_3_CN	HTIB (0.2)	TsOH·H_2_O (0.2)	*m-*CPBA (1.2)	59	24
4	CH_3_CN	none	none	*m-*CPBA (1.2)	5	20
5	CH_3_CN	none	TsOH·H_2_O (1.1)	*m-*CPBA (1.2)	13	15
6^d^	CH_3_CN	HTIB (0.2)	TsOH·H_2_O (0.2)	*m-*CPBA (1.2)	na	na

^a^Unless otherwise stated 0.1 mmol of **1a** was used with 0.1 M conc. of solvent. ^b^NMR yield determined by ^1^H NMR of the crude reaction mixture using an internal standard. ^c^Combined yield of regioisomers. ^d^X = Cl and the reaction was carried out both with or without addition of 5.0 equiv of H_2_O.

**Scheme 2:**
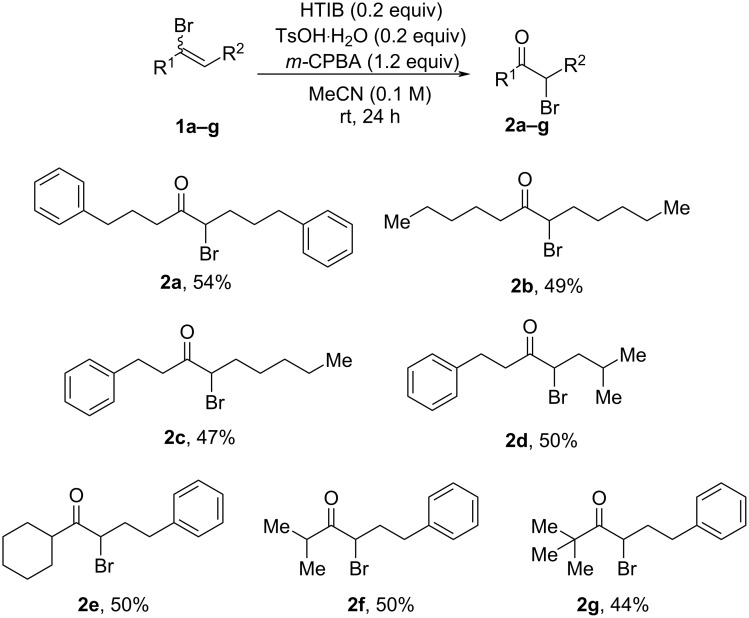
Substrate scope. Unless otherwise stated 0.2 mmol of **1** was used and the isolated yields are given.

**Scheme 3:**
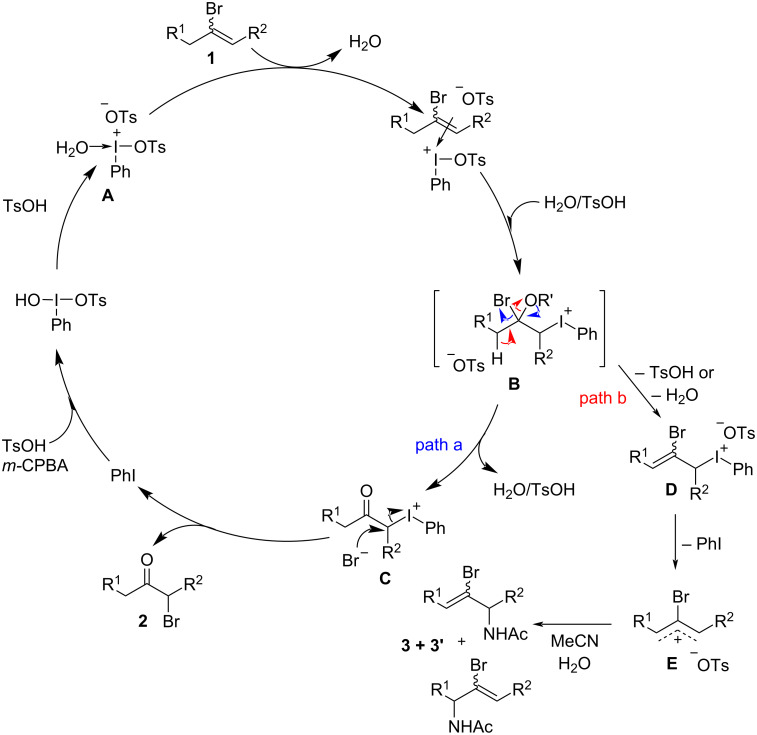
Proposed catalytic cycle.

It was unfortunately not possible to prevent formation of side products **3a**/**3a'** using modifications of the reaction conditions. We thus next turned our attention to exploring the scope of the developed protocols, focusing both on symmetrical as well as unsymmetrical dialkyl bromoalkenes, in order to determine if the nature of the substrate could influence the reaction outcome. As shown in Scheme 2, the (*E*/*Z*) symmetrical dialkyl bromoalkenes reacted well with catalytic HTIB, irrespective of the chain length, affording the corresponding α-bromoketones (**2a**,**b**) in 49–54% isolated yields by oxidative transposition of the bromine atom in the reaction process. We then extended this scope by synthesizing unsymmetrical dialkyl bromoalkenes (**1c**–**g**) bearing side chains of varied length and steric character. The incorporation of *n*-pentyl or isobutyl groups at the distal side of bromoalkene was readily tolerated and yielded the products (**2c**,**d**) with consistent yields. Demonstrating additional generalizability, substrates bearing sterically demanding cyclohexyl or isopropyl groups as the near side chain of bromoalkene afforded the corresponding α-bromoketones (**2e**,**f**) with unaffected reactivity or yields. Notably, 15–20% Ritter-type side products were obtained with all these substrates as a mixture of regioisomers. Surprisingly, even substrate **2g** did not provide a higher yield of the desired α-bromoketone product, despite the absence of hydrogens on the allylic position (see Scheme 3 for explanations).

Our mechanistic understanding of the oxidative hydrolysis of styrene haloalkene analogs [19] lets us hypothesize an external bromide attack as the main reaction pathway for this catalytic oxidative transposition of dialkyl bromoalkenes (Scheme 3). No α-tosyloxy ketone products were observed in the crude reaction mixtures, either with catalytic or stoichiometric use of TsOH·H_2_O, even when the reactions were incomplete. These observations ruled out the possibility of double S_N_2 attack by tosylate followed by bromide.

TsOH·H_2_O accelerates the formation of the phenyl tosyloxy iodonium intermediate **A** from catalytic HTIB. Dialkyl bromoalkene **1** then associates with **A** followed by attack of tosyloxy or water, delivering iodonium intermediate **B**. Being a better leaving group, the bromide anion is then expelled, which becomes a counterion for the iodonium intermediate **C**. Liberation of PhI serves as the driving force for subsequent S_N_2 attack by the bromide anion to give the dialkyl α-bromoketone **2**. *m-*CPBA then regenerates the hypervalent iodine (HTIB) catalyst by oxidizing PhI in the presence of TsOH·H_2_O. The formation of the Ritter-type side products is proposed through path b (Scheme 3). The elimination of α-proton on the side chain of dialkyl bromoalkenes results in iodonium intermediate **D**, which on the expulsion of PhI gives a mixture of the allylic carbocation **E**, which ultimately gets trapped by MeCN in the presence of H_2_O, giving the regioisomeric mixture of Ritter-type amidation side products **3**.

## Conclusion

In summary we have developed a hypervalent iodine-catalyzed synthetic method for the oxidative hydrolysis of diverse dialkyl bromoalkenes. The current approach can tolerate both symmetrical as well as unsymmetrical dialkyl bromoalkenes as substrates delivering dialkyl α-bromoketones which are highly sought-after synthons in heterocycle synthesis and medicinal chemistry, thus overcoming the limitations of previous methods. The reaction accommodates sterically hindered bromoalkenes as substrates, leading to the corresponding α-bromoketone derivatives. While we could not further minimize the formation of Ritter-type side products (≈4:1 ratio of α-bromoketone vs Ritter-type side products), noticing these side products from common phenyl tosyloxy iodonium intermediate suggest that hypervalent iodine reagents could be utilized in the future for the α-acetamidation of dialkyl bromoalkenes. The present work provides an operationally simple catalytic method to access a diverse range of α-bromoketones, which are versatile building blocks for synthesizing various important hetero aromatics.

## Supporting Information

File 1 Experimental procedures for reactions, and relevant spectra of all new compounds.

## Data Availability

All data that supports the findings of this study is available in the published article and/or the supporting information to this article.
