# Supplementary material for: Oxidative hydrolysis of aliphatic bromoalkenes: scope study and reactivity insights
Source: Beilstein J Org Chem. 2024 Jun 3;20:1286–91. doi: 10.3762/bjoc.20.111 (PMC11181184; doi:10.3762/bjoc.20.111)
Supplement: File 1 — Experimental procedures for reactions, and relevant spectra of all new compounds. [file Beilstein_J_Org_Chem-20-1286-s001.pdf]

BEILSTEIN JOURNAL OF ORGANIC CHEMISTRY

Supporting Information

for

Oxidative hydrolysis of aliphatic bromoalkenes: scope study and reactivity insights

Amol P. Jadhav and Claude Y. Legault

Beilstein J. Org. Chem. **2024,** *20,* 1286–1291. doi:10.3762/bjoc.20.111

Experimental procedures for reactions, and relevant spectra of all new compounds

License and Terms: This is a supporting information file under the terms of the Creative Commons Attribution License (https://creativecommons.org/licenses/by/4.0). Please note that the reuse, redistribution and reproduction in particular requires that the author(s) and source are credited and that individual graphics may be subject to special legal provisions.
The license is subject to the *Beilstein Journal of Organic Chemistry* terms and conditions: (https://www.beilstein-journals.org/bjoc/terms)

Table of Contents

General details S2

Preparation of substrates S3

Oxidative hydrolysis of bromoalkenes S11

NMR Spectra S15

General Details

All non-aqueous reactions involving air or moisture-sensitive compounds were run under an inert atmosphere (nitrogen or argon) with rigid exclusion of moisture from reagents and glassware using standard techniques.[1] All glassware was stored in the oven and/or was flame-dried prior to use under an inert atmosphere of gas. Anhydrous solvents were obtained either by distillation over sodium (THF, ether), or over calcium hydride (CH_2Cl_2). Analytical thin-layer chromatography (TLC) was performed on precoated, glass-backed silica gel (Merck 60 F254). Visualization of the developed chromatogram was performed by UV absorbance, aqueous cerium molybdate, ethanolic phosphomolybdic acid, iodine, or aqueous potassium permanganate. Flash column chromatography was performed using 230–400 mesh silica (EM Science or Silicycle) of the indicated solvent system according to standard technique.[2] Infrared spectra were taken on a Perkin Elmer Spectrum One FTIR and are reported in reciprocal centimeters (cm^{-1}). Nuclear magnetic resonance spectra (^1H, ^{13}C, DEPT, COSY, HMQC) were recorded either on a Bruker Avance III HD 300 or Varian Mercury+ 400 spectrometer. Chemical shifts for ^1H NMR spectra are recorded in parts per million from tetramethylsilane with the solvent resonance as the internal standard. Data are reported as follows: chemical shift, multiplicity (s = singlet, d = doublet, t = triplet, q = quartet, qn = quintet, sext = sextuplet, m = multiplet and br = broad), coupling constant in Hz, integration. Chemical shifts for ^{13}C NMR spectra are recorded in parts per million from tetramethylsilane with the solvent resonance as the internal standard. High resolution mass spectra were performed at Université de Sherbrooke. Analytical High Performance Liquid Chromatography was performed on Shimadzu Prominence LC system equipped with diode array UV detector. Data are reported as follows: (column type, eluent, flow rate: retention time (*tr*)).

Note 1: Our attempts to obtain HRMS in Q-TOF for the vinyl halides **1b**–**g** *were not successful.*

GC–MS (EI) were obtained instead for these compounds. The fragmentation patterns are consistent with the molecular formula. Furthermore, a report by Prati et al. has shown that for similar vinyl halides, the GC–MS are always consistent with elemental analysis.

---

1. Shriver, D. F.; Drezdzon, M. A. *The manipulation of air-sensitive compounds*; 2nd Edition; Wiley: New York, **1986**.
2. Still, W.C.; Kahn, M.; Mitra A. *J. Org. Chem.* **1978**, *43*, 2923.
3. Spaggiari, A; Vaccari, D.; Davoli, P.; Torre, G.; Prati, F. *J. Org. Chem.* **2007**, *72*, 2216.

Preparation of substrates

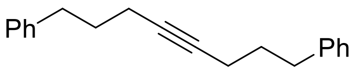

A1

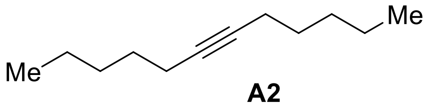

A2

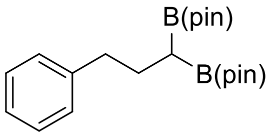

B1

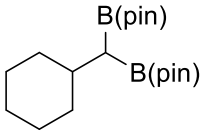

B2

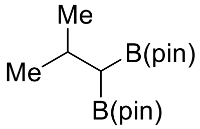

B3

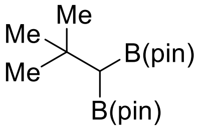

B4

The symmetrical alkynes **A1** were prepared according to the known literature procedure.[4] 6-Dodecyne (**A2**) was purchased from TCI America and used as received. Geminal bis(boronate) esters **B1** and **B4** were prepared according to the known literature procedure from the corresponding germinal dibromides.[5] Geminal bis(boronate) esters **B2** and **B4** were prepared according to the known literature procedure directly from the corresponding aldehydes.[6]

General procedure for the preparation of symmetrical bromoalkenes (1a–b)[7]

Method A:

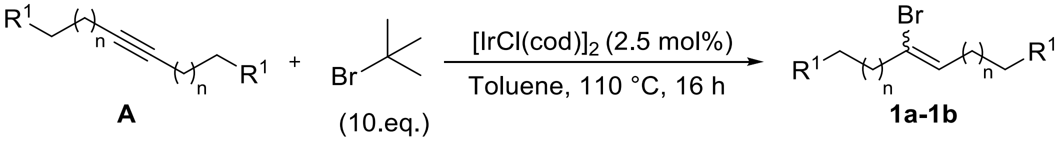

In the glove box, to a 15 mL pressure tube equipped with a magnetic stirring bar was added $[IrCl(cod)]_2$ (2.5 mol %). The pressure tube was removed from the glove box and anhydrous toluene (0.5 M) was added under argon atmosphere. Alkyne **A** (1.0 equiv) and *tert*-butyl bromide (10.0 equiv.) were then added sequentially to the resulting solution, and the pressure tube was sealed and heated at 110 °C in an oil bath. After stirring for 16 hours, the reaction mixture was cooled to room temperature. The resulting solution was concentrated under reduced pressure and purified using silica gel column chromatography (100% pentane or 10% CH_2Cl_2 in hexane) to give the symmetrical bromoalkenes **1a** and **1b**. The ratio of (*Z*/*E*) isomers was assigned based on either data reported in literature or analogy to known similar compounds.

4. Lee, Y. H.; Denton, E. H.; Morandi, B. *J. Am. Chem. Soc.* **2020**, *142*, 20948−20955
5. Coombs, J. R.; Zhang, L.; Morken, J. P. *Org. Lett.* **2015**, *17*, 1708−1711
6. Kumar, N.; Reddy, R. R.; Masarwa, A. *Chem. Eur. J.* **2019**, *25*, 8008–8012

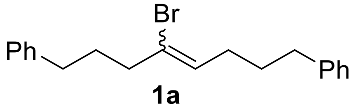

1a

(4-Bromooct-4-ene-1,8-diyl)dibenzene (**1a**): Prepared according to the general procedure (Method **A**) utilizing $[IrCl(cod)]_2$ (44.8 mg, 0.067 mmol), 1,8-diphenyloct-4-yne **A1** (700 mg, 2.67 mmol) and *tert*-butyl bromide (3.0 mL, 26.7 mmol) in anhydrous toluene (5.3 mL). The crude reaction mixture was purified by column chromatography on silica gel (10% CH_2Cl_2 in hexanes, R_f = 0.48) to afford the desired product **1a** as a colorless liquid (650 mg, 71% yield, *Z*/*E* = 60/40), the (*Z*/*E*) ratio for compound **1a** was assigned based on analogy to compound **1b**. ^{1}H NMR (300 MHz, $CDCl_3$) δ 7.36-7.31 (m, 4H), 7.26-7.19 (m, 6H), 5.95 (t, J = 7.6 Hz, 1H =CH_E), 5.70 (t, J = 6.8 Hz, 1H =CH_Z), 2.71-2.44 (m, 4H), 2.53-2.44 (m, 2H), 2.26 (dd, 14.6, 7.2 Hz, 1H), 2.08-1.90 (m, 3H), 1.77 (td, J = 15.0, 7.7 Hz, 2H); ^{13}C NMR (75 MHz, $CDCl_3$) δ 142.3, 142.0, 141.9, 141.8, 132.5, 128.7, 128.6, 128.5, 128.4, 128.3, 128.2, 41.6, 35.6, 35.3, 35.0, 34.7, 34.6, 31.1, 30.9, 30.4, 29.9, 29.8, 29.2; IR (neat) 2927, 2858, 1496, 1453, 1030, 741, 695 cm^{-1}; HRMS ESI-Q-TOF calcd for $C_{20}H_{23}BrNa$ $[MNa]^+$ 365.0875, found 365.0885

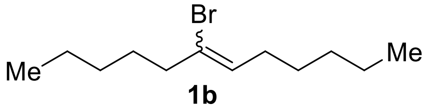

1b

6-Bromododec-6-ene (**1b**): Prepared according to the general procedure (Method **A**) utilizing $[IrCl(cod)]_2$ (16.8 mg, 0.025 mmol), dodec-6-yne **A2** (166 mg, 1.0 mmol) and *tert*-butyl bromide (1.12 mL, 10.0 mmol) in anhydrous toluene (2.0 mL). The crude reaction mixture was purified by column chromatography on silica gel (*n*-Pentane, R_f = 0.8) to afford the desired product **1b** as a colorless liquid (210 mg, 85% yield, *Z*/*E* = 69/31), the (*Z*/*E*) ratio for compound **1b** was determined based on the data reported in literature[7]. ^{1}H NMR (300 MHz, $CDCl_3$) δ 5.85 (t, J = 7.6 Hz, 1H =CH_E), 5.61 (t, J = 6.8 Hz, 1H =CH_Z), 2.43-2.36 (m, 2H), 2.14 (dd, J = 14.0, 7.0 Hz, 1H =CH_Z), 2.02 (dd, J = 14.6, 7.3 Hz, 1H =CH_E), 1.60-1.50 (m, 2H), 1.44-1.20 (m, 10H); ^{13}C NMR (75 MHz, $CDCl_3$) δ 132.6, 128.6, 128.5, 126.1, 41.7, 35.5, 31.6, 31.5, 31.4, 30.9, 30.7, 29.7, 29.1, 28.4, 28.0, 27.9, 22.7, 22.6, 22.5 14.2, 14.1; IR (neat) 2957, 2858, 1459, 1379, 1138, 728.7 cm^{-1}; LRMS-EI (m/z) calcd for $C_{12}H_{23}Br$ $[M]^{+\cdot}$ 246.00983, found 246.0

7. Yu, P.; Bismuto, A.; Morandi, B. *Angew. Chem. Int. Ed.* **2020**, *59*, 2904–2910
8. Ganic´ A.; Pfaltz, A. *Chem. Eur. J.* **2012**, *18*, 6724–6728
9. Roman, U. V.; Ruhdorfer, J.; Knorr, R. *Synthesis*, **1993**, *10*, 985–992

General procedure for the preparation of unsymmetrical bromoalkenes (1c–g)[5]

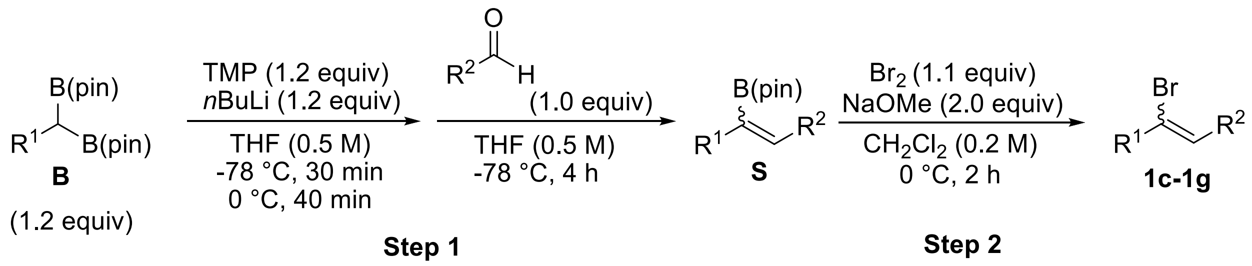

Method B

To a solution of 2,2,6,6-tetramethylpiperidine (TMP) (1.3 equiv) in anhydrous THF (0.5 M) at −78 °C was slowly added *n*-BuLi (2.5 M, 1.3 equiv) under argon atmosphere. The solution was allowed to stir for 30 minutes at this temperature, followed by stirring for additional 30 minutes at 0 °C. A solution of 1,1-diboronate **B** (1.2 equiv) in anhydrous THF (0.25 M) was slowly added to this reaction mixture at 0 °C. It was allowed to stir for 10 minutes at this temperature before cooling it down at −78 °C. A solution of aldehyde (1.0 equiv) in anhydrous THF (0.5 M) was then slowly added to the reaction flask followed by additional stirring up to 4 h at −78 °C. Upon complete consumption of aldehyde, the reaction mixture was directly concentrated under reduced pressure. The crude reaction mixture was purified by column chromatography on silica gel (4% Et_2O in hexanes) to afford the desired trisubstituted-vinyl boronate product **S** as a clear, colorless oil as a mixture of *Z*/*E* isomers.

Method C

Following a modified procedure[5] trisubstituted-vinyl boronate compound **S** (1.0 equiv) was weighed into a 25-mL round bottom flask equipped with a magnetic stir bar. It was then dissolved in anhydrous CH_2Cl_2 (0.2 M) and the reaction flask was placed in an ice-water bath at 0 °C. Bromine (1.1 equiv) was added drop-wise via syringe to this solution. The reaction was allowed to stir at 0 °C for 1 h, after which time a solution of sodium methoxide (0.6 M in anhydrous MeOH, 2.0 equiv) was added via a syringe. The solution was allowed to stir at 0 °C for 1 h, after which time a saturated solution of aqueous sodium thiosulfate (5 mL) was added. The aqueous layer was extracted with CH_2Cl_2 (3 × 5 mL). The organic layer was dried over magnesium sulfate, filtered, and concentrated in vacuo. The crude residue was then purified using column chromatography on silica gel (2% Et_2O in hexanes) to afford the desired unsymmetrical bromoalkene **1c**–**g** as a mixture of *Z*/*E* isomers.

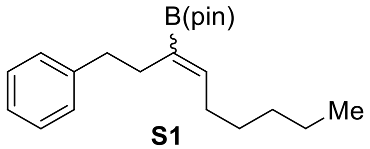

S1

4,4,5,5-Tetramethyl-2-(1-phenylnon-3-en-3-yl)-1,3,2-dioxaboro-lane (**S1)**: The title compound was obtained from 1,1-diboronate **B1** (447 mg, 1.2 mmol), 1-hexanal (100 mg, 1.0 mmol), TMP (0.219 mL, 1.3 mmol) and n-BuLi (0.58 mL, 1.3 mmol, 2.25 M in hexanes) in anhydrous THF (4.0 mL) according to the general procedure (Method **B**). The crude reaction mixture was purified by column chromatography on silica gel (4% Et_2O in hexanes, R_f = 0.55) to afford the desired product **S1** as a colorless liquid (307 mg, 94% yield, *Z*/*E* = 91/09). The ratio of geometric isomers (*Z*/*E*) for compound **S1** was determined based on the data reported in literature[5]. ^{1}H NMR (300 MHz, $CDCl_3$) δ (major isomer) 7.35-7.25 (m, 2H), 7.21-7.14 (m, 3H), 6.32 (t, *J* = 7.1 Hz, 1H), 2.66 (t, *J* = 7.8 Hz, 2H), 2.45 (t, *J* = 7.8 Hz), 2.02 (q, *J* = 7.2 Hz), 1.33-1.26 (m, 18H), 0.91-0.86 (m, 3H); ^{13}C NMR (75 MHz, $CDCl_3$) δ (major isomer); 147.0, 142.8, 128.7, 128.2, 125.6, 88.1, 36.5, 31.9, 30.8, 28.9, 28.6, 24.9, 22.7, 14.2; ^{11}B NMR (96 MHz, CD_3Cl): δ (major isomer) 30.5 (s)(br); IR (neat) 2927, 2858, 1379, 1308, 1144, 697 cm^{-1}; HRMS ESI-Q-TOF calcd for $C_{21}H_{33}BO_2Na$ $[MNa]^+$ 351.2470, found 351.2470

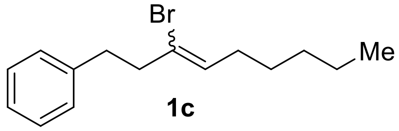

1c

(3-Bromonon-3-enyl)benzene (**1c)**: The title compound was obtained from **S1** (136 mg, 0.41 mmol), bromine (23.4 µL, 0.45 mmol) and NaOMe (1.52 mL, 0.6 M in MeOH) in anhydrous CH_2Cl_2 (2.1 mL, 0.2 M) according to the general procedure (Method **C**). The crude reaction mixture was purified by column chromatography on silica gel (2% Et_2O in hexanes, R_f = 0.65) to afford the desired product **1c** as a colorless liquid (117 mg, 66% yield, *Z*/*E* = 89/11). The ratio of geometric isomers (*Z*/*E*) for compound **1c** was assigned based on analogy to compound **1b**. ^{1}H NMR (300 MHz, $CDCl_3$) δ (major isomer) 7.31-7.26 (m, 2H), 7.22-7.17 (m, 3H), 5.54 (t, *J* = 6.9 Hz, 1H), 2.87 (dd, *J* = 8.5, 6.3 Hz, 2H), 2.70 (t, *J* = 6.5 Hz, 2H), 2.11 (q, *J* = 7.0 Hz, 2H), 1.38-1.15 (m, 6H), 0.91-0.84 (m, 3H); ^{13}C NMR (75 MHz, $CDCl_3$) δ (major isomer) 140.9, 129.8, 128.8, 128.7, 128.5, 128.4, 127.1, 126.2, 43.7, 34.8, 31.4, 31.3, 28.2, 22.6, 14.2; IR (neat) 2927, 2858, 1459, 1379, 1138, 728 cm^{-1}; LRMS-EI (m/z) calcd for $C_{15}H_{21}Br$ $[M]^{+\cdot}$ 280.0827, $C_{12}H_{23}Br$ $[M\text{-}Br]^{+\cdot}$ 201.1638, found 282.0, 201.1

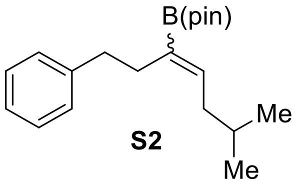

S2

4,4,5,5-Tetramethyl-2-(6-methyl-1-phenylhept-3-en-3-yl)-1,3,2-dioxaborolane (**S2)**: The title compound was obtained from 1,1-diboronate **B1** (447 mg, 1.2 mmol), isovaleraldehyde (86.1 mg, 1.0 mmol), TMP (0.219 mL, 1.3 mmol) and *n*-BuLi (0.58 mL, 1.3 mmol, 2.25 M in hexanes) in anhydrous THF (4.0 mL) according to the general procedure (Method **B**). The crude reaction mixture was purified by column chromatography on silica gel (4% Et_2O in hexanes, R_f = 0.49) to afford the desired product **S2** as a colorless liquid (295 mg, 94% yield, *Z*/*E* = 77/23). The ratio of geometric isomers (*Z*/*E*) for compound **S2** was determined based on the data reported in literature[5]. ^{1}H NMR (300 MHz, $CDCl_3$) δ (major isomer) 7.37-7.26 (m, 2H), 7.25-7.15 (m, 3H), 6.36 (t, J = 7.2 Hz, 1H), 2.73-2.40 (m, 2H), 2.46 (t, J = 8.8 Hz, 2H), 1.96 (t, J = 7.0 Hz, 2H), 1.69-1.60 (m, 1H), 1.27 (s, 12H), 0.90 (d, 6.2 Hz, 6H); ^{13}C NMR (75 MHz, $CDCl_3$) δ (major isomer); 145.7, 142.9, 128.8, 128.2, 125.6, 83.1, 37.7, 36.4, 30.8, 28.4, 24.9, 22.8; ^{11}B NMR (96 MHz, CD_3Cl): δ (major isomer) 30.8 (s)(br); IR (neat) 2955, 1628, 1379, 1302, 1142, 857, 697 cm^{-1}; HRMS ESI-Q-TOF calcd for $C_{20}H_{31}BO_2Na$ $[MNa]^+$ 337.2313, found 337.2317

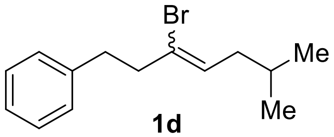

1d

(3-Bromo-6-methylhept-3-enyl)benzene (**1d)**: The title compound was obtained from **S2** (295 mg, 0.94 mmol), bromine (53.1 μL, 1.03 mmol) and NaOMe (3.13 mL, 0.6 M in MeOH) in anhydrous CH_2Cl_2 (4.7 mL, 0.2 M) according to the general procedure (Method **C**). The crude reaction mixture was purified by column chromatography on silica gel (2% Et_2O in hexanes, R_f = 0.58) to afford the desired product **1d** as a colorless liquid. (210 mg, 84% yield, *Z*/*E* = 71/29). The ratio of geometric isomers (*Z*/*E*) for compound **1d** was assigned based on analogy to compound **1b**. ^{1}H NMR (300 MHz, $CDCl_3$) δ (major isomer) 7.31-7.25 (m, 2H), 7.24-7.17 (m, 3H), 5.54 (t, J = 7.0 Hz, 1H), 2.86 (q, J = 6.2 Hz, 2H), 2.71 (t, J = 6.8 Hz, 2H), 2.01 (t, J = 6.9 Hz, 2H), 1.73-1.58 (m, 1H), 0.83 (d, J = 6.1 Hz, 2H); ^{13}C NMR (75 MHz, $CDCl_3$) δ (major isomer) 140.8, 132,5, 128.7, 128.6, 128.4, 126.2, 43.8, 40.4, 34.8, 28.2, 22.4; IR (neat) 2955, 1455, 1168, 1028, 747, 697 cm^{-1}; LRMS-EI (m/z) calcd for $C_{14}H_{19}Br$ $[M]^{+\cdot}$ 266.0670, $C_{14}H_{19}$ $[M\text{-}Br]^{+\cdot}$ 187.1481, found 266.0, 187.1

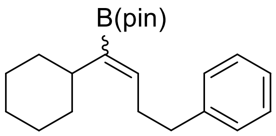

S3

2-(1-Cyclohexyl-4-phenylbut-1-enyl)-4,4,5,5-tetramethyl-1,3,2-dioxaborolane (**S3**): The title compound was obtained from 1,1-diboronate **B2** (420 mg, 1.2 mmol), hydrocinnamaldehyde (0.132 mL, 1.0 mmol), TMP (0.219 mL, 1.3 mmol) and *n*-BuLi (0.58 mL, 1.3 mmol, 2.25 M in hexanes) in anhydrous THF (4.0 mL) according to the general procedure (Method **B**). The crude reaction mixture was purified by column chromatography on silica gel (4% Et_2O in hexanes, R_f = 0.42) to afford the desired product **S2** as a colorless liquid (310 mg, 91% yield, *Z*/*E* = 56/44). The ratio of geometric isomers (*Z*/*E*) for compound **S3** was determined based on the data reported in literature[8]. ^{1}H NMR (300 MHz, $CDCl_3$) δ 7.30-7.25 (m, 2H), 7.24-7.11 (m, 3H), 6.23 (t, *J* = 6.9 Hz, 1H =CH_Z), 5.97 (t, *J* = 7.3 Hz, 1H =CH_E), 2.73-2.57 (m, 2H), 2.56-2.29 (m, 2H), 1.70-1.47 (m, 6H), 1.38-1.32 (m, 2H), 1.27 (s, 12H), 1.22-1.05 (m, 3H); ^{13}C NMR (75 MHz, $CDCl_3$) δ 142.5, 142.4, 140.4, 128.7, 128.5, 128.4, 128.3, 125.9, 125.7, 83.0, 82.9, 82.6, 44.5, 39.5, 36.9, 36.1, 35.9, 34.3, 33.6, 33.3, 32.0, 30.7, 27.0, 26.9, 26.7, 26.5, 26.2, 25.0, 24.9; ^{11}B NMR (96 MHz, CD_3Cl): δ 30.7 (s) (br); IR (neat) 2924, 2853, 1615, 1371, 1291, 1144, 699 cm^{-1}; HRMS ESI-Q-TOF calcd for $C_{22}H_{33}BO_2Na$ $[MNa]^+$ 363.2470, found 363.2475

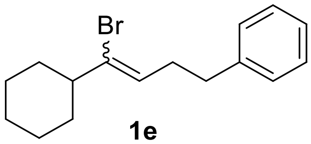

1e

(4-Bromo-4-cyclohexylbut-3-enyl)benzene (**1e**): The title compound was obtained from **S3** (300 mg, 0.88 mmol), bromine (49.7 µL, 0.97 mmol) and NaOMe (3.08 mL, 0.6 M in MeOH) in anhydrous CH_2Cl_2 (4.4 mL, 0.2 M) according to the general procedure (Method **C**). The crude reaction mixture was purified by column chromatography on silica gel (2% Et_2O in hexanes, R_f = 0.65) to afford the desired product **1e** as a colorless liquid (60 mg, 23% yield, *Z*/*E* = 66/34). The ratio of geometric isomers (*Z*/*E*) for compound **1e** was assigned based on analogy to compound **1b**. ^{1}H NMR (300 MHz, $CDCl_3$) δ 7.33-7.26 (m, 2H), 7.23-7.17 (m, 3H), 5.84 (t, *J* = 7.7 Hz, 1H =CH_E), 5.68 (t, *J* = 6.7 Hz, 1H =CH_Z), 2.70 (q, *J* = 7.0 Hz, 2H), 2.53-2.36 (m, 2H), 2.34-2.13 (m, 1H), 1.85-1.66 (m, 4 H), 1.53-1.08 (m, 6H); ^{13}C NMR (75 MHz, $CDCl_3$) δ 141.7, 141.3, 136.0, 134.4, 129.7, 128.6, 128.5, 128.4, 126.2, 126.0, 125.3, 49.0, 41.9, 35.7, 34.9, 33.1, 32.6, 31.6, 31.5, 26.3, 26.1, 26.0, 25.8; IR (neat) 2927, 2853, 1638, 1449, 892, 745, 697 cm^{-1}; LRMS-EI (m/z) calcd for $C_{16}H_{21}Br$ $[M]^{+\cdot}$ 292.0827, $C_{16}H_{21}$ $[M-Br]^{+\cdot}$ 213.1638, found 213.1

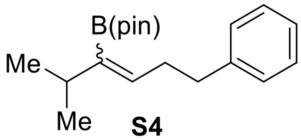

S4

4,4,5,5-Tetramethyl-2-(2-methyl-6-phenylhex-3-en-3-yl)-1,3,2-dioxaborolane (**S4**): The title compound was obtained from 1,1-diboronate **B3** (760 mg, 2.45 mmol), hydrocinnamaldehyde (0.269 mL, 2.04 mmol), TMP (0.49 mL, 2.66 mmol) and *n*-BuLi (1.06 mL, 2.66 mmol, 2.5 M in hexanes) in anhydrous THF (4.0 mL) according to the general procedure (Method **B**). The crude reaction mixture was purified by column chromatography on silica gel (10% Et_2O in hexanes, R_f = 0.63) to afford the desired product **S4** as a colorless liquid (580 mg, 95% yield, *Z*/*E* = 40/60); The ratio of geometric isomers (*Z*/*E*) for compound **S4** was determined based on the data reported in literature[5]. ^{1}H NMR (300 MHz, $CDCl_3$) δ 7.31-7.25 (m, 2H), 7.21-7.15 (m, 3H), 6.24 (t, *J* = 7.0 Hz, 1H =CH_Z), 6.01 (t, *J* = 7.1 Hz, 1H =CH_E), 2.78-2.50 (m, 4H), 2.47-2.37 (m, 1H), 1.28 (s, 12H = CH_E), 1.26 (s, 12H = CH_Z), 1.03 (dd, *J* = 6.9, 1.7 Hz, 6H); ^{13}C NMR (75 MHz, $CDCl_3$) δ 142.4, 142.3, 142.0, 128.6, 128.4, 128.3, 128.2, 125.7, 125.5, 82.8, 82.7, 36.8, 35.8, 34.3, 33.3, 30.4, 28.5, 24.8, 24.7, 22.7, 22.1; ^{11}B NMR (96 MHz, CD_3Cl): δ 30.9 (s) (br); IR (neat) 2978, 1621, 1295, 1144, 978, 855, 747, 698 cm^{-1}; HRMS ESI-Q-TOF calcd for $C_{19}H_{29}BO_2Na$ $[MNa]^+$ 323.2156, found 323.2160

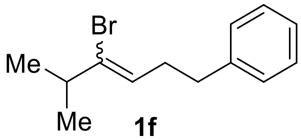

1f

(4-Bromo-5-methylhex-3-enyl)benzene (**1f**): The title compound was obtained from **S4** (450 mg, 1.5 mmol), bromine (84.5 μL, 0.97 mmol) and NaOMe (5.25 mL, 0.6 M in MeOH) in anhydrous CH_2Cl_2 (7.5 mL, 0.2 M) according to the general procedure (Method **C**). The crude reaction mixture was purified by column chromatography on silica gel (2% Et_2O in hexanes, R_f = 0.73) to afford the desired product **1f** as a colorless liquid (144 mg, 38% yield, *Z*/*E* = 50/50). The ratio of geometric isomers (*Z*/*E*) for compound **1f** was assigned based on analogy to compound **1b**. ^{1}H NMR (300 MHz, $CDCl_3$) δ 7.35-7.28 (m, 2H), 7.25-7.20 (m, 3H), 5.86 (t, *J* = 7.7 Hz, 1H =CH_E), 5.74 (t, *J* = 6.6 Hz, 1H =CH_Z), 2.83-2.70 (m, 2H), 2.62-2.39 (m, 3H), 1.13 (d, *J* = 6.7 Hz, 6H =CH_E), 0.98 (d, *J* = 6.6 Hz, 6H =CH_Z); ^{13}C NMR (75 MHz, $CDCl_3$) δ 141.7, 141.3, 137.3, 136.0, 129.4, 128.6, 128.5, 128.4, 126.2, 126.0, 125.1, 39.6, 35.7, 34.8, 32.9, 31.6, 31.4, 22.1, 21.6; IR (neat) 2968, 1638, 1455, 1116, 840.5, 745.5, 697.0 cm^{-1}; LRMS-EI (m/z) calcd for $C_{13}H_{17}Br$ $[M]^{+\cdot}$ 252.0514, $C_{13}H_{17}$ $[M-Br]^{+\cdot}$ 173.1325, found 252.0, 173.1

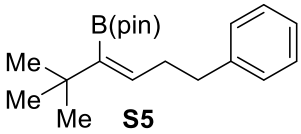

S5

(*E*)-2-(2,2-Dimethyl-6-phenylhex-3-en-3-yl)-4,4,5,5-tetramethyl-1,3,2-dioxaborolane (**S5**): The title compound was obtained from 1,1-diboronate **B4** (520 mg, 1.60 mmol), hydrocinnamaldehyde (0.185 mL, 1.34 mmol), TMP (0.29 mL, 1.74 mmol) and *n*-BuLi (0.77 mL, 1.74 mmol, 2.25 M in hexanes) in anhydrous THF (6.7 mL) according to the general procedure (Method **B**). The crude reaction mixture was purified by column chromatography on silica gel (5% Et_2O in hexanes, R_f = 0.59) to afford the desired product **S5** as a colorless liquid (240 mg, 57% yield, E-isomer only) The olefin geometry for compound **S5** was determined based on the data reported in literature[5]. ^{1}H NMR (300 MHz, $CDCl_3$) δ 7.34-7.27 (m, 2H), 7.24-7.23 (m, 1H), 7.18-7.13 (m, 2H), 5.87 (t, J = 7.5 Hz, 1H =CH_E), 2.66 (dd, J = 9.5, 6.4 Hz), 2.45-2.37 (m, 2H), 1.28 (s, 12H = CH_E), 1.06 (s, 9H); ^{13}C NMR (75 MHz, $CDCl_3$) δ 142.4, 135.1, 134.0, 133.7, 128.6, 128.3, 125.7, 83.3, 36.9, 35.3, 34.2, 30.4, 25.1; ^{11}B NMR (96 MHz, CD_3Cl): δ 31.5 (s) (br); IR (neat) 2952, 1388, 1291, 1142, 974, 859, 697 cm^{-1}; HRMS ESI-Q-TOF calcd for $C_{20}H_{31}BO_2Na$ $[MNa]^+$ 337.2313, found 337.2317

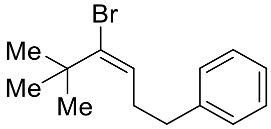

1g

(*E*)-(4-Bromo-5,5-dimethylhex-3-enyl)benzene (**1g**)**:** The title compound was obtained from **S4** (210 mg, 0.67 mmol), bromine (37.6 μL, 0.74 mmol) and NaOMe (2.45 mL, 0.6 M in MeOH) in anhydrous CH_2Cl_2 (3.4 mL, 0.2 M) according to the general procedure (Method **C**). The crude reaction mixture was purified by column chromatography on silica gel (2% Et_2O in hexanes, R_f = 0.42) to afford the desired product **1f** as a colorless liquid (49 mg, 27% yield, only *E*-isomer). The olefin geometry for compound **1g** was determined based on the data reported in literature[9]. ^{1}H NMR (300 MHz, $CDCl_3$) δ 7.34-7.30 (m, 2H), 7.26-7.19 (m, 3H), 5.99 (t, J = 7.5 Hz, 1H =CH_E), 2.74-2.69 (m, 2H), 2.60-2.52 (m, 2H), 1.28 (s, 9H); ^{13}C NMR (75 MHz, $CDCl_3$) δ 141.3, 138.3, 131.7, 128.6, 126.2, 40.8, 36.3, 32.6, 31.2; IR (neat) 2959, 1649, 1455, 1168, 1008, 747.3, 698.9 cm^{-1}; LRMS-EI (m/z) calcd for $C_{14}H_{19}Br$ $[M]^{+\cdot}$ 267.2047, $C_{14}H_{19}$ $[M-Br]^{+\cdot}$ 187.1481, found 187.1

Oxidative hydrolysis of bromoalkenes

Method D - General procedure for the oxidative hydrolysis of bromoalkenes (2a–g)

To a 2-dram vial were added HTIB (0.2 equiv) in anhydrous CH_3CN (0.1 M) followed by addition of *m*-CPBA (1.1 equiv) and *p*-TSA·H_2O (1.1 equiv.) The homogenous solution was stirred at rt for 5 minutes. Then bromoalkene (1.0 equiv) was added dropwise to the reaction mixture and let to vigorously stirred at rt for 24 h. The solution was then diluted with 5% $Na_2S_2O_3$: sat. $NaHCO_3$ (2.0 mL, 1:1) and extracted with three portions of Et_2O (3 × 3.0 mL). The combined organic layers were washed with brine, dried over magnesium sulfate, filtered and concentrated by rotatory evaporation. The crude was purified by flash column chromatography (10–50% CH_2Cl_2 in hexanes or pentanes) to obtain the pure α–bromoketones (**2a**–**g**).

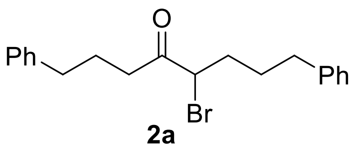

2a

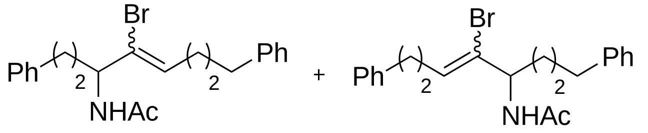

3a + 3a'

5-Bromo-1,8-diphenyloctan-4-one (**2a)**: The title compound was obtained from **1a** (68.7 mg, 0.2 mmol), HTIB (16.7 mg, 20 mol %), *p*-TSA·H_2O (7.6 mg, 20 mol %) and *m*CPBA (59.2 mg, 0.24 mmol, ca. 70%) in CH_3CN (2.0 mL, 0.1 M) according to the general procedure (Method **D**). The crude reaction mixture was purified by column chromatography on silica gel (40% CH_2Cl_2 in hexanes, R_f = 0.5) to afford the desired product **2a** as a colorless liquid (36.6 mg, 54% yield). Chromatographic elution by using 5% MeOH in CH_2Cl_2 afforded a mixture of Ritter-type side products as a light yellow liquid (19.2 mg, 24% yield) (**3a** + **3a**');

2a: ^{1}H NMR (300 MHz, $CDCl_3$) δ 7.33-7.23 (m, 4H), 7.20-7.17 (m, 6H), 4.22 (dd, J = 8.0, 6.4 Hz, 1H), 2.80-2.54 (m, 6H), 2.07-1.87 (m, 4H), 1.85-1.75 (m, 1H), 1.70-1.58 (m, 1H); ^{13}C NMR (75 MHz, $CDCl_3$) δ 203.9, 141.5, 141.4, 128.6, 128.5, 128.4, 128.3, 53.5, 38.4, 35.3, 35.0, 32.9, 29.2, 25.5; IR (neat) 2929, 1712, 1496, 1453, 1029, 743, 697 cm^{-1}; HRMS ESI-Q-TOF calcd for $C_{20}H_{23}BrONa$ $[MNa]^+$ 381.0824, found 381.0833

3a + **3a**': *N*-(5-bromo-1,8-diphenyloct-5-en-4-yl)acetamide and *N*-(4-bromo-1,8-diphenyloct-4-en-3-yl)acetamide; ^{1}H NMR (300 MHz, $CDCl_3$) δ 7.31-7.26 (m, 4H), 7.23-7.13 (m, 10H), 6.01 (q, J = 6.7 Hz, 1H), 5.65 (dd, J = 19.3, 8.8 Hz, 1H), 4.57 (td, J = 16.3, 7.9 Hz, 1H), 2.73-2.56 (m, 6H), 2.53-2.41 (m, 2H), 2.27-2.12 (m, 2H), 1.97 (s, 3H), 1.93-1.88 (m, 2 H), 1.80-1.63 (m, 2H), 1.61-1.49 (m, 3H); ^{13}C NMR (75 MHz, $CDCl_3$) δ 169.7, 169.1, 169.0, 142.0, 141.9, 141.1, 141.0, 132.0, 131.0, 129.3, 128.8, 128.7, 128.6, 128.5, 128.4, 128.3, 126.2, 126.1, 126.0, 126.9, 35.7, 35.6, 35.5, 34.4, 33.4, 32.6, 32.3, 30.6, 30.1, 29.8, 27.6, 23.6; IR (neat) 3515, 2973, 1642, 1374, 746, 689 cm^{-1}; HRMS ESI-Q-TOF calcd for $C_{22}H_{26}BrNa$ $[MNa]^+$ 422.1095, found 422.1096

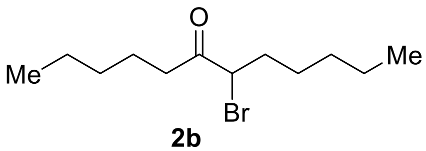

2b

7-Bromododecan-6-one (**2b)**: The title compound was obtained from **1b** (49.4 mg, 0.2 mmol), HTIB (16.7 mg, 20 mol %), *p*-TSA·H_2O (7.6 mg, 20 mol %) and *m*CPBA (59.2 mg, 0.24 mmol, ca. 70%) in CH_3CN (2.0 mL, 0.1 M) according to the general procedure (Method **D**). The crude reaction mixture was purified by column chromatography on silica gel (20% CH_2Cl_2 in hexanes, R_f = 0.55) to afford the desired product **2b** as a colorless liquid (26.0 mg, 49% yield); ^{1}H NMR (300 MHz, $CDCl_3$) δ 4.23 (dd, *J* = 7.9, 6.6 Hz, 1H), 2.76-2.56 (m, 2H), 2.05-1.84 (m, 2H), 1.66-1.57 (m, 2H), 1.39-1.22 (m, 10H), 0.95-0.87 (m, 6H); ^{13}C NMR (75 MHz, $CDCl_3$) δ 204.6, 53.9, 39.1, 33.6, 31.4, 31.3, 27.1, 23.8, 22.6, 22.5, 14.1, 14.0; IR (neat) 2912, 2855, 1708, 1418, 1353, 711 cm^{-1}; HRMS ESI-Q-TOF calcd for $C_{12}H_{23}BrONa$ $[MNa]^+$ 285.0824, found 285.0824

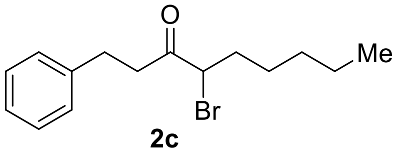

2c

4-Bromo-1-phenylnonan-3-one (**2c)**: The title compound was obtained from **1c** (56.2 mg, 0.2 mmol), HTIB (16.7 mg, 20 mol %), *p*-TSA·H_2O (7.6 mg, 20 mol %) and *m*CPBA (59.2 mg, 0.24 mmol, ca. 70%) in CH_3CN (2.0 mL, 0.1 M) according to the general procedure (Method **D**). The crude reaction mixture was purified by column chromatography on silica gel (40% CH_2Cl_2 in hexanes, R_f = 0.52) to afford the desired product **2c** as a colorless liquid (28.0 mg, 47% yield); ^{1}H NMR (300 MHz, $CDCl_3$) δ 7.32-7.26 (m, 2H), 7.22-7.19 (m, 3H), 4.19 (dd, *J* = 7.9, 6.6 Hz, 1H), 3.09-2.91 (m, 4H), 1.96-1.86 (m, 2H), 1.44-1.26 (m, 6H), 0.88 (t, *J* = 6.7 Hz, 3H); ^{13}C NMR (75 MHz, $CDCl_3$) δ 203.6, 140.7, 128.6, 128.5, 126.4, 54.1, 40.7, 33.4, 31.2, 30.3, 27.1, 22.5, 14.1; IR (neat) 2928, 1715, 1496, 1454, 1073, 748, 697 cm^{-1}; HRMS ESI-Q-TOF calcd for $C_{15}H_{21}BrONa$ $[MNa]^+$ 319.0668, found 319.0670

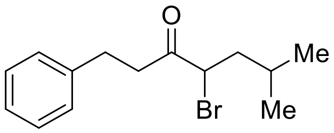

2d

4-Bromo-6-methyl-1-phenylheptan-3-one (**2d**): The title compound was obtained from **1d** (53.4 mg, 0.2 mmol), HTIB (16.7 mg, 20 mol %), *p*-TSA·H_2O (7.6 mg, 20 mol %) and *m*-CPBA (59.2 mg, 0.24 mmol, ca. 70%) in CH_3CN (2.0 mL, 0.1 M) according to the general procedure

(Method **D**). The crude reaction mixture was purified by column chromatography on silica gel (40% CH_2Cl_2 in hexanes, R_f = 0.48) to afford the desired product **2d** as a colorless liquid (28.3 mg, 50% yield); ^{1}H NMR (300 MHz, $CDCl_3$) δ 7.32-7.26 (m, 2H), 7.22-7.19 (m, 3H), 4.26 (dd, J = 8.7, 5.9 Hz, 1H), 3.10-2.92 (m, 4H), 1.87-1.66 (m, 3H), 0.89 (dd, J = 18.2, 6.2 Hz, 6H); ^{13}C NMR (75 MHz, $CDCl_3$) δ 203.6, 140.7, 128.7, 128.5, 126.4, 52.6, 41.9, 40.6, 31.3, 26.3, 22.7, 21.6; IR (neat) 2958, 1714, 1454, 1369, 1073, 748, 697 cm^{-1}; HRMS ESI-Q-TOF calcd for $C_{14}H_{19}BrONa$ $[MNa]^+$ 305.0511, found 305.0513

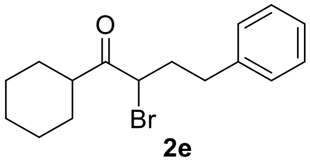

2e

2-Bromo-1-cyclohexyl-4-phenylbutan-1-one (**2e**): The title compound was obtained from **1e** (58.6 mg, 0.2 mmol), HTIB (16.7 mg, 20 mol %), *p*-TSA·H_2O (7.6 mg, 20 mol %) and *m*CPBA (59.2 mg, 0.24 mmol, ca. 70%) in CH_3CN (2.0 mL, 0.1 M) according to the general procedure (Method **D**). The crude reaction mixture was purified by column chromatography on silica gel (30% CH_2Cl_2 in hexanes, R_f = 0.45) to afford the desired product **2e** as a colorless liquid (30.9 mg, 50% yield); ^{1}H NMR (300 MHz, $CDCl_3$) δ 7.33-7.26 (m, 2H), 7.24-7.18 (m, 3H), 4.32 (dd, J = 8.0, 6.3 Hz, 1H), 2.83-2.64 (m, 3H), 2.36-2.20 (m, 2H), 1.85-1.66 (m, 5H), 1.57-1.45 (m, 1H), 1.34-1.18 (m, 4H); ^{13}C NMR (75 MHz, $CDCl_3$) δ 206.7, 140.3, 128.8, 128.6, 126.5, 51.2, 48.2, 34.7, 33.4, 29.8, 28.9, 25.9, 25.8, 25.4; IR (neat) 2929, 2855, 1710, 1497, 1449, 986, 749, 697 cm^{-1}; HRMS ESI-Q-TOF calcd for $C_{16}H_{21}BrONa$ $[MNa]^+$ 331.0668, found 331.0670

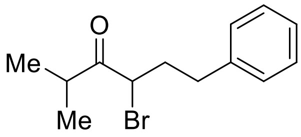

2f

4-Bromo-2-methyl-6-phenylhexan-3-one (**2f**): The title compound was obtained from **1f** (50.6 mg, 0.2 mmol), HTIB (16.7 mg, 20 mol %), *p*-TSA·H_2O (7.6 mg, 20 mol %) and *m*CPBA (59.2 mg, 0.24 mmol, ca. 70%) in CH_3CN (2.0 mL, 0.1 M) according to the general procedure (Method **D**). The crude reaction mixture was purified by column chromatography on silica gel (30% CH_2Cl_2 in hexanes, R_f = 0.6) to afford the desired product **2f** as a colorless liquid (26.9 mg, 50% yield); ^{1}H NMR (300 MHz, $CDCl_3$) δ 7.34-7.28 (m, 2H), 7.26-7.19 (m, 3H), 4.34 (dd, J = 8.0, 6.3 Hz, 1H), 3.02 (dt, J = 13.7, 6.9 Hz, 1H), 2.85-2.65 (m, 2H), 2.40-2.19 (m, 2H), 1.17 (d, J = 6.7 Hz, 3H), 1.09 (d, J = 7.0 Hz, 3H); ^{13}C NMR (75 MHz, $CDCl_3$) δ 207.6, 140.2, 128.7,

128.6, 126.5, 50.9, 33.2, 34.7, 33.4, 19.5, 18.8; IR (neat) 2971, 1716, 1497, 1455, 1001, 748, 698 cm^{-1}; HRMS ESI-Q-TOF calcd for $C_{13}H_{13}BrONa$ $[MNa]^+$ 291.0355, found 291.0357

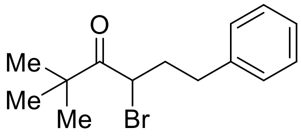

2g

4-Bromo-2,2-dimethyl-6-phenylhexan-3-one (**2g)**: The title compound was obtained from **1g** (40.0 mg, 0.15 mmol), HTIB (12.5 mg, 20 mol %), *p*-TSA·H_2O (5.7 mg, 20 mol %) and *m*-CPBA (44.3 mg, 0.18 mmol, ca. 70%) in CH_3CN (1.5 mL, 0.1 M) according to the general procedure (Method **D**). The crude reaction mixture was purified by column chromatography on silica gel (30% CH_2Cl_2 in hexanes, R_f = 0.56) to afford the desired product **2g** as a colorless liquid (18.5 mg, 44% yield); ^1H NMR (300 MHz, $CDCl_3$) δ 7.36-7.29 (m, 2H), 7.27-7.21 (m, 3H), 4.59 (dd, J = 8.2, 6.0 Hz, 1H), 2.86-2.66 (m, 2H), 2.37-2.21 (m, 2H); ^{13}C NMR (75 MHz, $CDCl_3$) δ 209.1, 140.3, 128.7, 128.5, 126.5, 44.9, 44.5, 35.8, 33.4, 26.8; IR (neat) 2968, 1709, 1455, 1369, 978, 750, 699 cm^{-1}; HRMS ESI-Q-TOF calcd for $C_{14}H_{19}BrONa$ $[MNa]^+$ 305.0511, found 305.0511

NMR Spectras

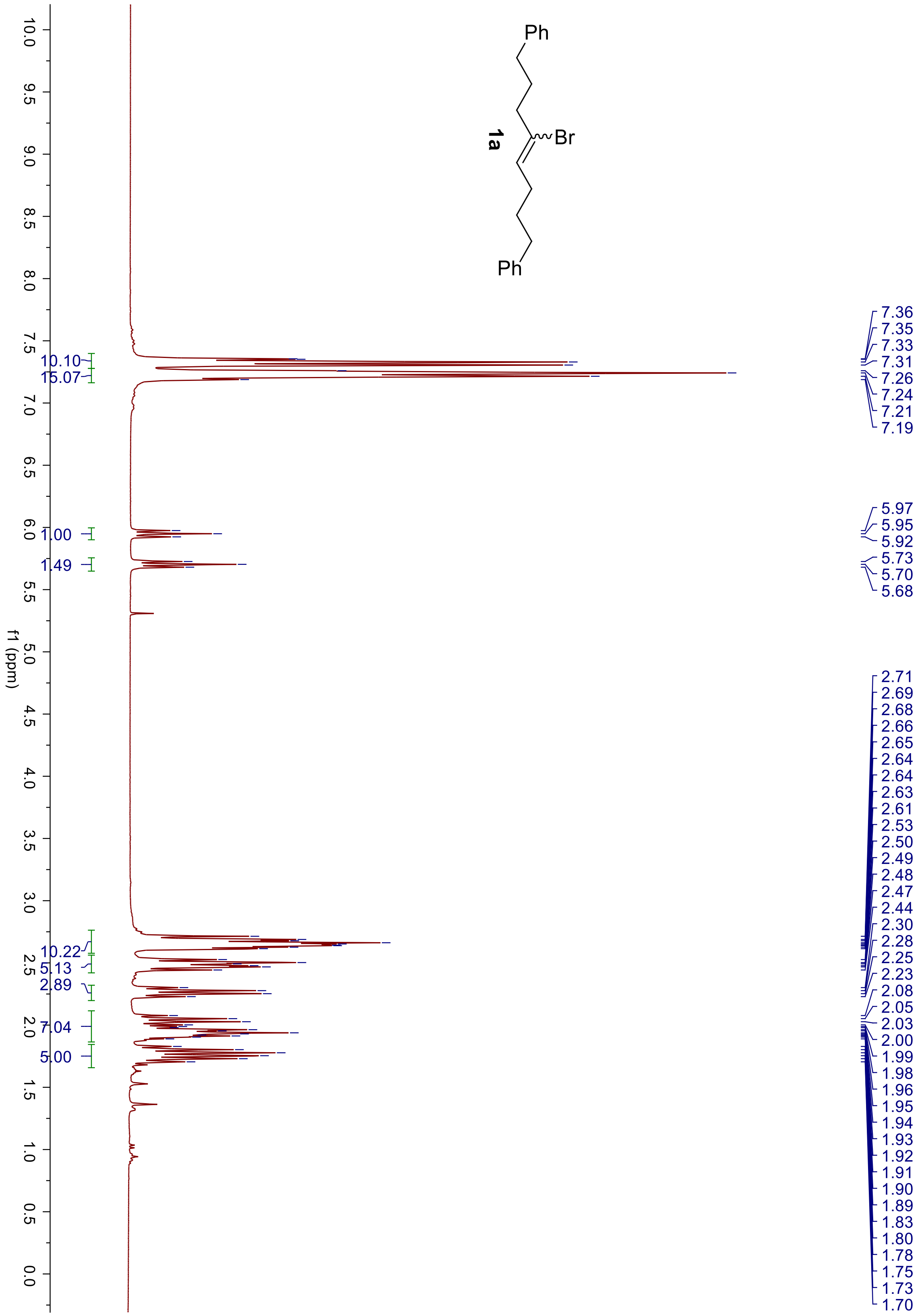

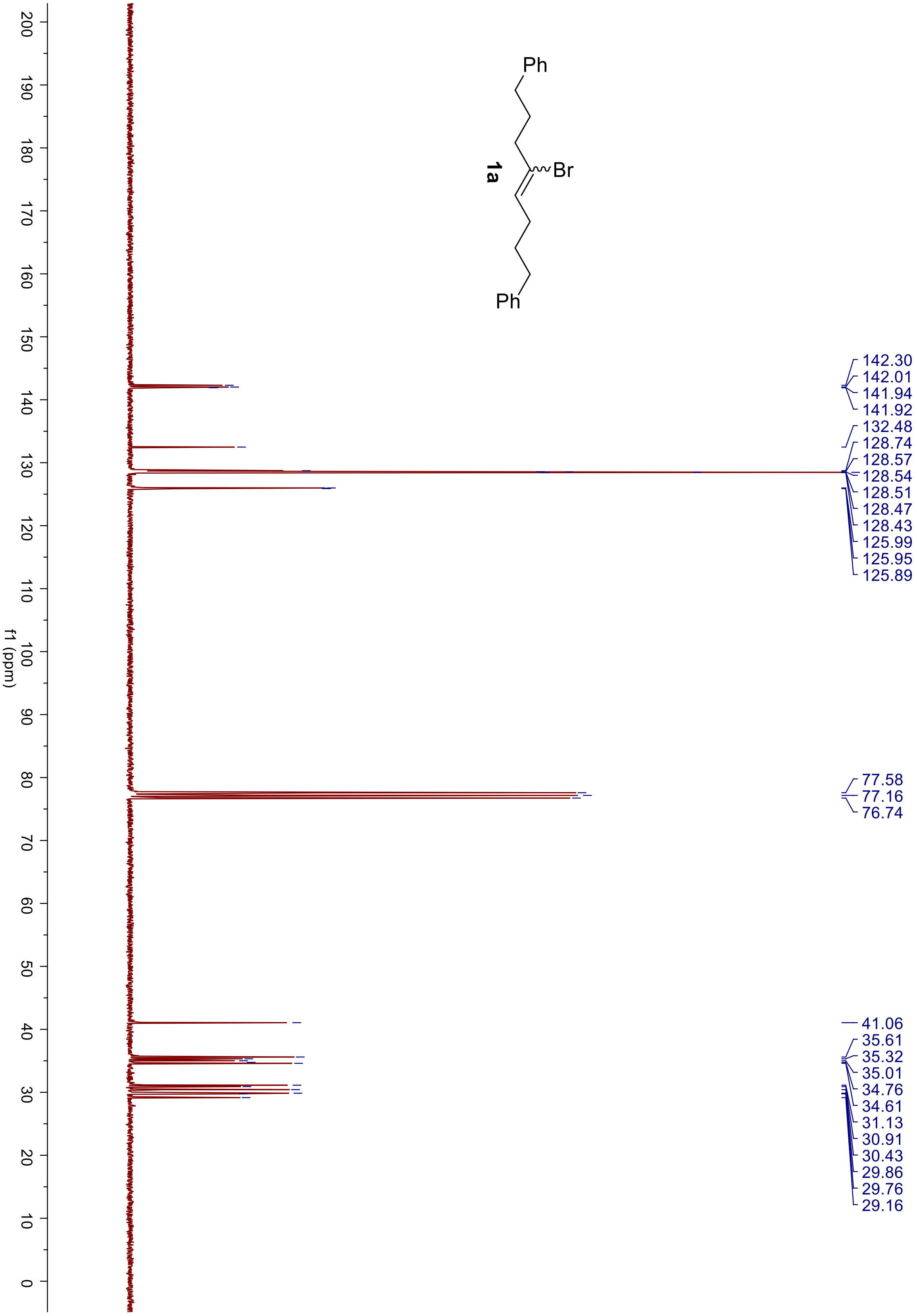

Ph
Br
1a
Ph
142.30
142.01
141.94
141.92
132.48
128.74
128.57
128.54
128.51
128.47
128.43
125.99
125.95
125.89
77.58
77.16
76.74
41.06
35.61
35.32
35.01
34.76
34.61
31.13
30.91
30.43
29.86
29.76
29.16
f1 (ppm)

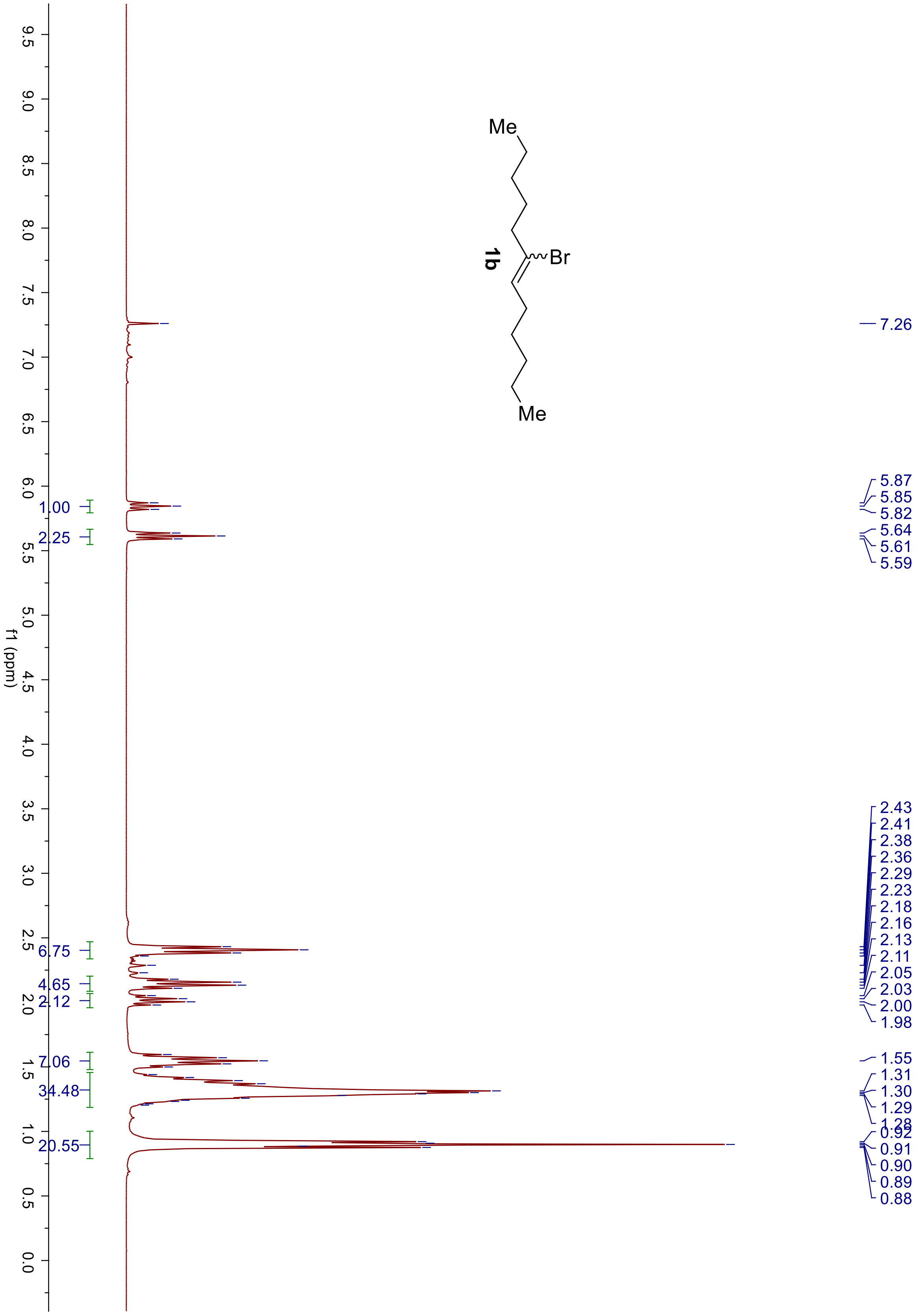

Me
Br
1b
Me
7.26
5.87
5.85
5.82
5.64
5.61
5.59
2.43
2.41
2.38
2.36
2.29
2.23
2.18
2.16
2.13
2.11
2.05
2.03
2.00
1.98
1.55
1.31
1.30
1.29
1.28
0.92
0.91
0.90
0.89
0.88
1.00
2.25
6.75
4.65
2.12
7.06
34.48
20.55
f1 (ppm)

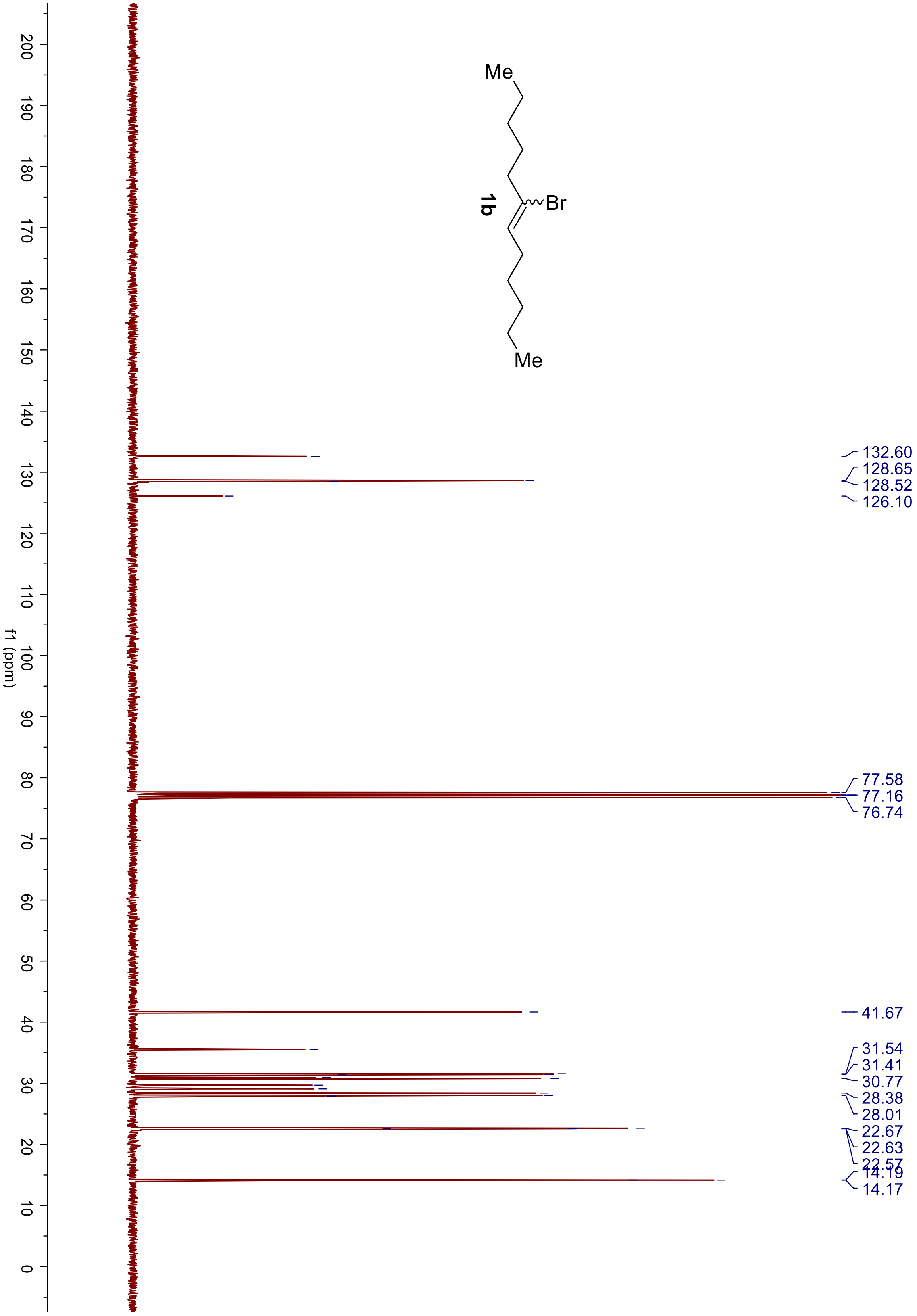
Me
Br
1b
Me
132.60
128.65
128.52
126.10
77.58
77.16
76.74
41.67
31.54
31.41
30.77
28.38
28.01
22.67
22.63
22.57
14.19
14.17
f1 (ppm)

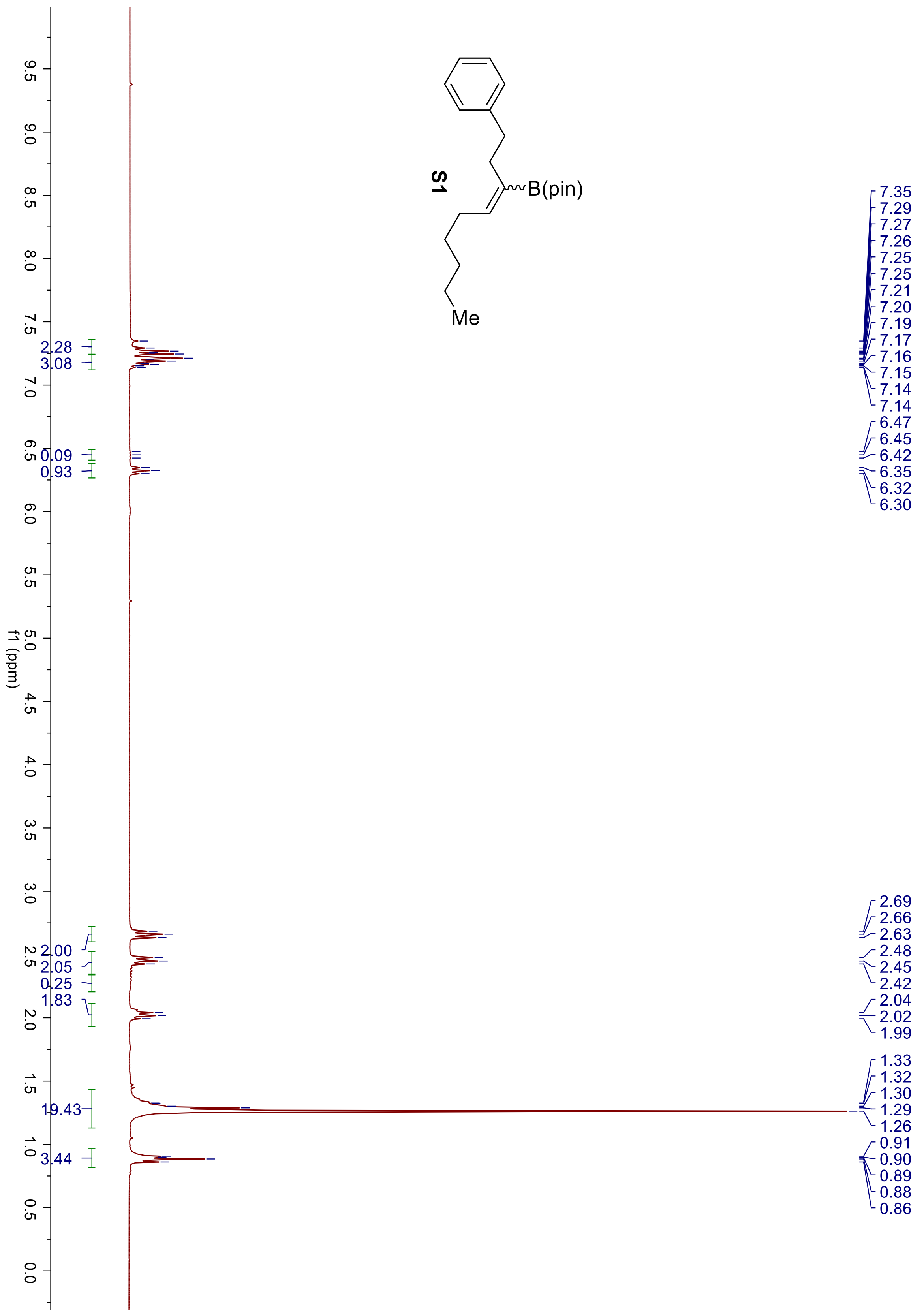
S1
B(pin)
Me
f1 (ppm)

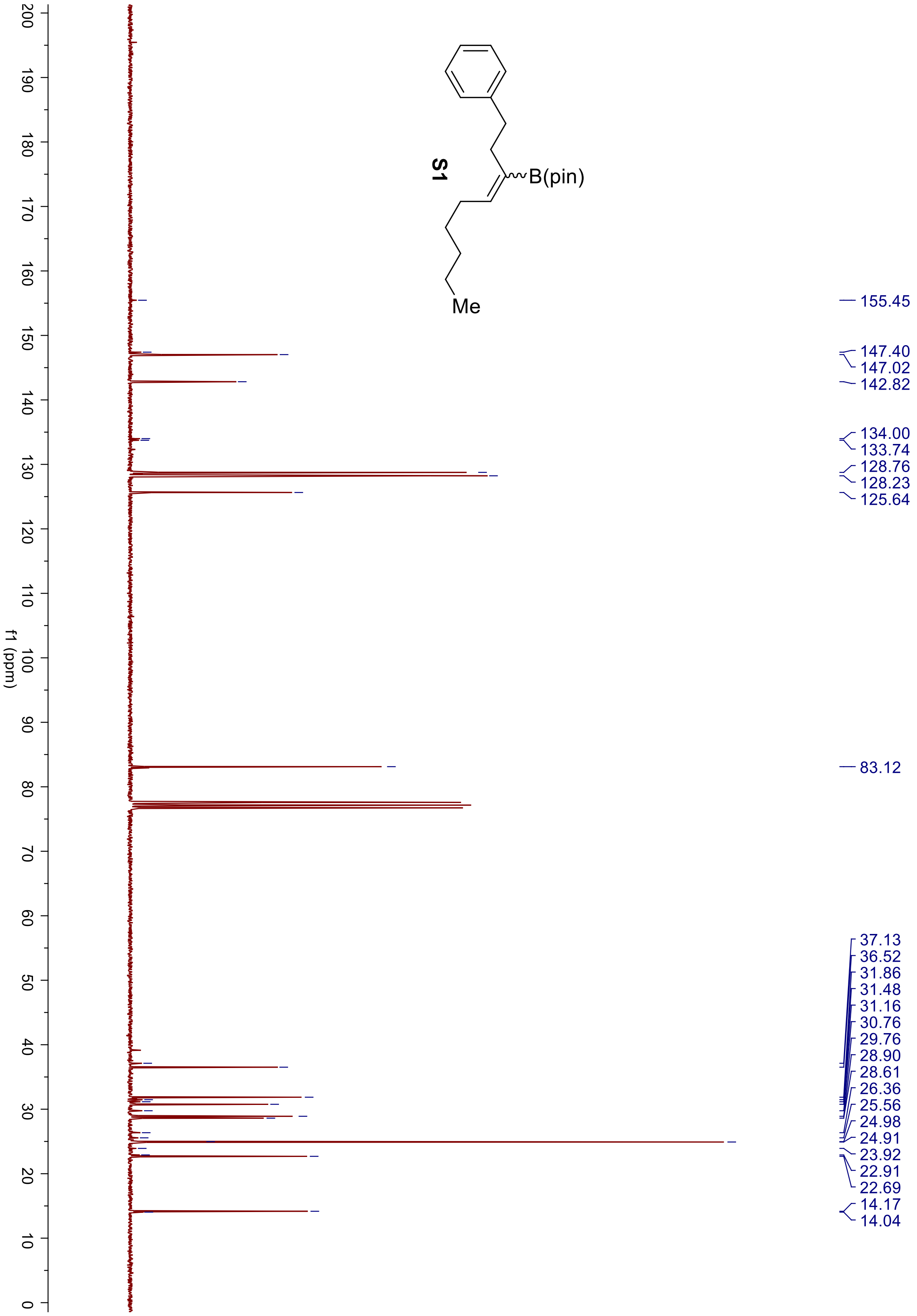
S1
B(pin)
Me
f1 (ppm)
155.45
147.40
147.02
142.82
134.00
133.74
128.76
128.23
125.64
83.12
37.13
36.52
31.86
31.48
31.16
30.76
29.76
28.90
28.61
26.36
25.56
24.98
24.91
23.92
22.91
22.69
14.17
14.04

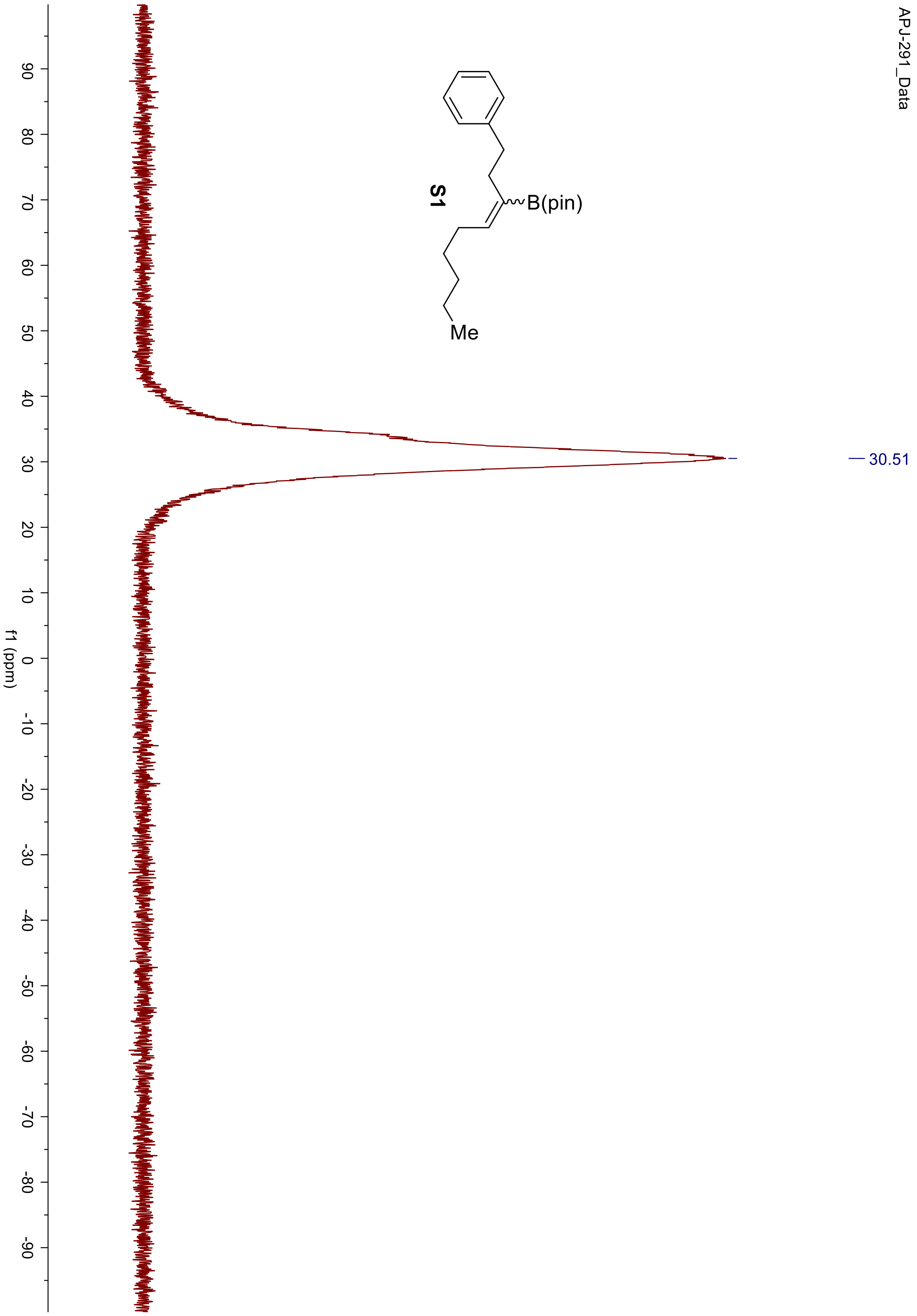

APJ-291_Data
B(pin)
Me
S1
30.51
f1 (ppm)

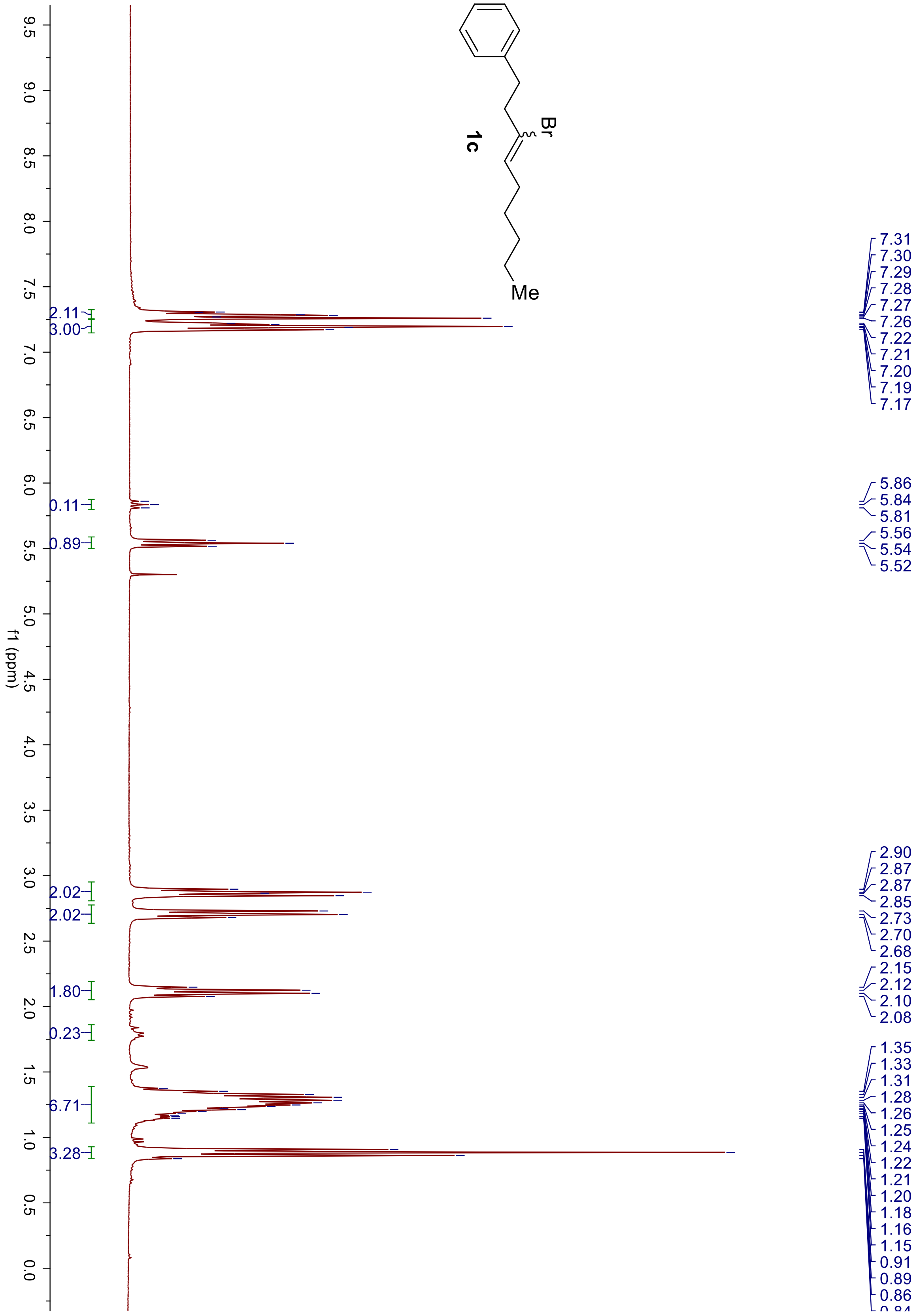
Br
1c
Me
f1 (ppm)
9.5
9.0
8.5
8.0
7.5
7.0
6.5
6.0
5.5
5.0
4.5
4.0
3.5
3.0
2.5
2.0
1.5
1.0
0.5
0.0
2.11
3.00
0.11
0.89
2.02
2.02
1.80
0.23
6.71
3.28
7.31
7.30
7.29
7.28
7.27
7.26
7.22
7.21
7.20
7.19
7.17
5.86
5.84
5.81
5.56
5.54
5.52
2.90
2.87
2.87
2.85
2.73
2.70
2.68
2.15
2.12
2.10
2.08
1.35
1.33
1.31
1.28
1.26
1.25
1.24
1.22
1.21
1.20
1.18
1.16
1.15
0.91
0.89
0.86

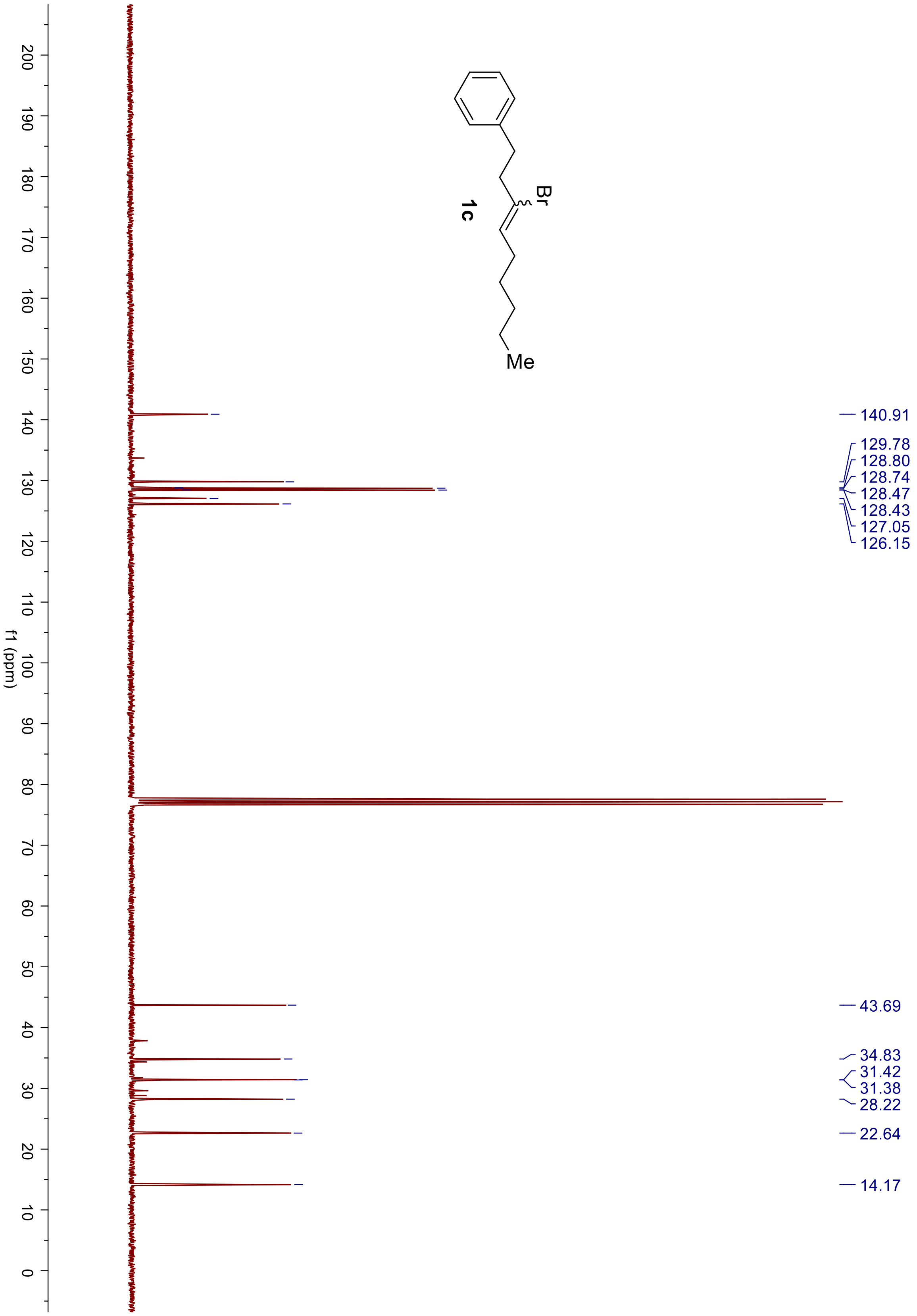

Br
1c
Me
140.91
129.78
128.80
128.74
128.47
128.43
127.05
126.15
43.69
34.83
31.42
31.38
28.22
22.64
14.17
f1 (ppm)

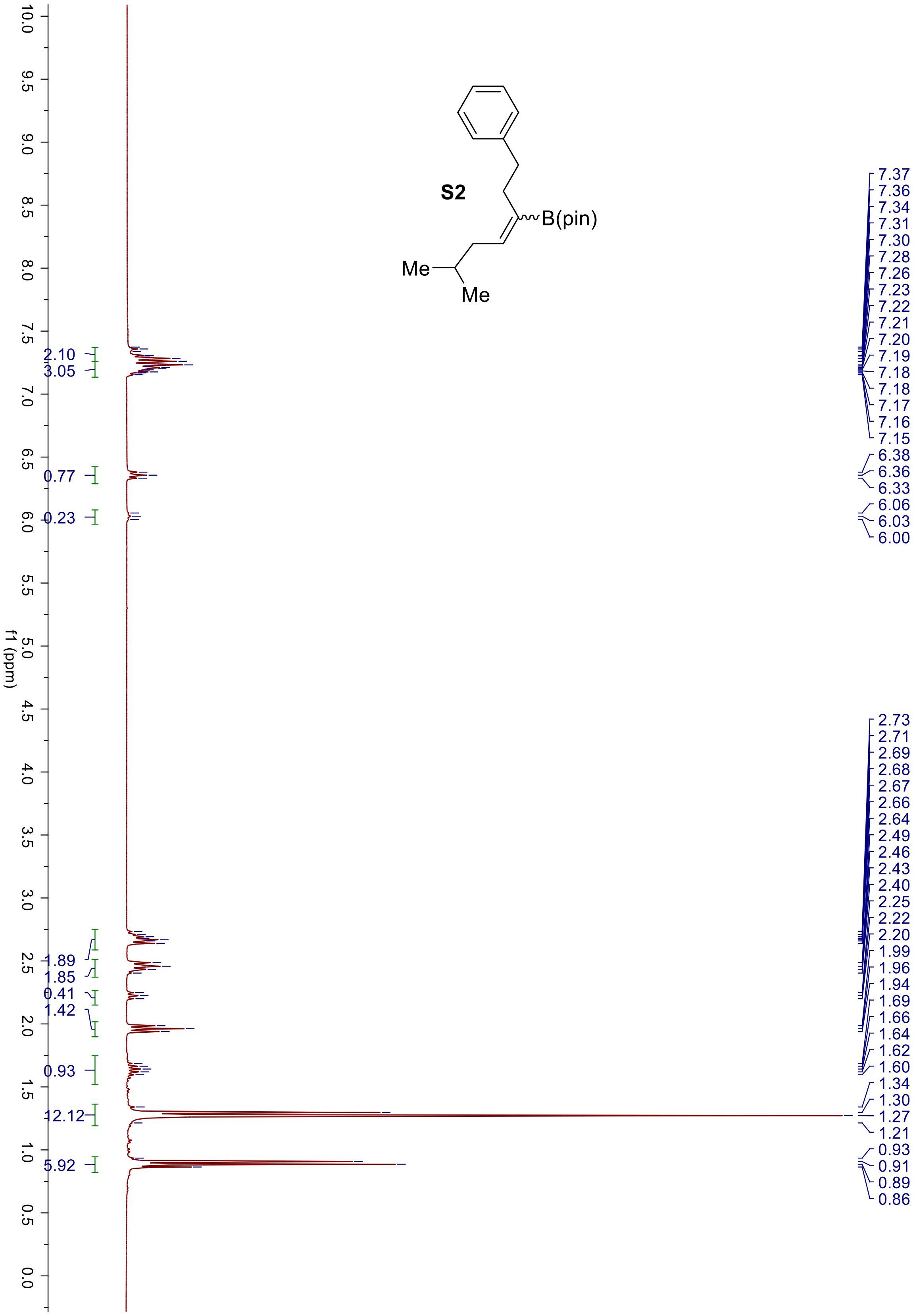
S2
B(pin)
Me
Me
f1 (ppm)
10.0 9.5 9.0 8.5 8.0 7.5 7.0 6.5 6.0 5.5 5.0 4.5 4.0 3.5 3.0 2.5 2.0 1.5 1.0 0.5 0.0
2.10
3.05
0.77
0.23
1.89
1.85
0.41
1.42
0.93
12.12
5.92
7.37 7.36 7.34 7.31 7.30 7.28 7.26 7.23 7.22 7.21 7.20 7.19 7.18 7.18 7.17 7.16 7.15
6.38 6.36 6.33
6.06 6.03 6.00
2.73 2.71 2.69 2.68 2.67 2.66 2.64 2.49 2.46 2.43 2.40 2.25 2.22 2.20 1.99 1.96 1.94 1.69 1.66 1.64 1.62 1.60 1.34 1.30 1.27 1.21
0.93 0.91 0.89 0.86

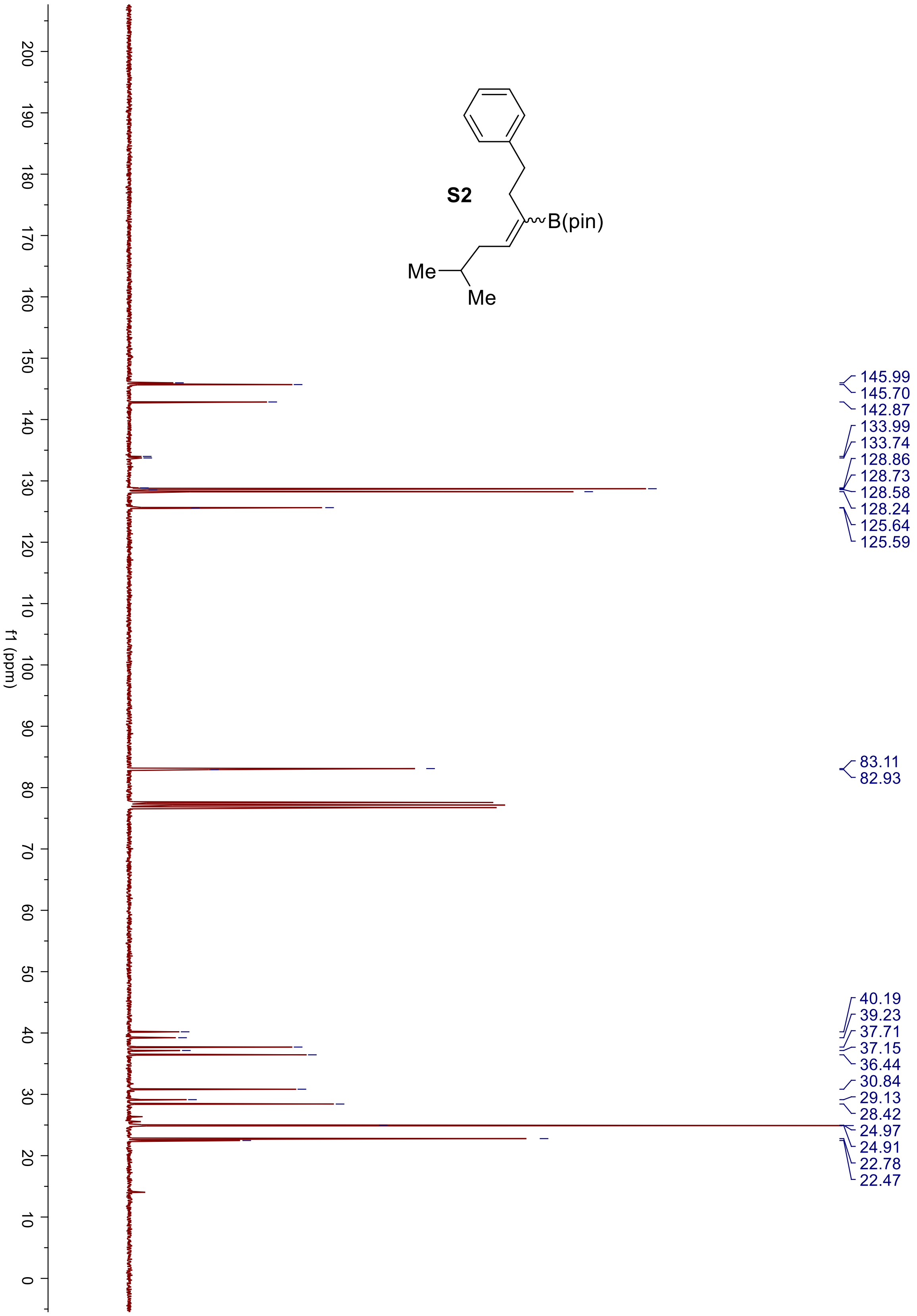

S2
B(pin)
Me
Me
145.99
145.70
142.87
133.99
133.74
128.86
128.73
128.58
128.24
125.64
125.59
83.11
82.93
40.19
39.23
37.71
37.15
36.44
30.84
29.13
28.42
24.97
24.91
22.78
22.47
f1 (ppm)
200
190
180
170
160
150
140
130
120
110
100
90
80
70
60
50
40
30
20
10
0

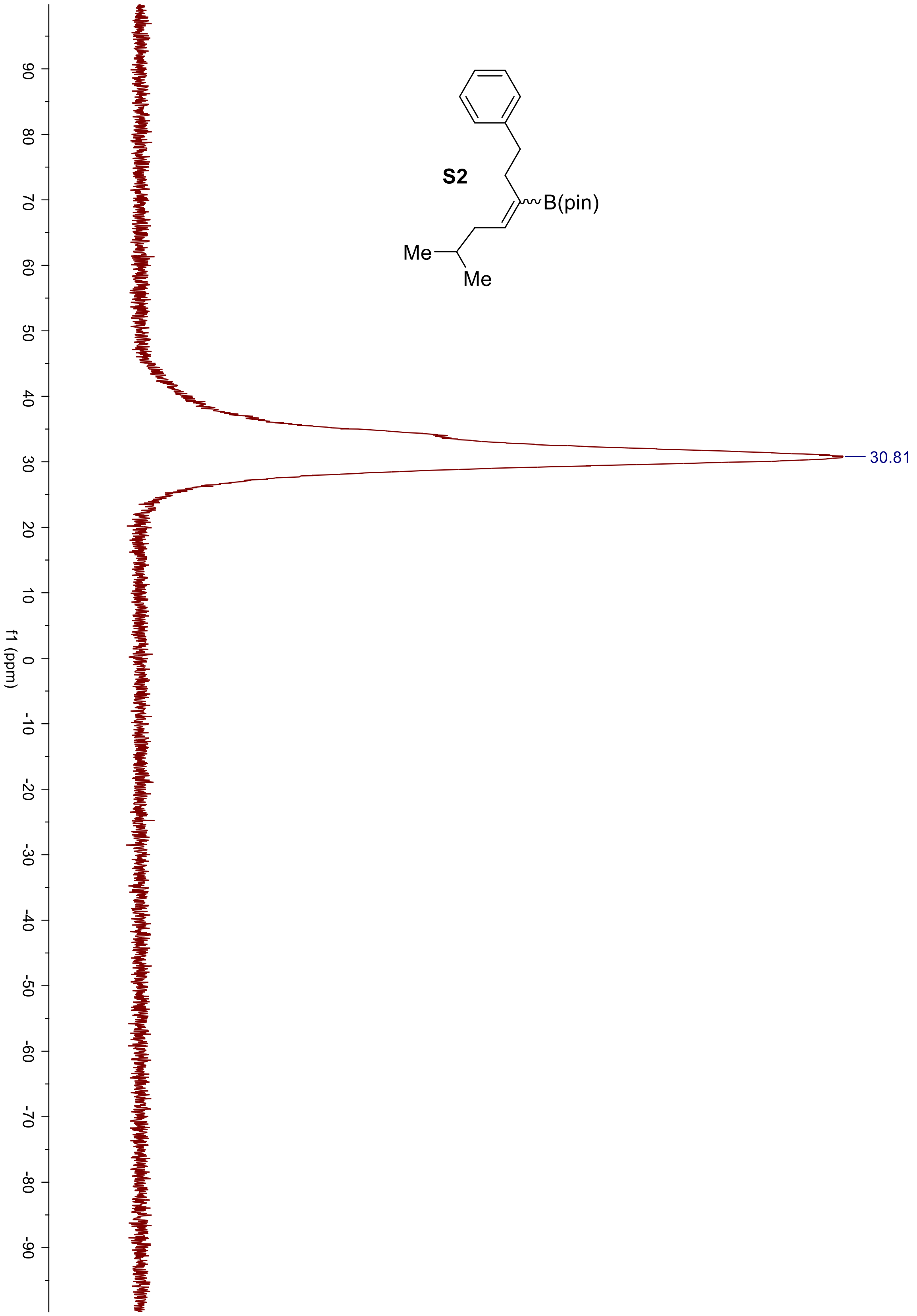
S2
B(pin)
Me
Me
30.81
f1 (ppm)
90
80
70
60
50
40
30
20
10
0
-10
-20
-30
-40
-50
-60
-70
-80
-90

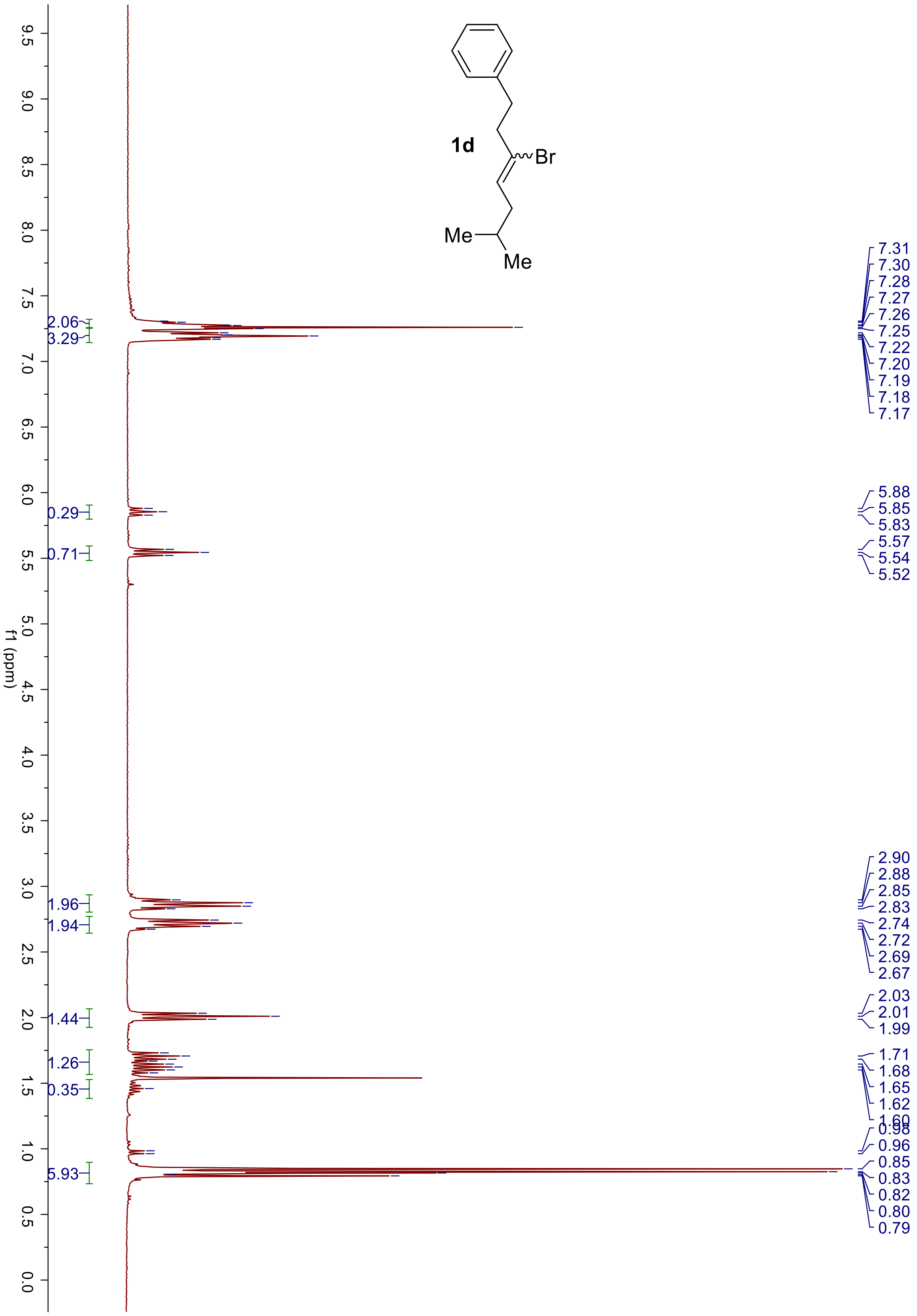
1d
Br
Me
Me
f1 (ppm)
7.31
7.30
7.28
7.27
7.26
7.25
7.22
7.20
7.19
7.18
7.17
5.88
5.85
5.83
5.57
5.54
5.52
2.90
2.88
2.85
2.83
2.74
2.72
2.69
2.67
2.03
2.01
1.99
1.71
1.68
1.65
1.62
0.98
0.96
0.85
0.83
0.82
0.80
0.79
2.06
3.29
0.29
0.71
1.96
1.94
1.44
1.26
0.35
5.93

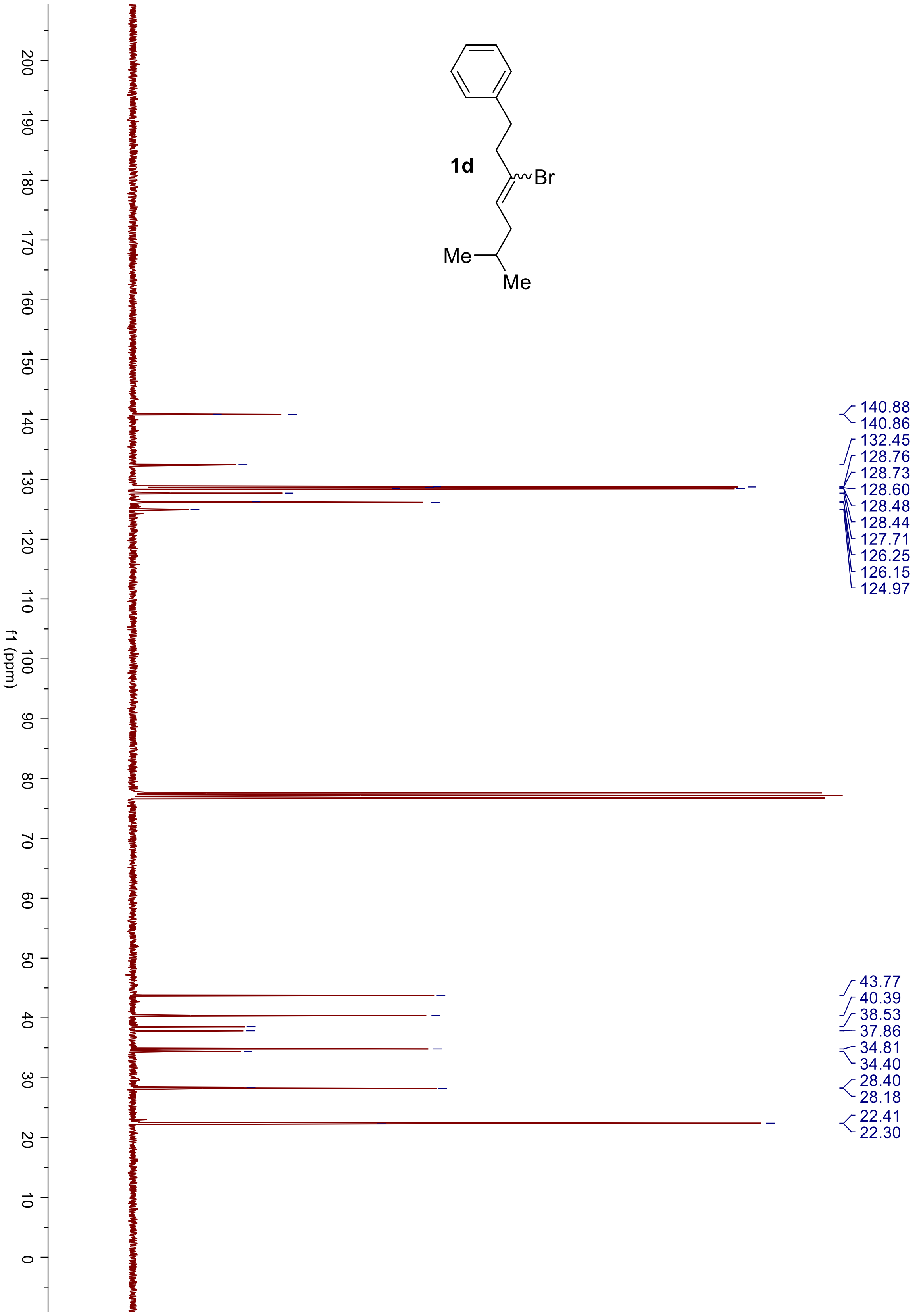
1d
Br
Me
Me
140.88
140.86
132.45
128.76
128.73
128.60
128.48
128.44
127.71
126.25
126.15
124.97
43.77
40.39
38.53
37.86
34.81
34.40
28.40
28.18
22.41
22.30
200
190
180
170
160
150
140
130
120
110
100
90
80
70
60
50
40
30
20
10
0
f1 (ppm)

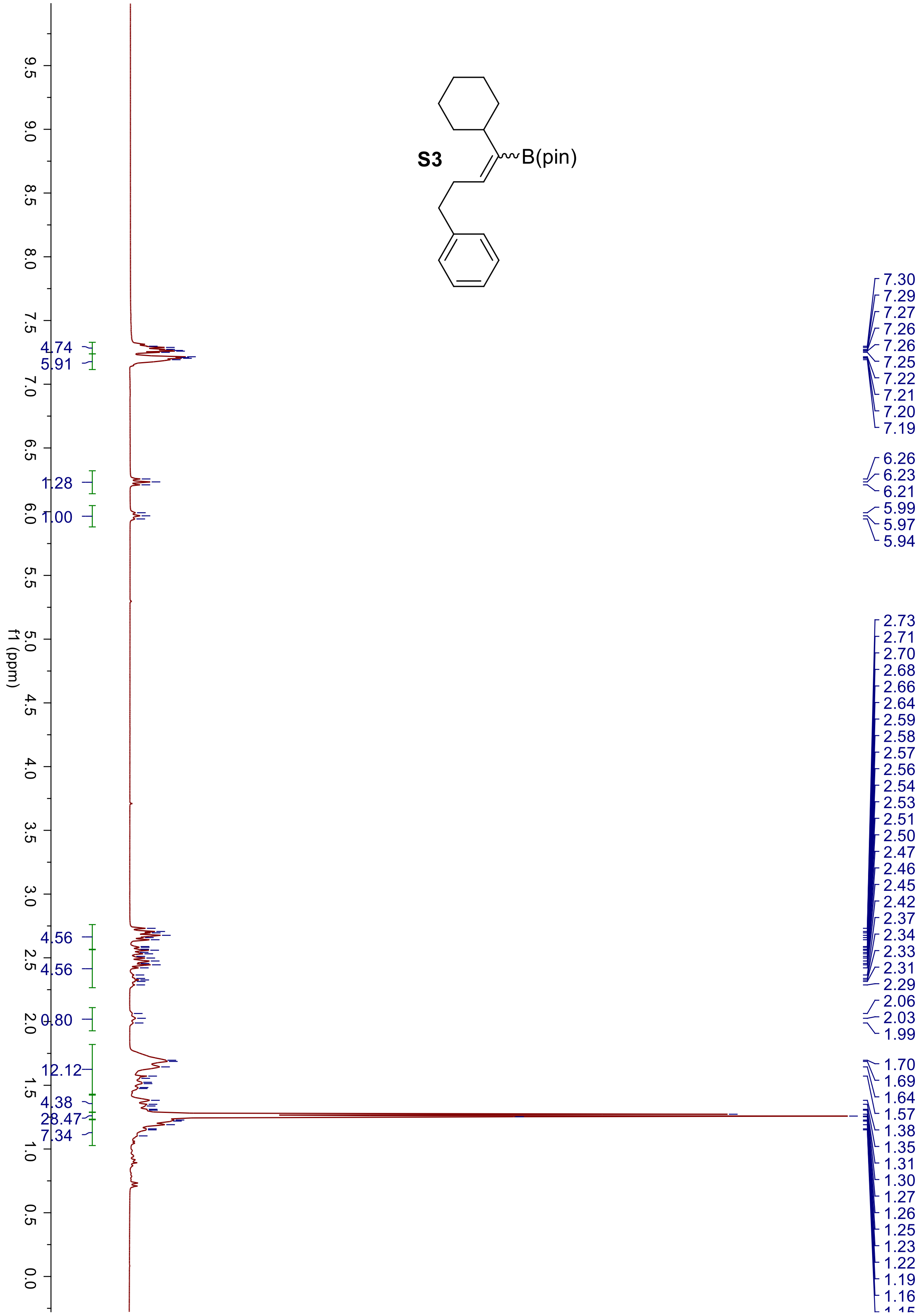

S3
B(pin)
f1 (ppm)
9.5
9.0
8.5
8.0
7.5
7.0
6.5
6.0
5.5
5.0
4.5
4.0
3.5
3.0
2.5
2.0
1.5
1.0
0.5
0.0
4.74
5.91
1.28
1.00
4.56
4.56
0.80
12.12
4.38
28.47
7.34
7.30
7.29
7.27
7.26
7.26
7.25
7.22
7.21
7.20
7.19
6.26
6.23
6.21
5.99
5.97
5.94
2.73
2.71
2.70
2.68
2.66
2.64
2.59
2.58
2.57
2.56
2.54
2.53
2.51
2.50
2.47
2.46
2.45
2.42
2.37
2.34
2.33
2.31
2.29
2.06
2.03
1.99
1.70
1.69
1.64
1.57
1.38
1.35
1.31
1.30
1.27
1.26
1.25
1.23
1.22
1.19
1.16

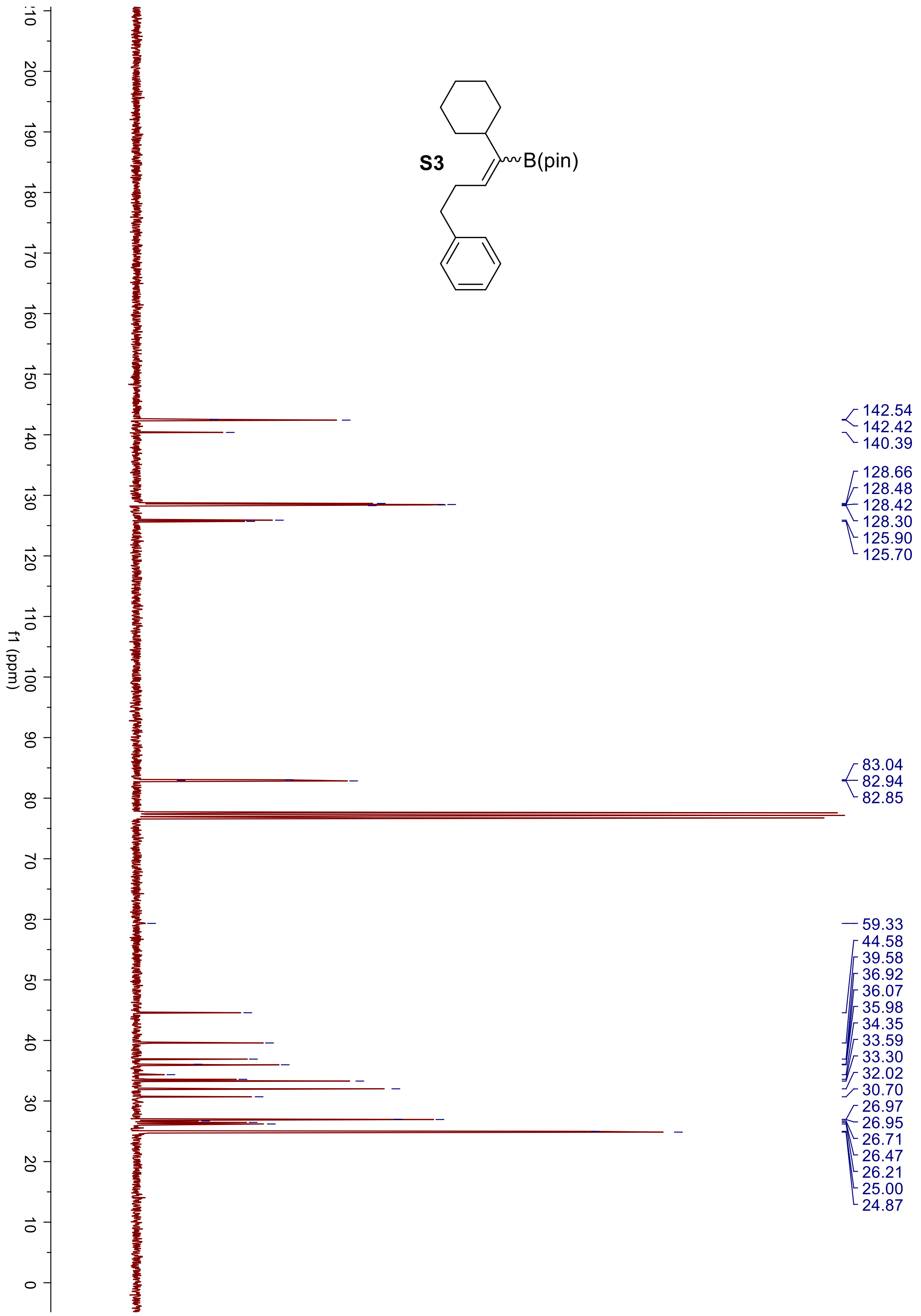
S3
B(pin)
142.54
142.42
140.39
128.66
128.48
128.42
128.30
125.90
125.70
83.04
82.94
82.85
59.33
44.58
39.58
36.92
36.07
35.98
34.35
33.59
33.30
32.02
30.70
26.97
26.95
26.71
26.47
26.21
25.00
24.87
f1 (ppm)
200
190
180
170
160
150
140
130
120
110
100
90
80
70
60
50
40
30
20
10
0

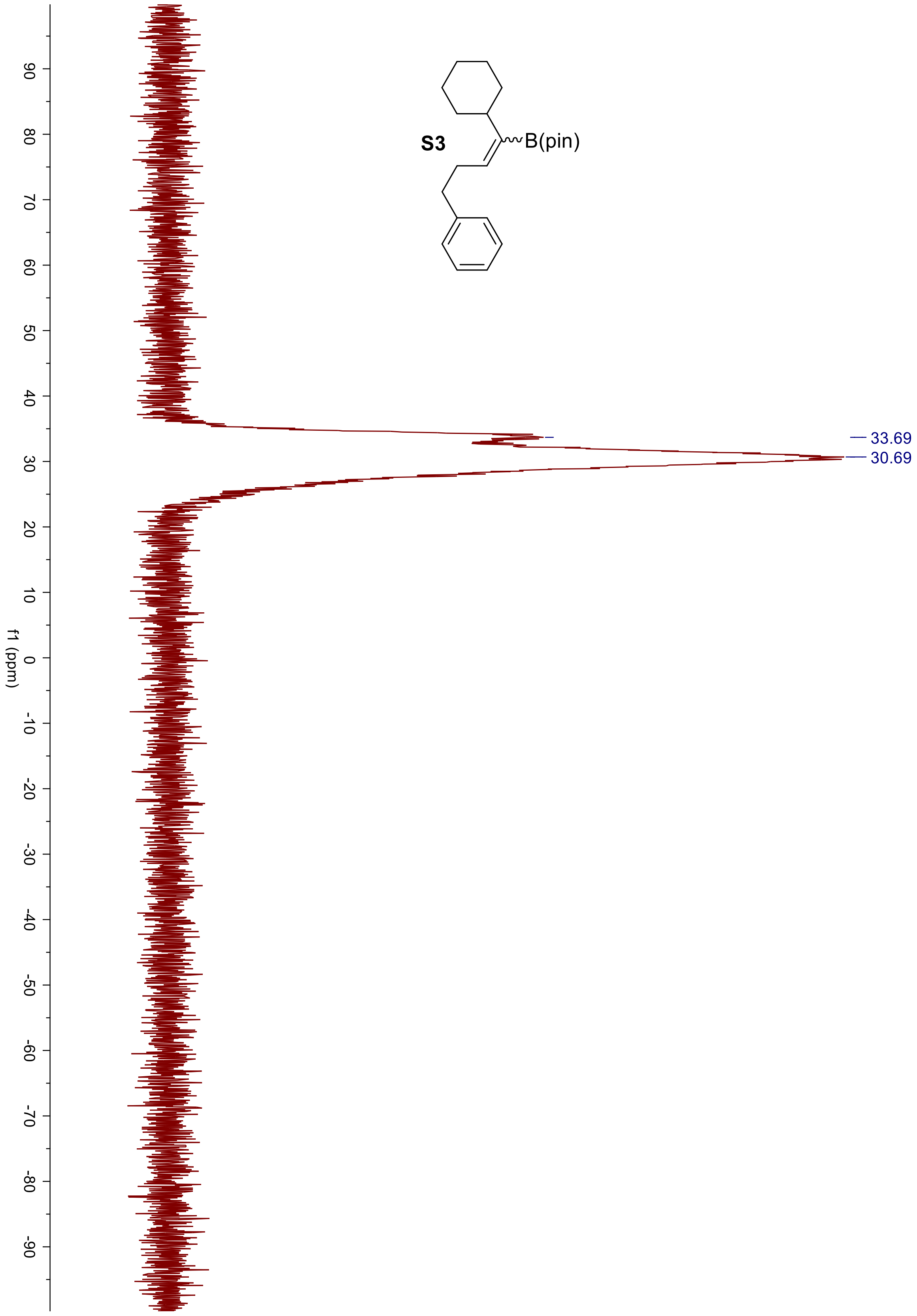
S3
B(pin)
33.69
30.69
f1 (ppm)

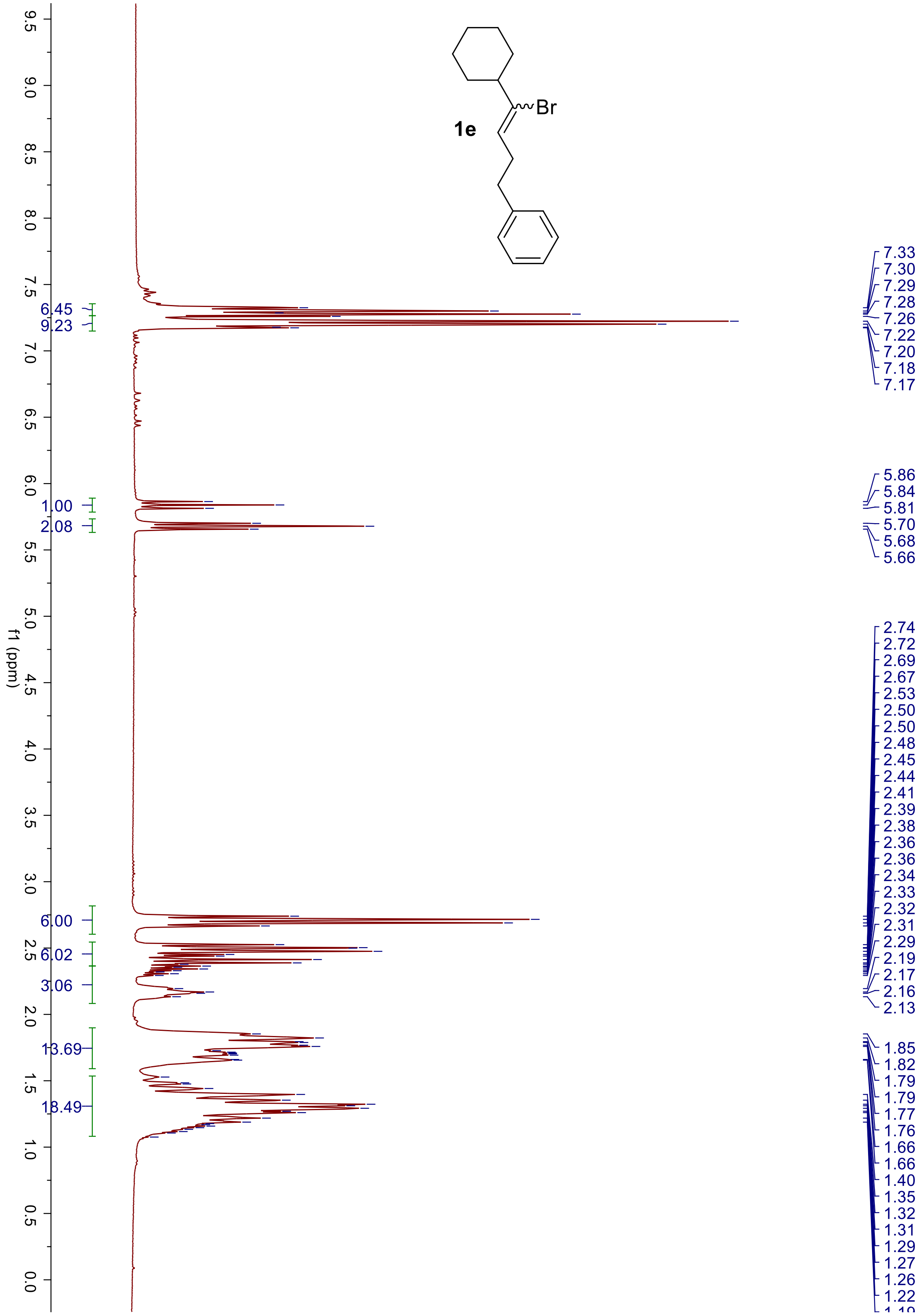

Br
1e
f1 (ppm)
9.5
9.0
8.5
8.0
7.5
7.0
6.5
6.0
5.5
5.0
4.5
4.0
3.5
3.0
2.5
2.0
1.5
1.0
0.5
0.0
6.45
9.23
1.00
2.08
6.00
6.02
3.06
13.69
18.49
7.33
7.30
7.29
7.28
7.26
7.22
7.20
7.18
7.17
5.86
5.84
5.81
5.70
5.68
5.66
2.74
2.72
2.69
2.67
2.53
2.50
2.50
2.48
2.45
2.44
2.41
2.39
2.38
2.36
2.36
2.34
2.33
2.32
2.31
2.29
2.19
2.17
2.16
2.13
1.85
1.82
1.79
1.79
1.77
1.76
1.66
1.66
1.40
1.35
1.32
1.31
1.29
1.27
1.26
1.22

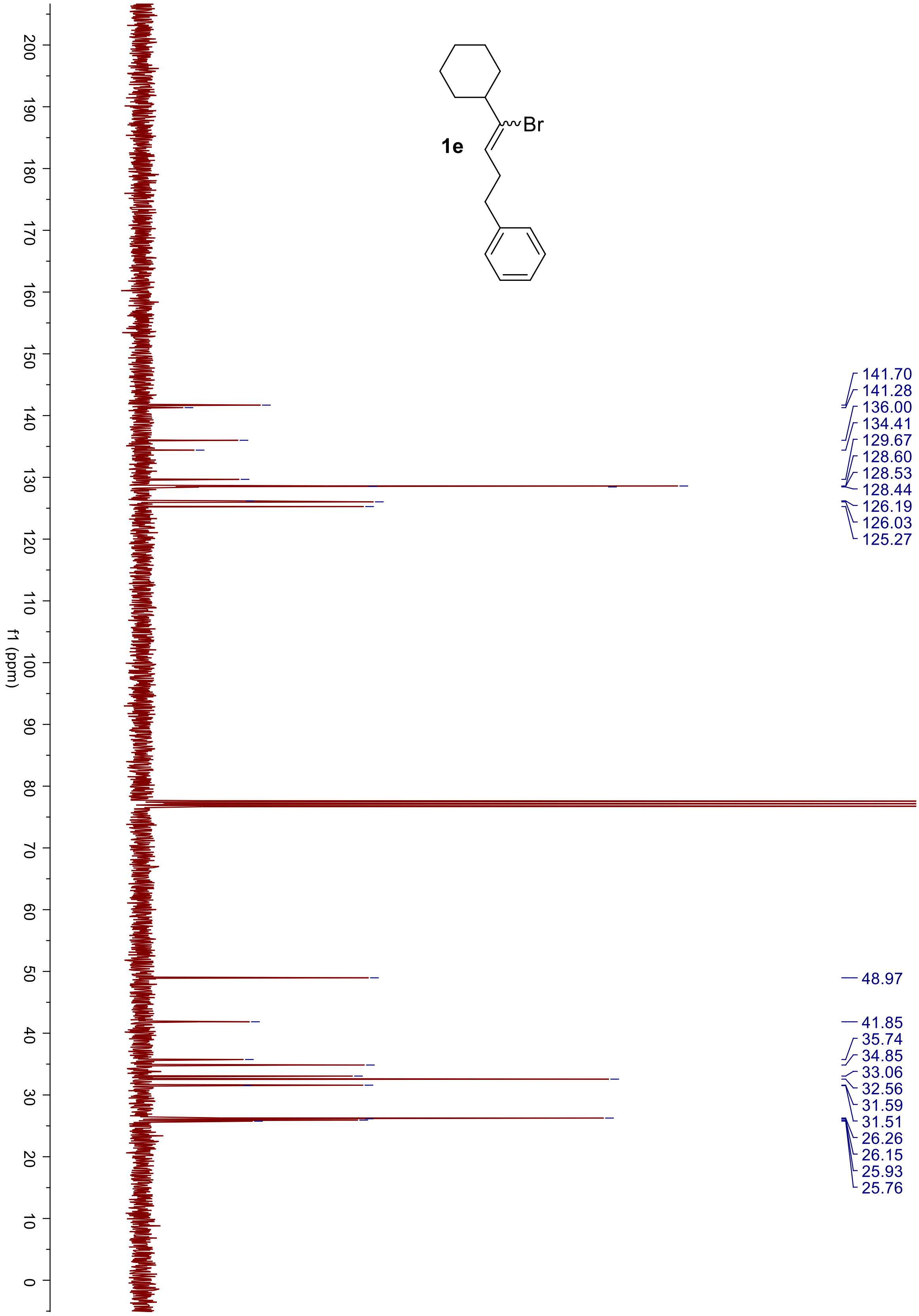

1e
Br
f1 (ppm)
141.70
141.28
136.00
134.41
129.67
128.60
128.53
128.44
126.19
126.03
125.27
48.97
41.85
35.74
34.85
33.06
32.56
31.59
31.51
26.26
26.15
25.93
25.76

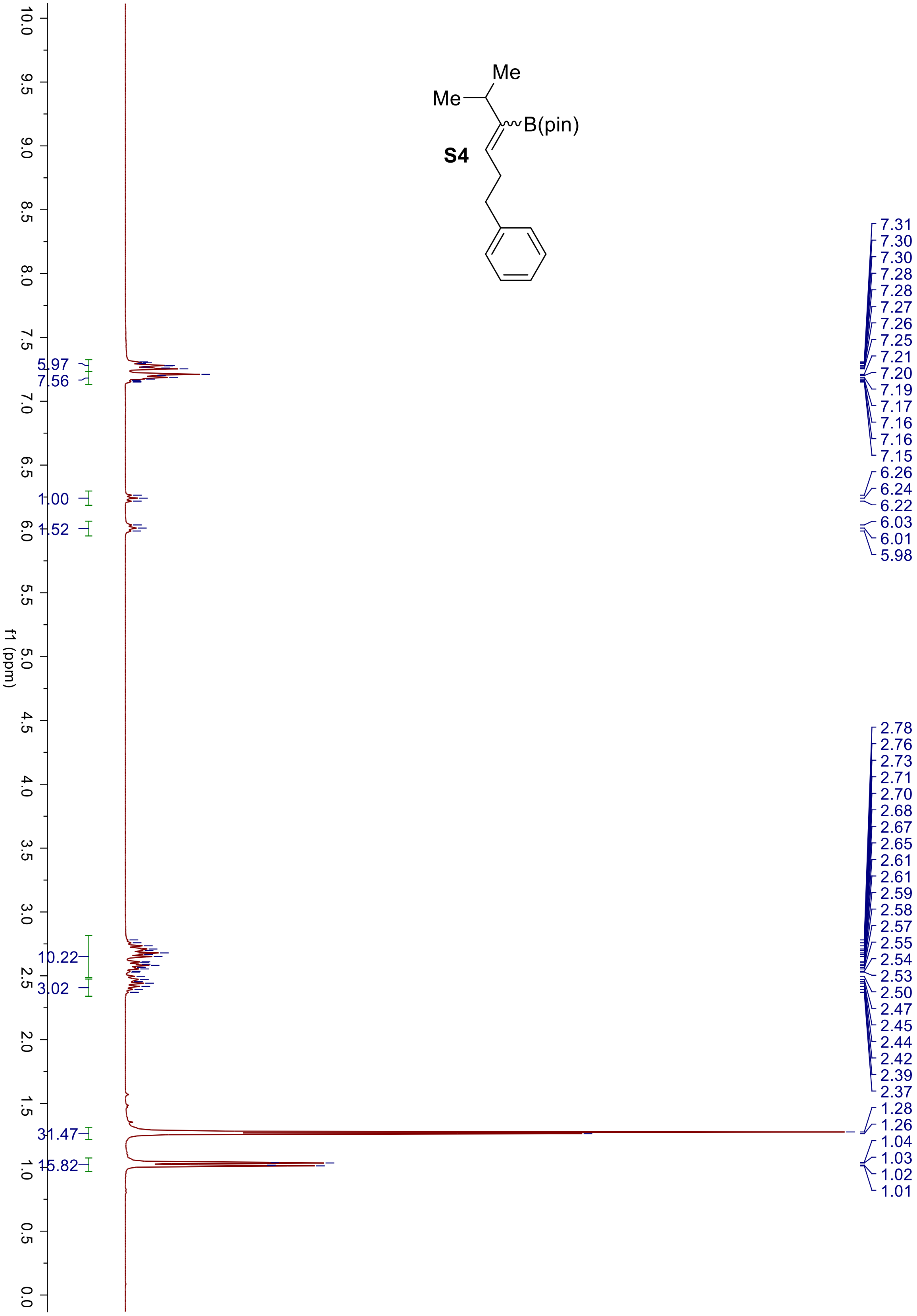
Me
Me
B(pin)
S4
7.31
7.30
7.30
7.28
7.28
7.27
7.26
7.25
7.21
7.20
7.19
7.17
7.16
7.16
7.15
6.26
6.24
6.22
6.03
6.01
5.98
2.78
2.76
2.73
2.71
2.70
2.68
2.67
2.65
2.61
2.61
2.59
2.58
2.57
2.55
2.54
2.53
2.50
2.47
2.45
2.44
2.42
2.39
2.37
1.28
1.26
1.04
1.03
1.02
1.01
5.97
7.56
1.00
1.52
10.22
3.02
31.47
15.82
f1 (ppm)
10.0
9.5
9.0
8.5
8.0
7.5
7.0
6.5
6.0
5.5
5.0
4.5
4.0
3.5
3.0
2.5
2.0
1.5
1.0
0.5
0.0

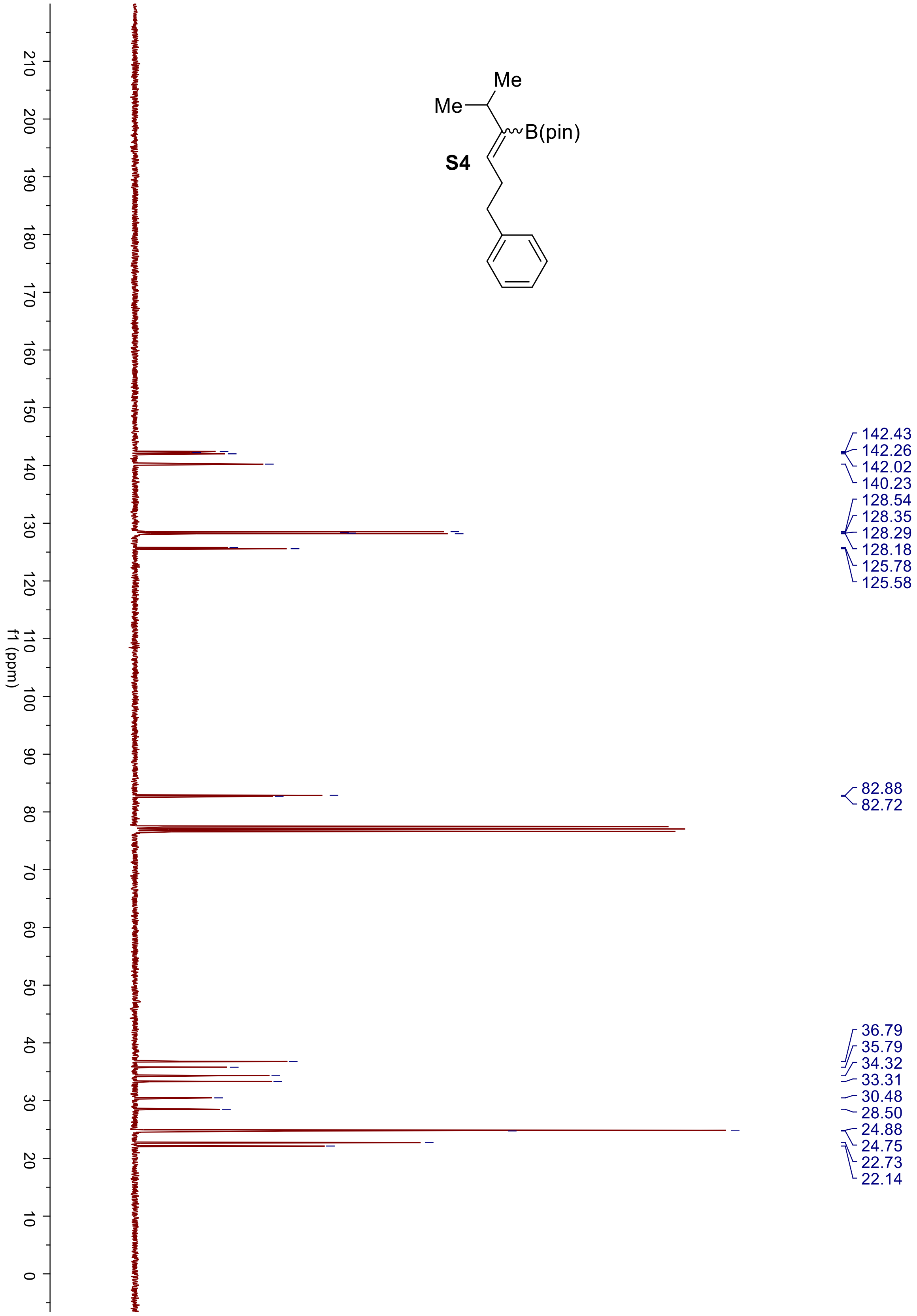
Me
Me
B(pin)
S4
f1 (ppm)
210
200
190
180
170
160
150
140
130
120
110
100
90
80
70
60
50
40
30
20
10
0
142.43
142.26
142.02
140.23
128.54
128.35
128.29
128.18
125.78
125.58
82.88
82.72
36.79
35.79
34.32
33.31
30.48
28.50
24.88
24.75
22.73
22.14

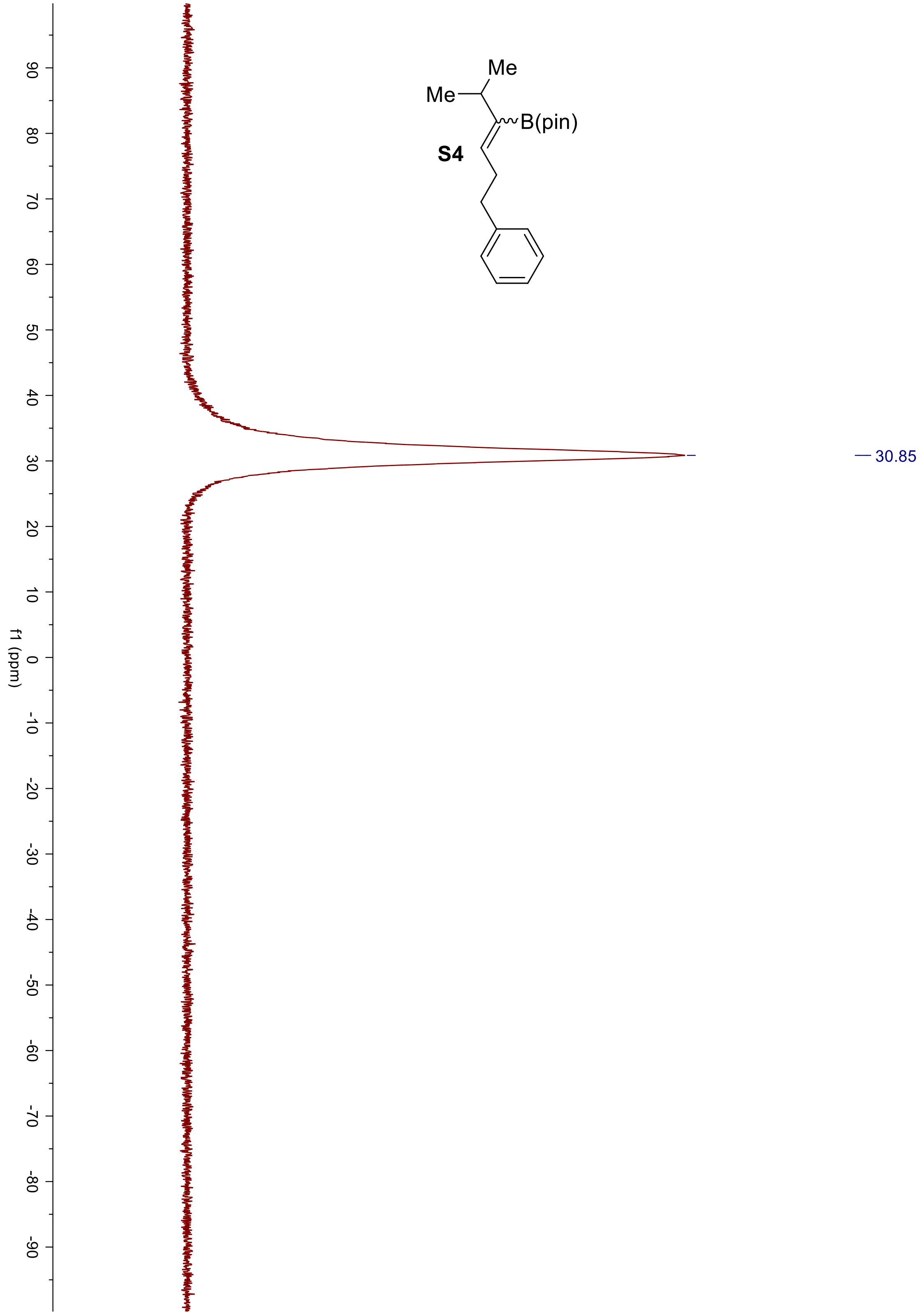
Me
Me
B(pin)
S4
30.85
f1 (ppm)
90
80
70
60
50
40
30
20
10
0
-10
-20
-30
-40
-50
-60
-70
-80
-90

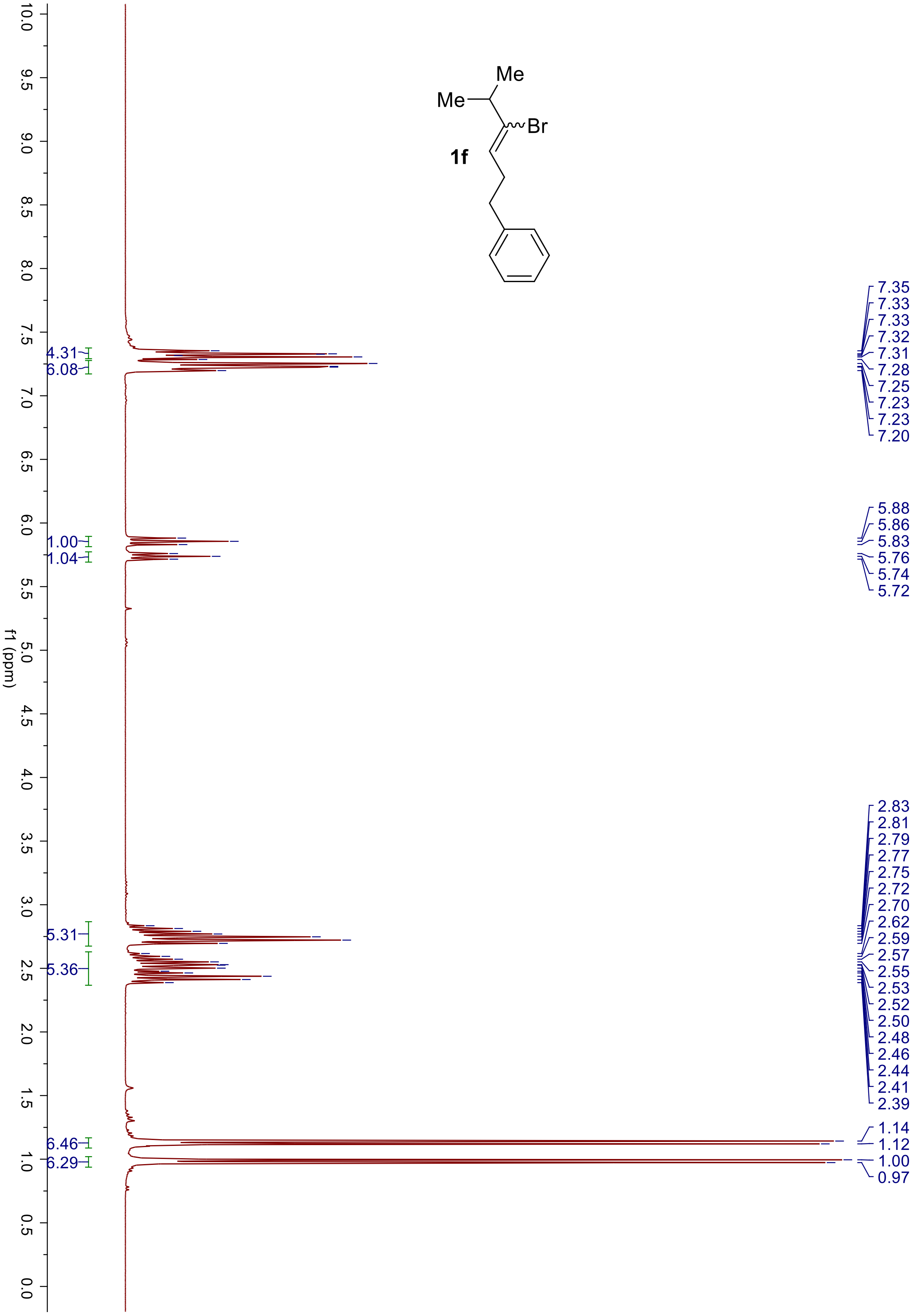
Me
Me
Br
1f
7.35
7.33
7.33
7.32
7.31
7.28
7.25
7.23
7.23
7.20
5.88
5.86
5.83
5.76
5.74
5.72
2.83
2.81
2.79
2.77
2.75
2.72
2.70
2.62
2.59
2.57
2.55
2.53
2.52
2.50
2.48
2.46
2.44
2.41
2.39
1.14
1.12
1.00
0.97
4.31
6.08
1.00
1.04
5.31
5.36
6.46
6.29
f1 (ppm)

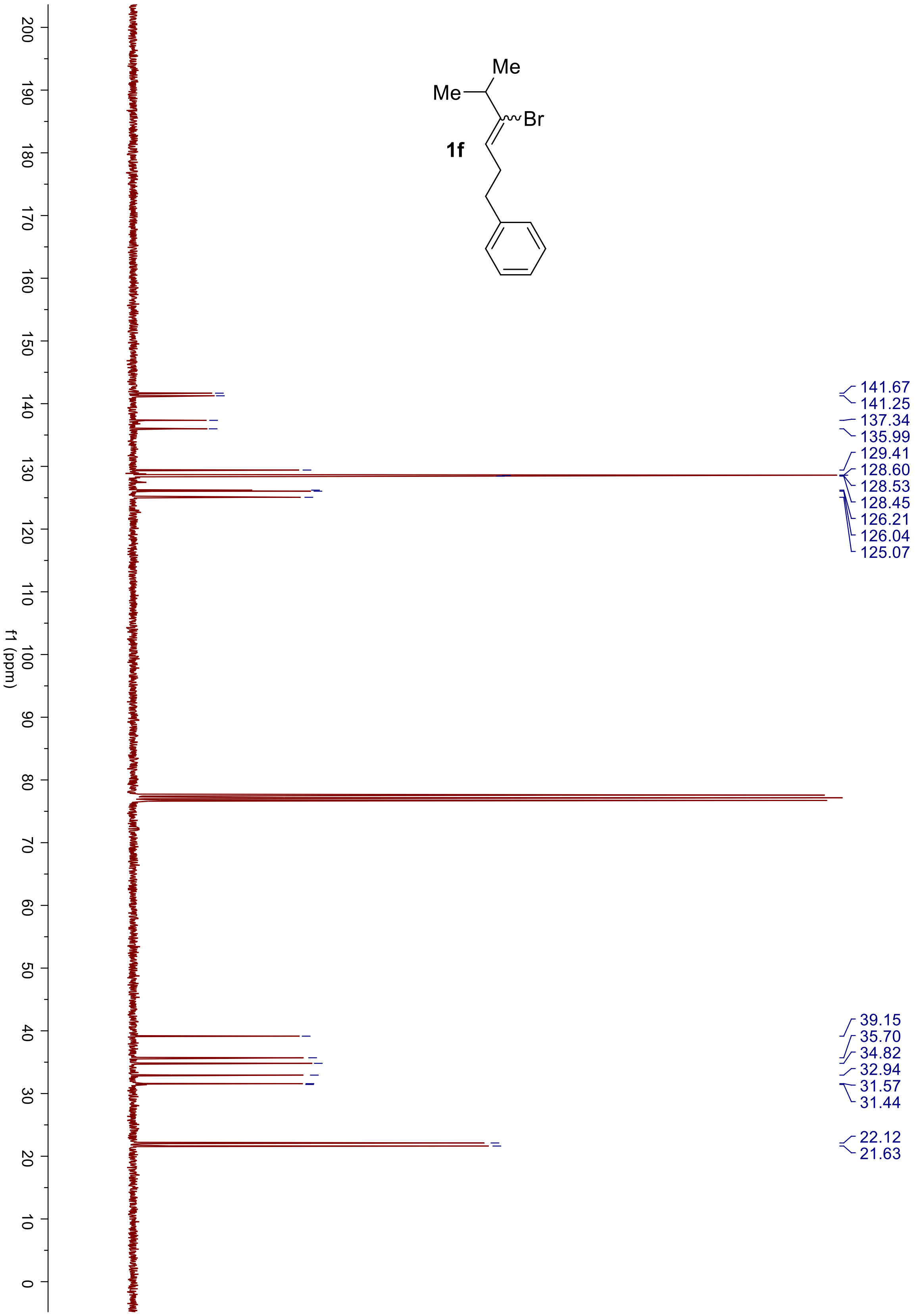
Me
Me
Br
1f
141.67
141.25
137.34
135.99
129.41
128.60
128.53
128.45
126.21
126.04
125.07
39.15
35.70
34.82
32.94
31.57
31.44
22.12
21.63
f1 (ppm)

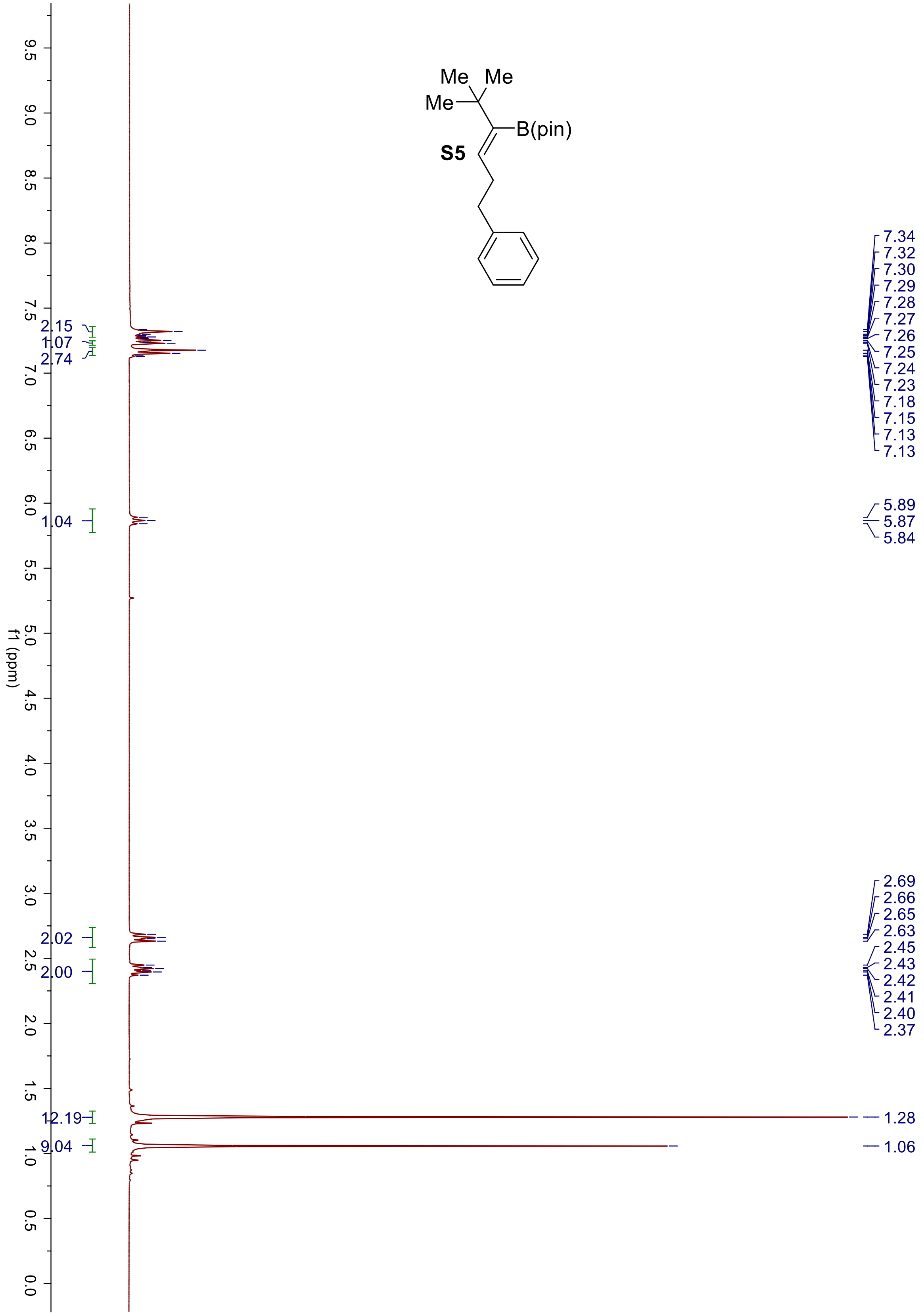
Me Me
Me
B(pin)
S5
7.34
7.32
7.30
7.29
7.28
7.27
7.26
7.25
7.24
7.23
7.18
7.15
7.13
7.13
5.89
5.87
5.84
2.69
2.66
2.65
2.63
2.45
2.43
2.42
2.41
2.40
2.37
1.28
1.06
2.15
1.07
2.74
1.04
2.02
2.00
12.19
9.04
f1 (ppm)

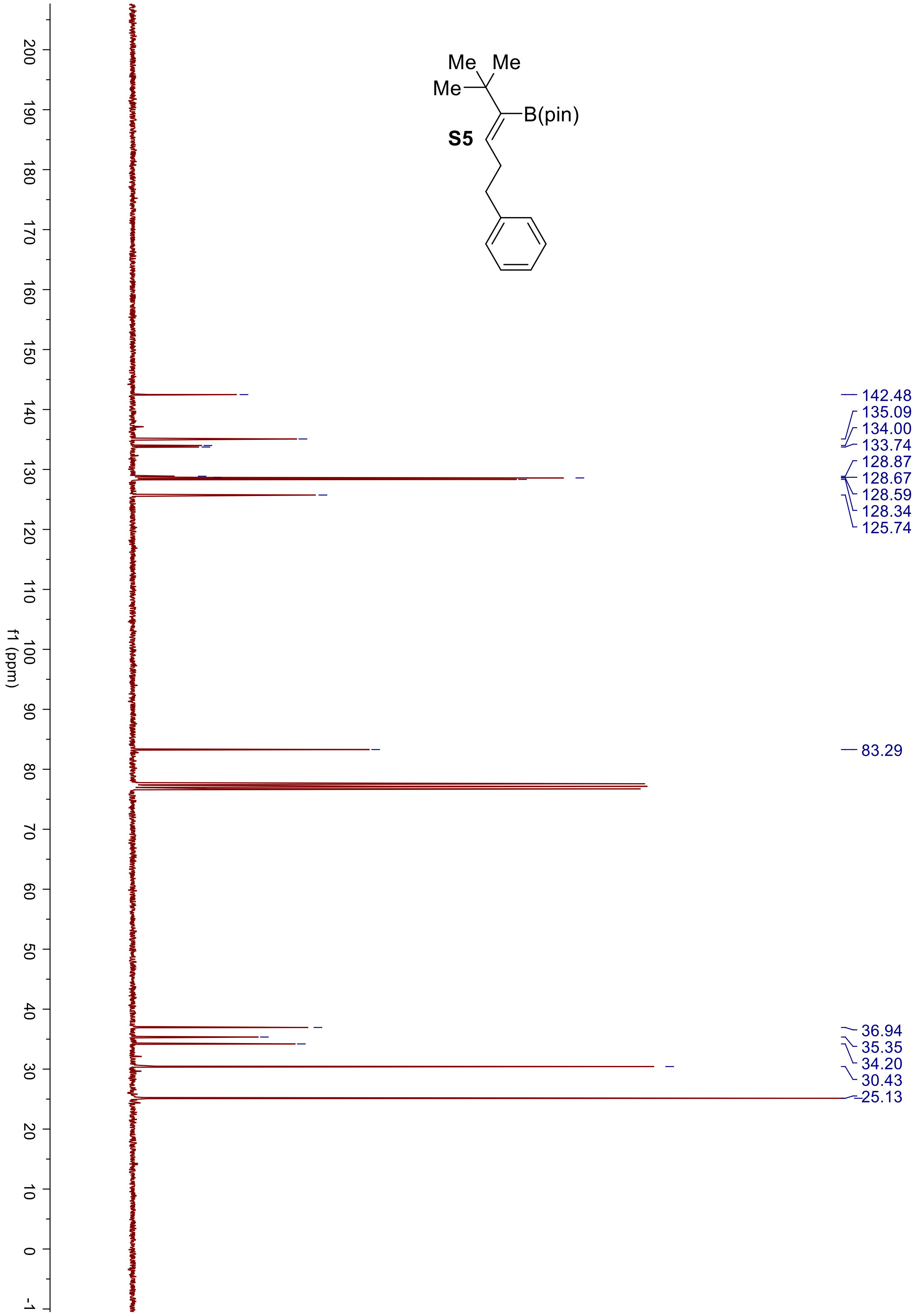
Me
Me
Me
B(pin)
S5
142.48
135.09
134.00
133.74
128.87
128.67
128.59
128.34
125.74
83.29
36.94
35.35
34.20
30.43
25.13
f1 (ppm)
200
190
180
170
160
150
140
130
120
110
100
90
80
70
60
50
40
30
20
10
0
-1

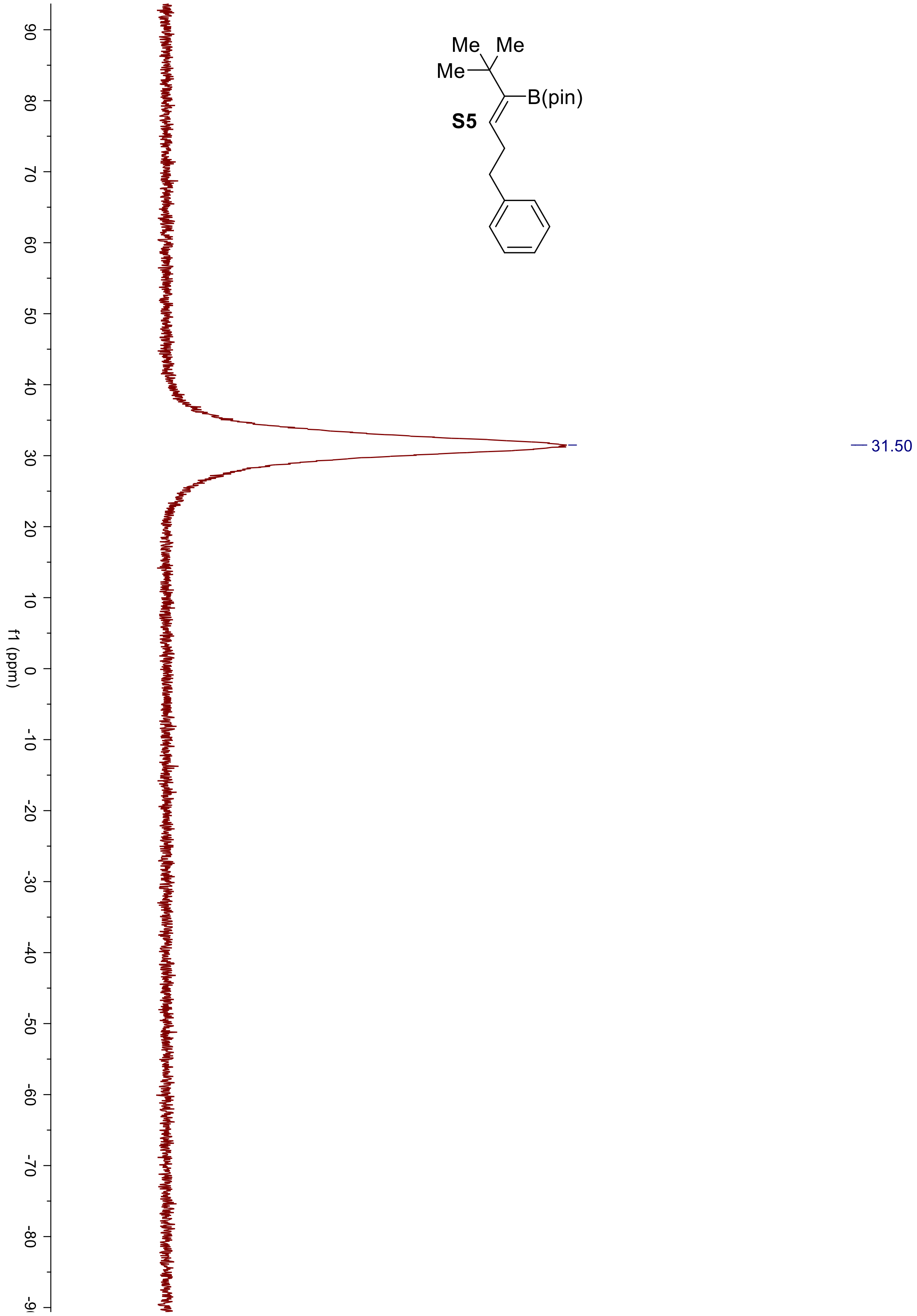
Me
Me
Me
B(pin)
S5
31.50
f1 (ppm)

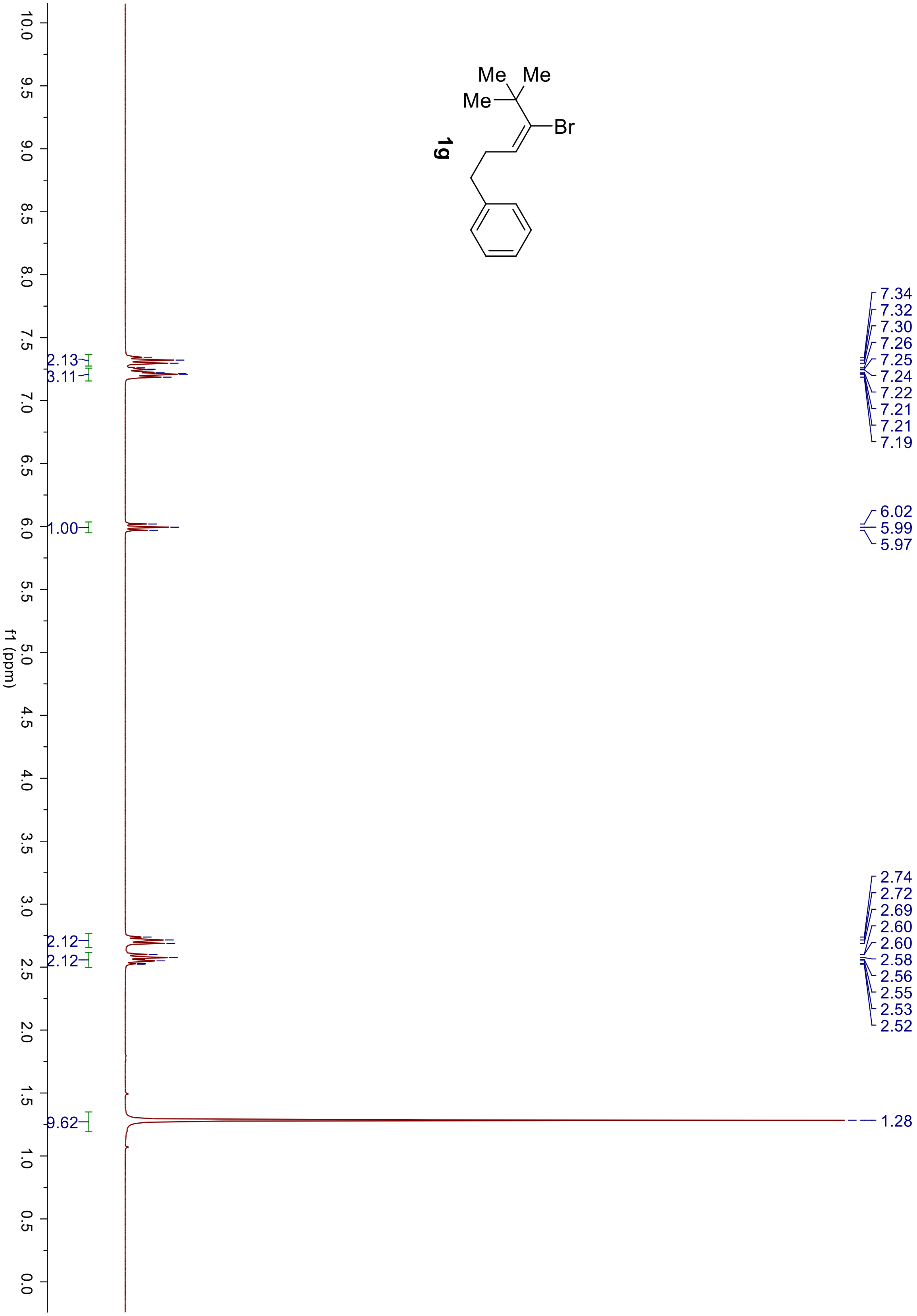

1g
Me
Me
Me
Br
f1 (ppm)
7.34
7.32
7.30
7.26
7.25
7.24
7.22
7.21
7.21
7.19
6.02
5.99
5.97
2.74
2.72
2.69
2.60
2.60
2.58
2.56
2.55
2.53
2.52
1.28
2.13
3.11
1.00
2.12
2.12
9.62

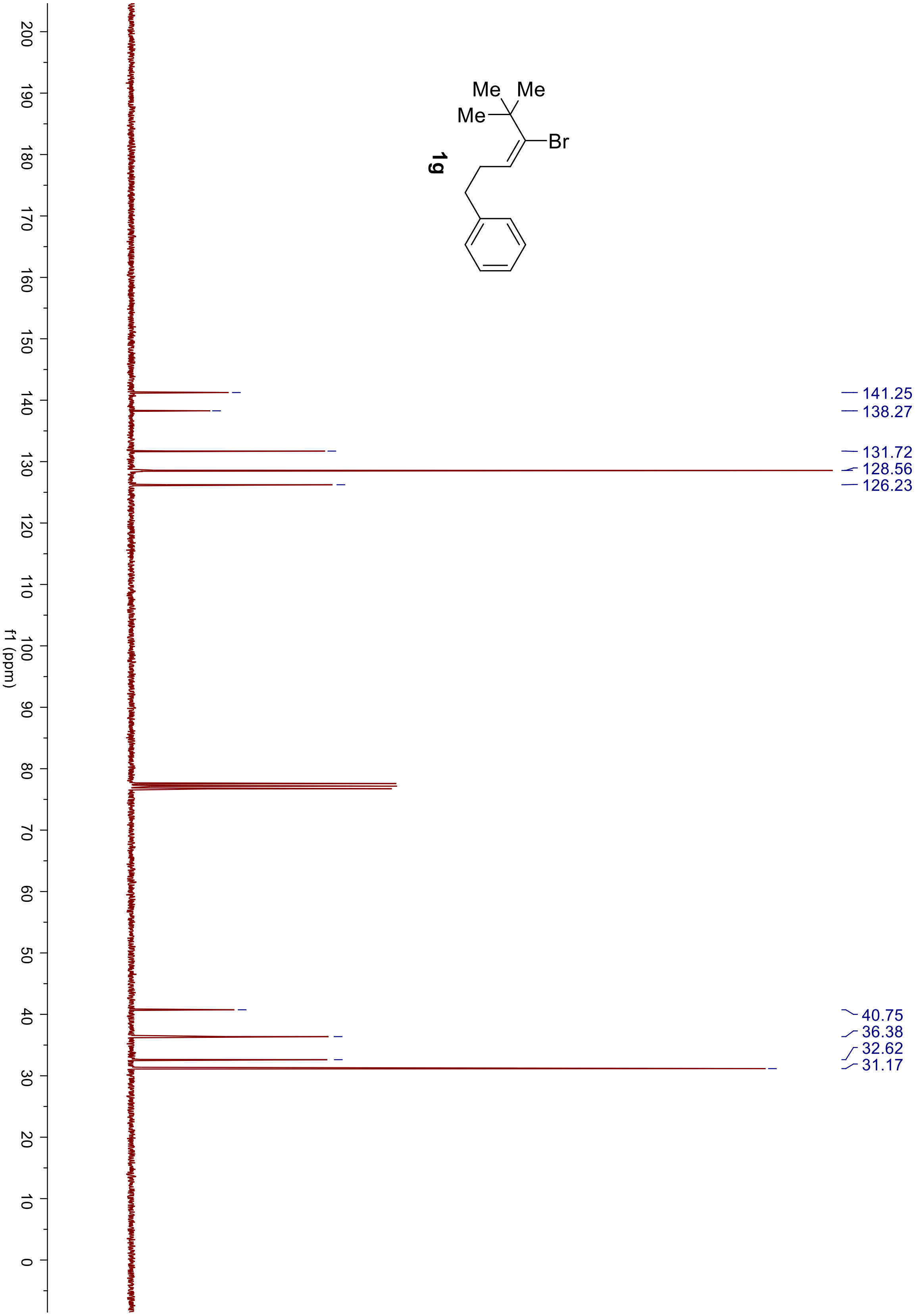

Me Me
Me
Br
1g
141.25
138.27
131.72
128.56
126.23
40.75
36.38
32.62
31.17
f1 (ppm)
200
190
180
170
160
150
140
130
120
110
100
90
80
70
60
50
40
30
20
10
0

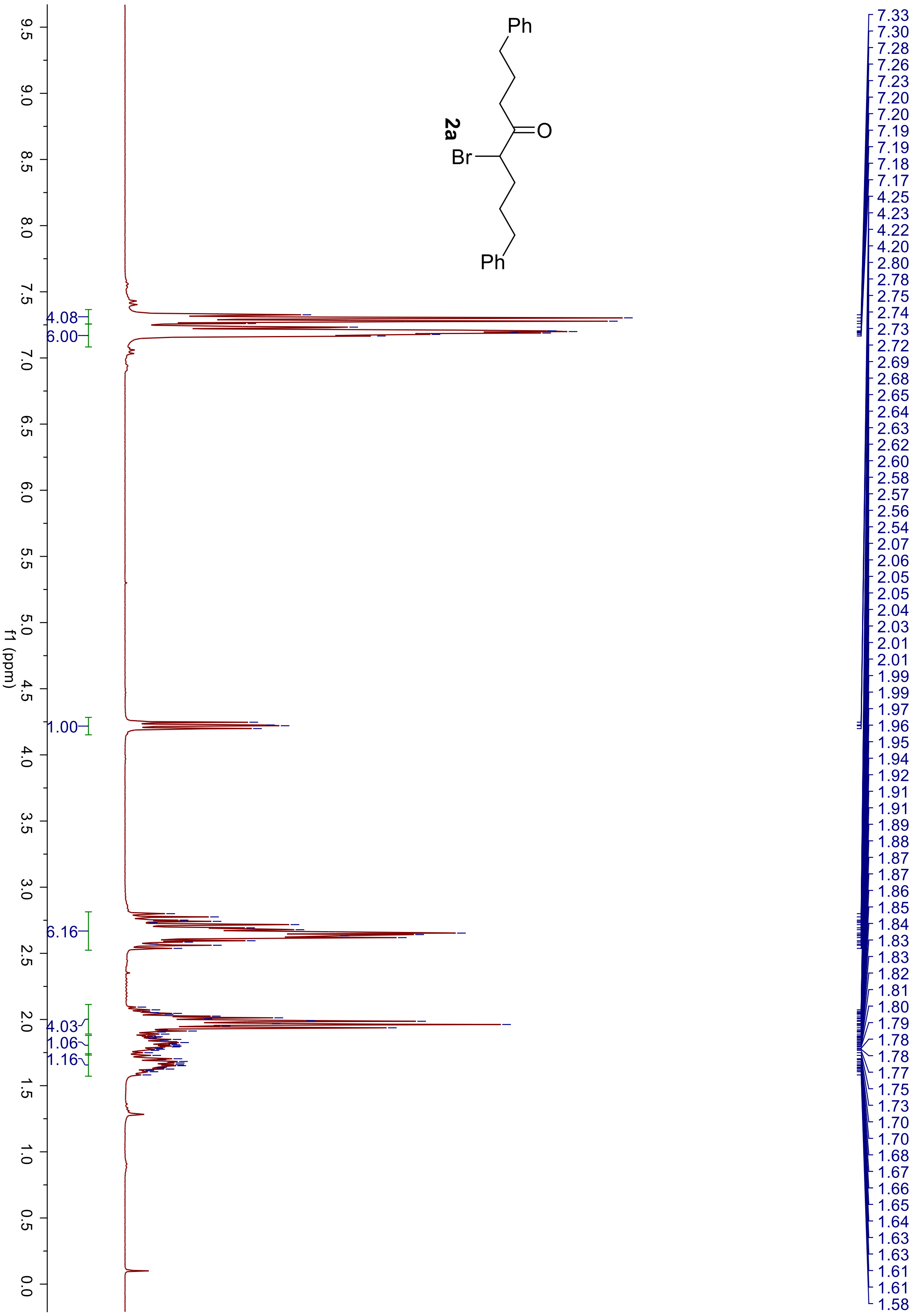
Ph
Ph
Br
O
2a
f1 (ppm)
9.5
9.0
8.5
8.0
7.5
7.0
6.5
6.0
5.5
5.0
4.5
4.0
3.5
3.0
2.5
2.0
1.5
1.0
0.5
0.0
4.08
6.00
1.00
6.16
4.03
1.06
1.16
7.33
7.30
7.28
7.26
7.23
7.20
7.20
7.19
7.19
7.18
7.17
4.25
4.23
4.22
4.20
2.80
2.78
2.75
2.74
2.73
2.72
2.69
2.68
2.65
2.64
2.63
2.62
2.60
2.58
2.57
2.56
2.54
2.07
2.06
2.05
2.05
2.04
2.03
2.01
2.01
1.99
1.99
1.97
1.96
1.95
1.94
1.92
1.91
1.91
1.89
1.88
1.87
1.87
1.86
1.85
1.84
1.83
1.83
1.82
1.81
1.80
1.79
1.78
1.78
1.77
1.75
1.73
1.70
1.70
1.68
1.67
1.66
1.65
1.64
1.63
1.63
1.61
1.61
1.58

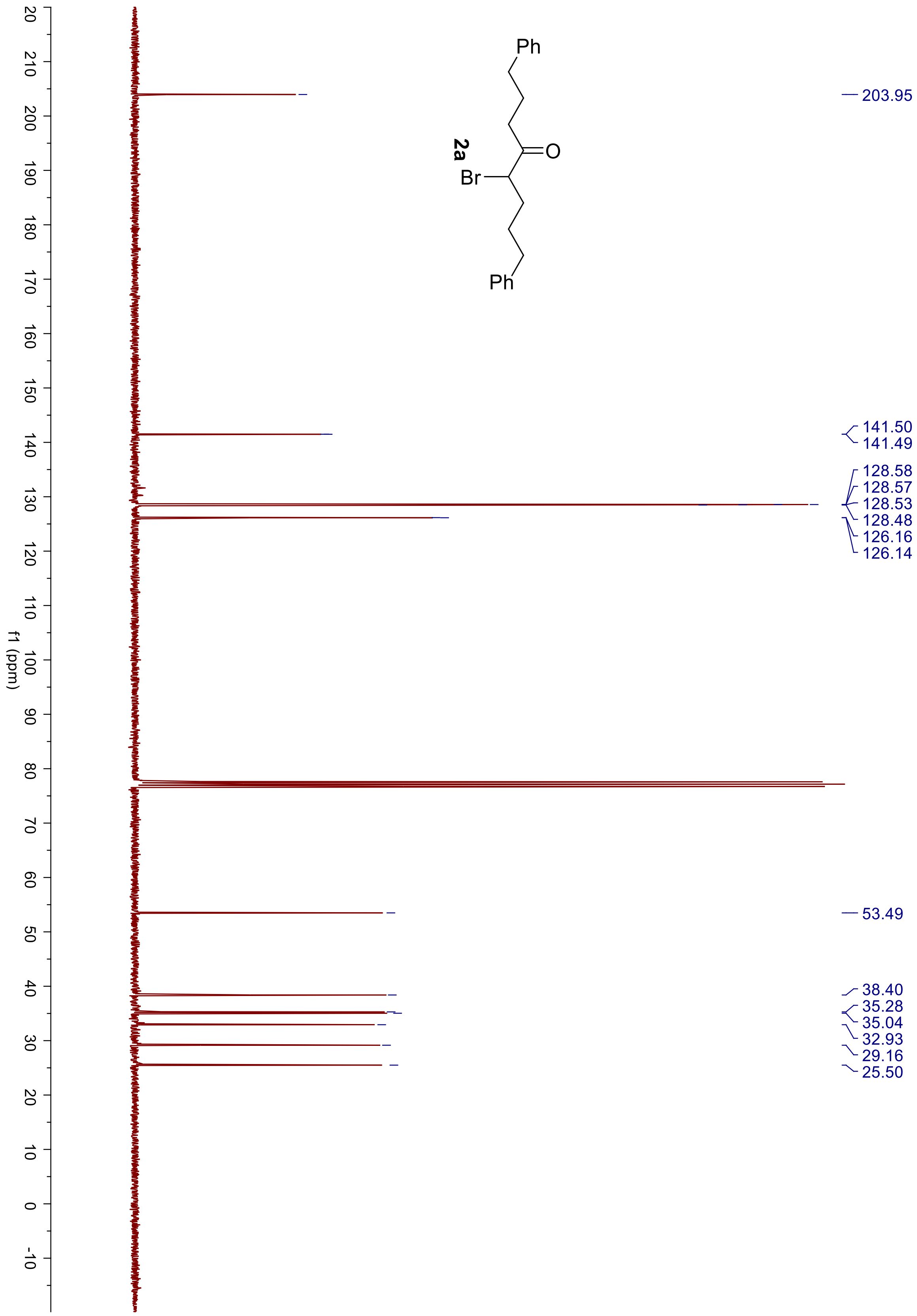
Ph
O
2a
Br
Ph
203.95
141.50
141.49
128.58
128.57
128.53
128.48
126.16
126.14
53.49
38.40
35.28
35.04
32.93
29.16
25.50
f1 (ppm)

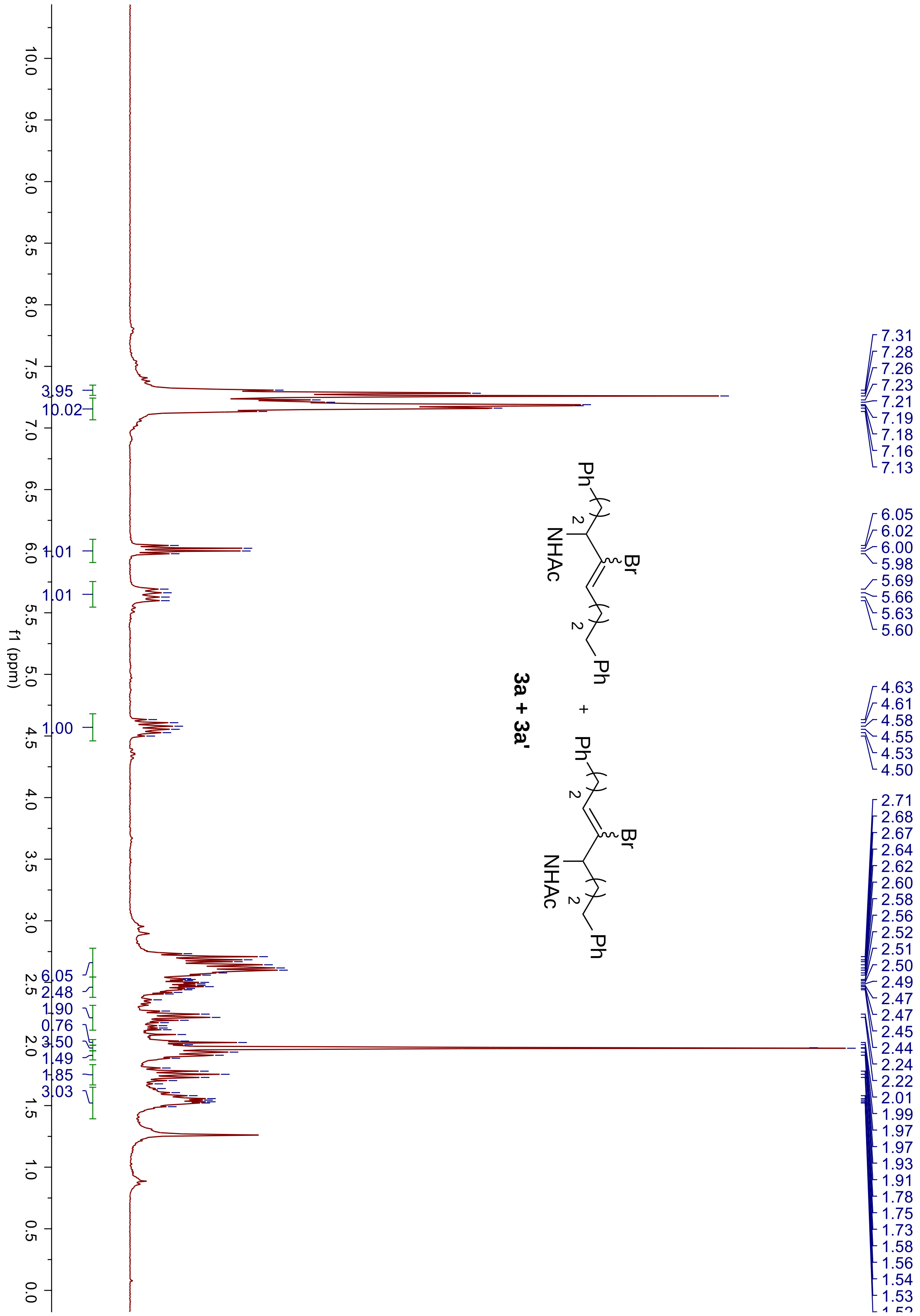

f1 (ppm)
3a + 3a'
Br
NHAc
Ph

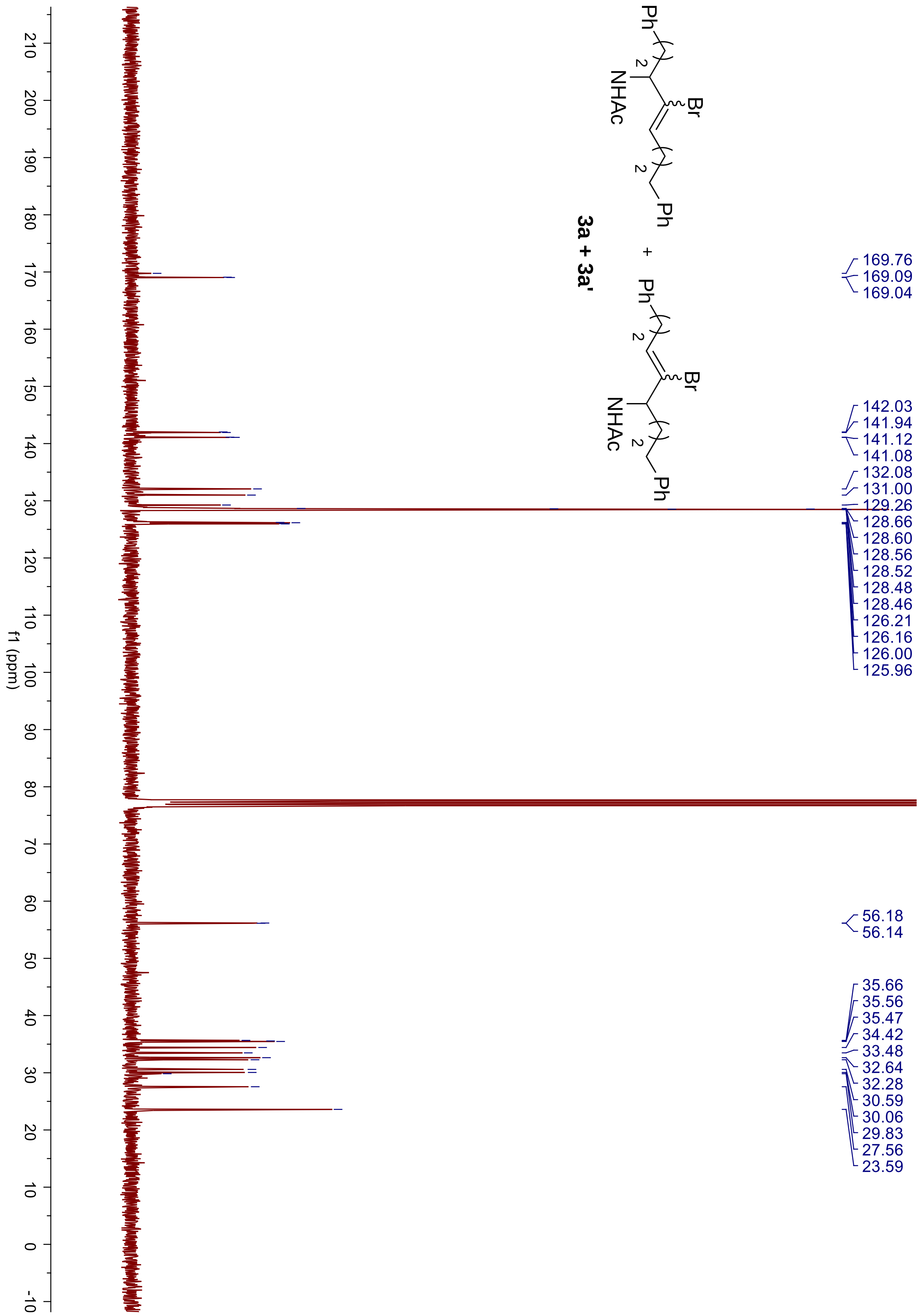
3a + 3a'
Ph
Br
NHAc
Ph
169.76
169.09
169.04
142.03
141.94
141.12
141.08
132.08
131.00
129.26
128.66
128.60
128.56
128.52
128.48
128.46
126.21
126.16
126.00
125.96
56.18
56.14
35.66
35.56
35.47
34.42
33.48
32.64
32.28
30.59
30.06
29.83
27.56
23.59
f1 (ppm)

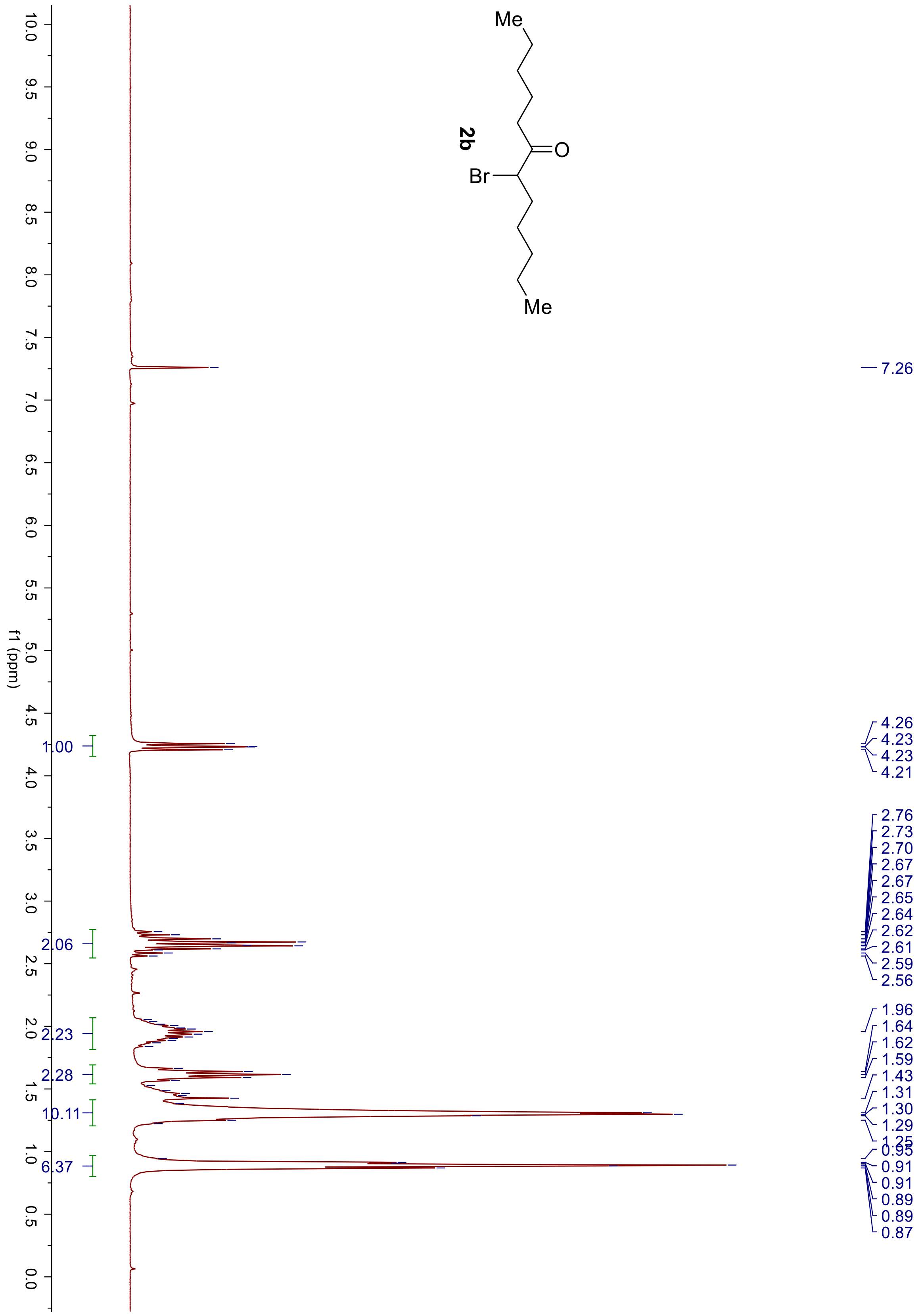
2b
Me
O
Br
Me
f1 (ppm)
1.00
2.06
2.23
2.28
10.11
6.37
7.26
4.26
4.23
4.23
4.21
2.76
2.73
2.70
2.67
2.67
2.65
2.64
2.62
2.61
2.59
2.56
1.96
1.64
1.62
1.59
1.43
1.31
1.30
1.29
1.25
0.95
0.91
0.91
0.89
0.89
0.87

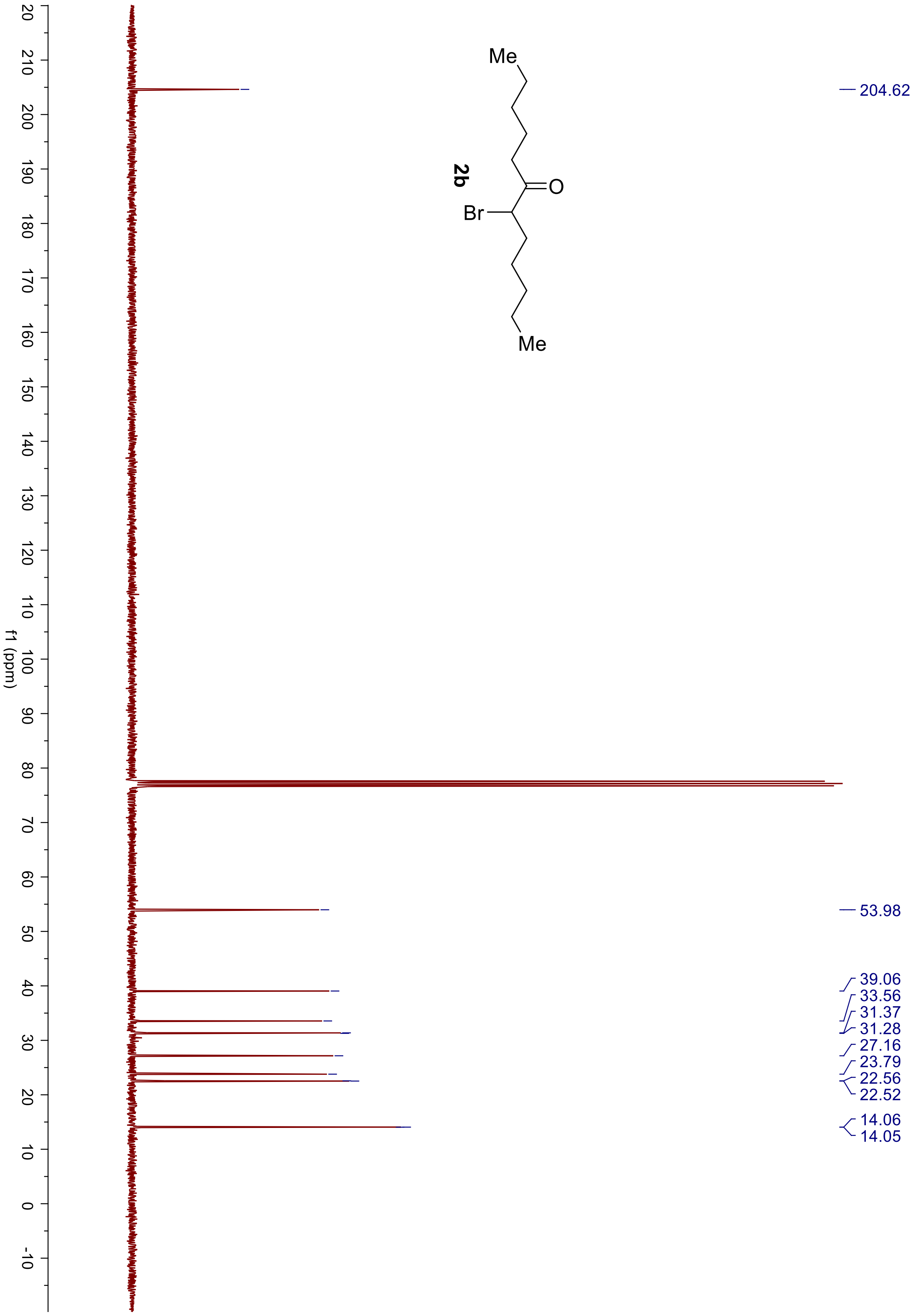

2b
Me
Br
O
Me
204.62
53.98
39.06
33.56
31.37
31.28
27.16
23.79
22.56
22.52
14.06
14.05
f1 (ppm)

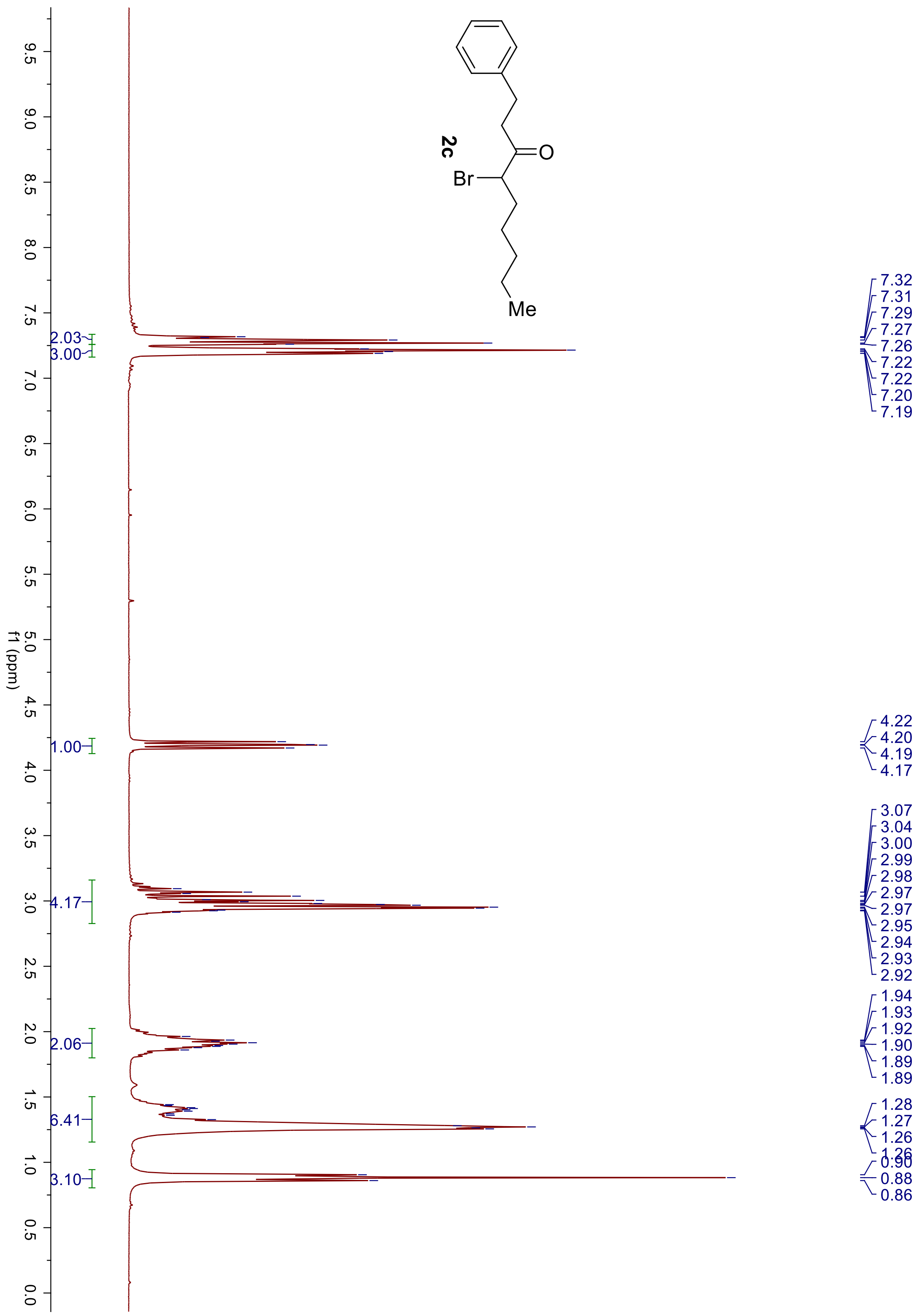
2c
Br
O
Me
f1 (ppm)
2.03
3.00
1.00
4.17
2.06
6.41
3.10
7.32
7.31
7.29
7.27
7.26
7.22
7.22
7.20
7.19
4.22
4.20
4.19
4.17
3.07
3.04
3.00
2.99
2.98
2.97
2.97
2.95
2.94
2.93
2.92
1.94
1.93
1.92
1.90
1.89
1.89
1.28
1.27
1.26
0.90
0.88
0.86

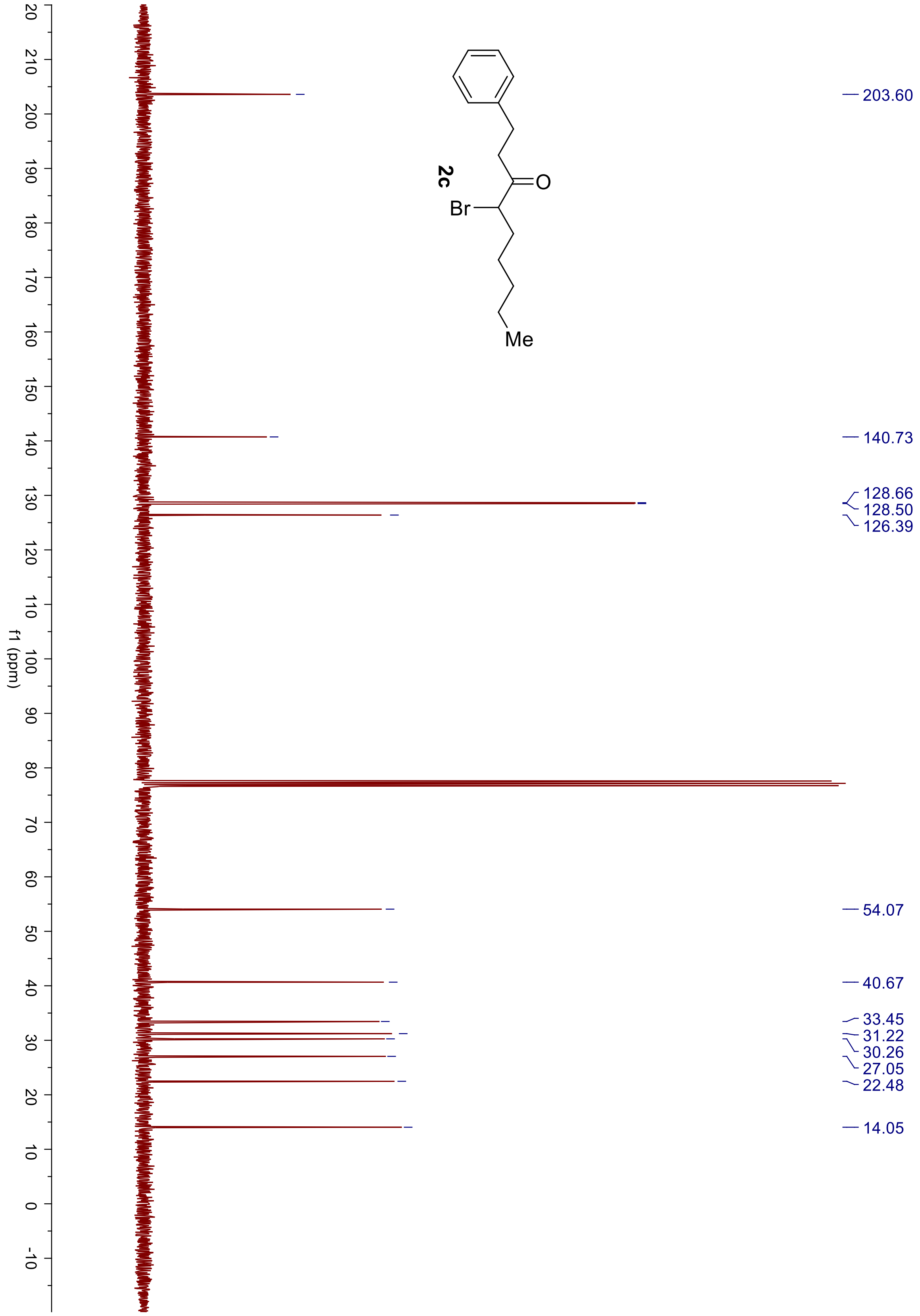
2c
Br
O
Me
203.60
140.73
128.66
128.50
126.39
54.07
40.67
33.45
31.22
30.26
27.05
22.48
14.05
f1 (ppm)

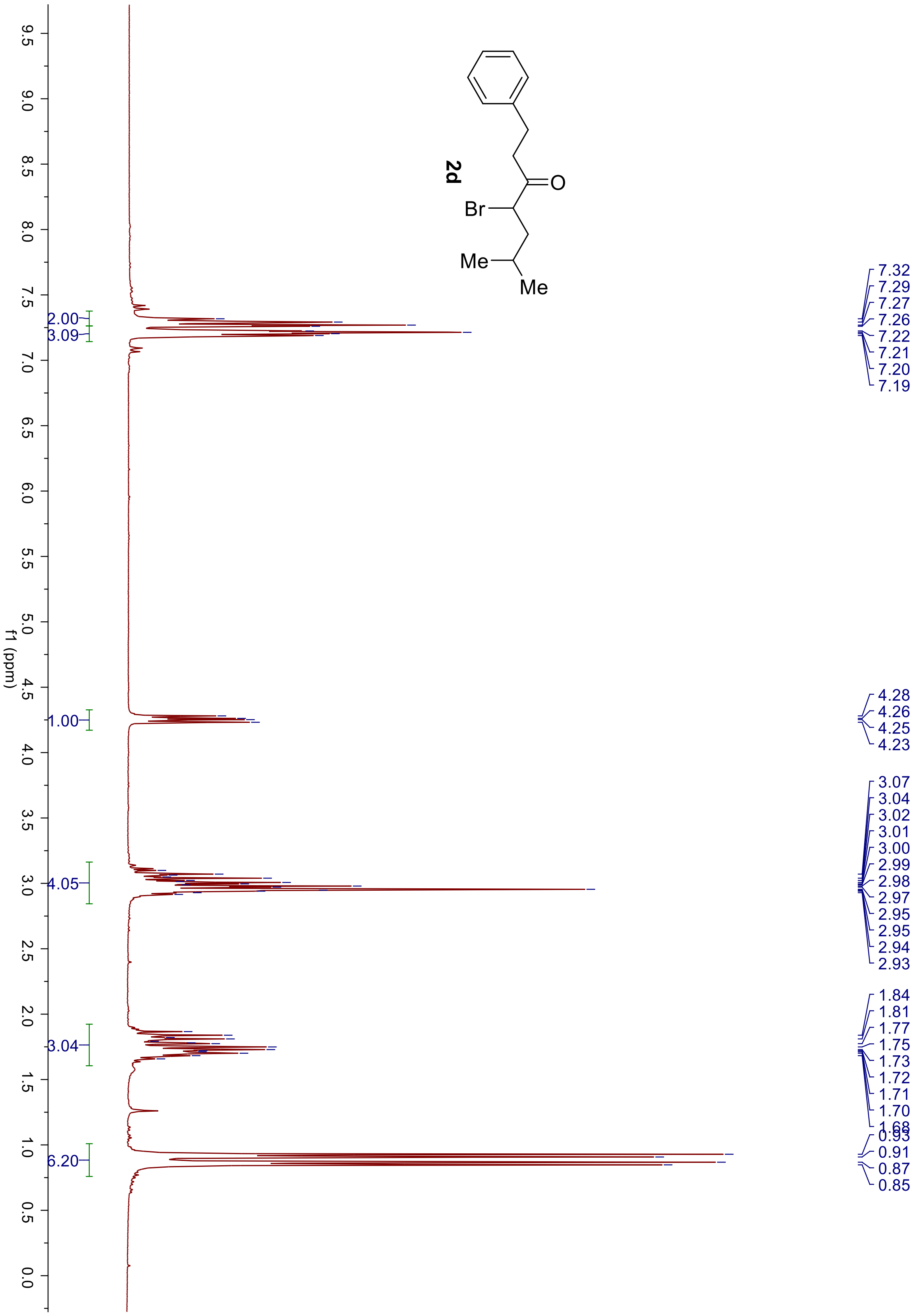
2d
Br
O
Me
Me
f1 (ppm)
2.00
3.09
1.00
4.05
3.04
6.20
7.32
7.29
7.27
7.26
7.22
7.21
7.20
7.19
4.28
4.26
4.25
4.23
3.07
3.04
3.02
3.01
3.00
2.99
2.98
2.97
2.95
2.95
2.94
2.93
1.84
1.81
1.77
1.75
1.73
1.72
1.71
1.70
1.68
0.93
0.91
0.87
0.85

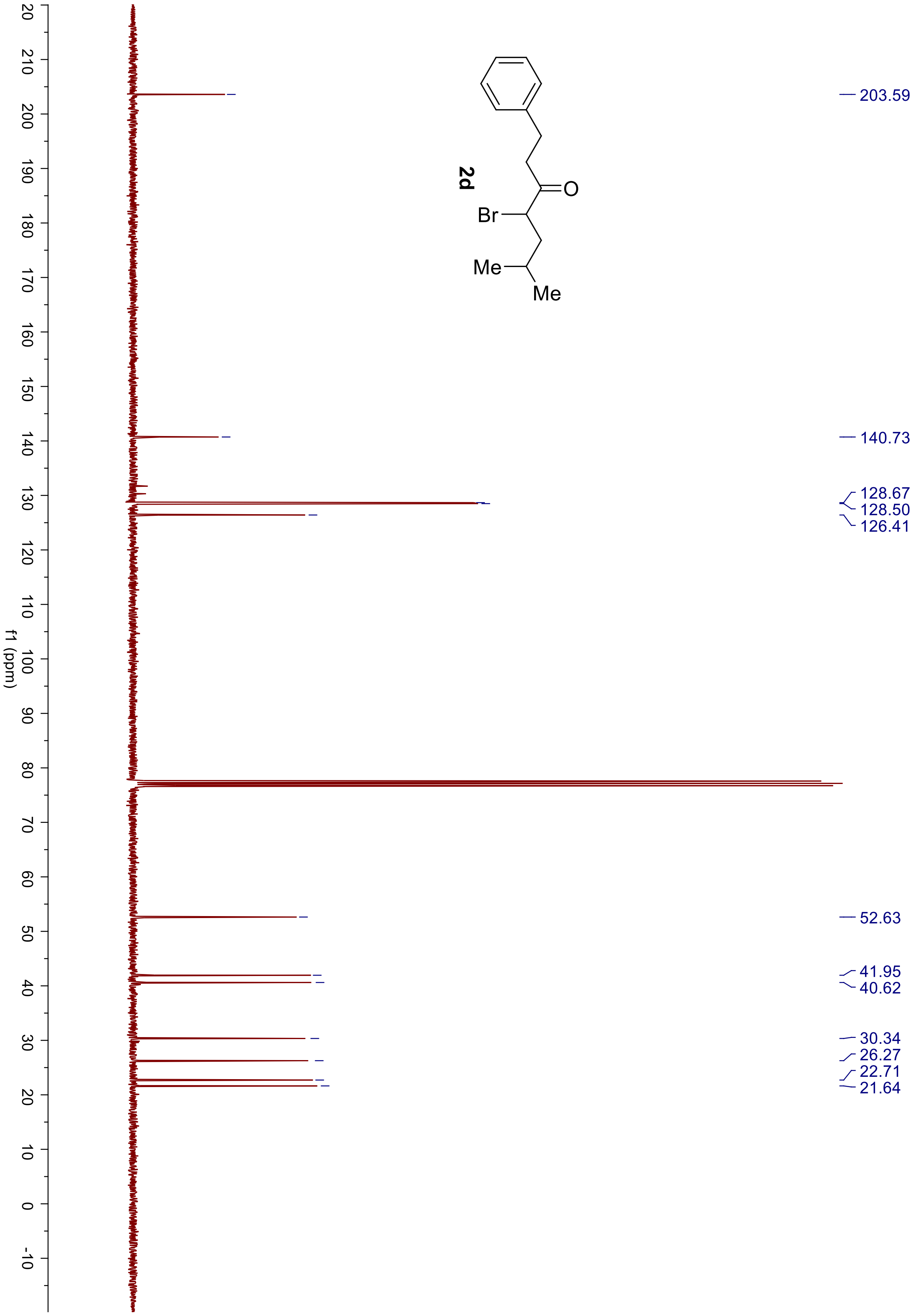
2d
Br
O
Me
Me
203.59
140.73
128.67
128.50
126.41
52.63
41.95
40.62
30.34
26.27
22.71
21.64
f1 (ppm)

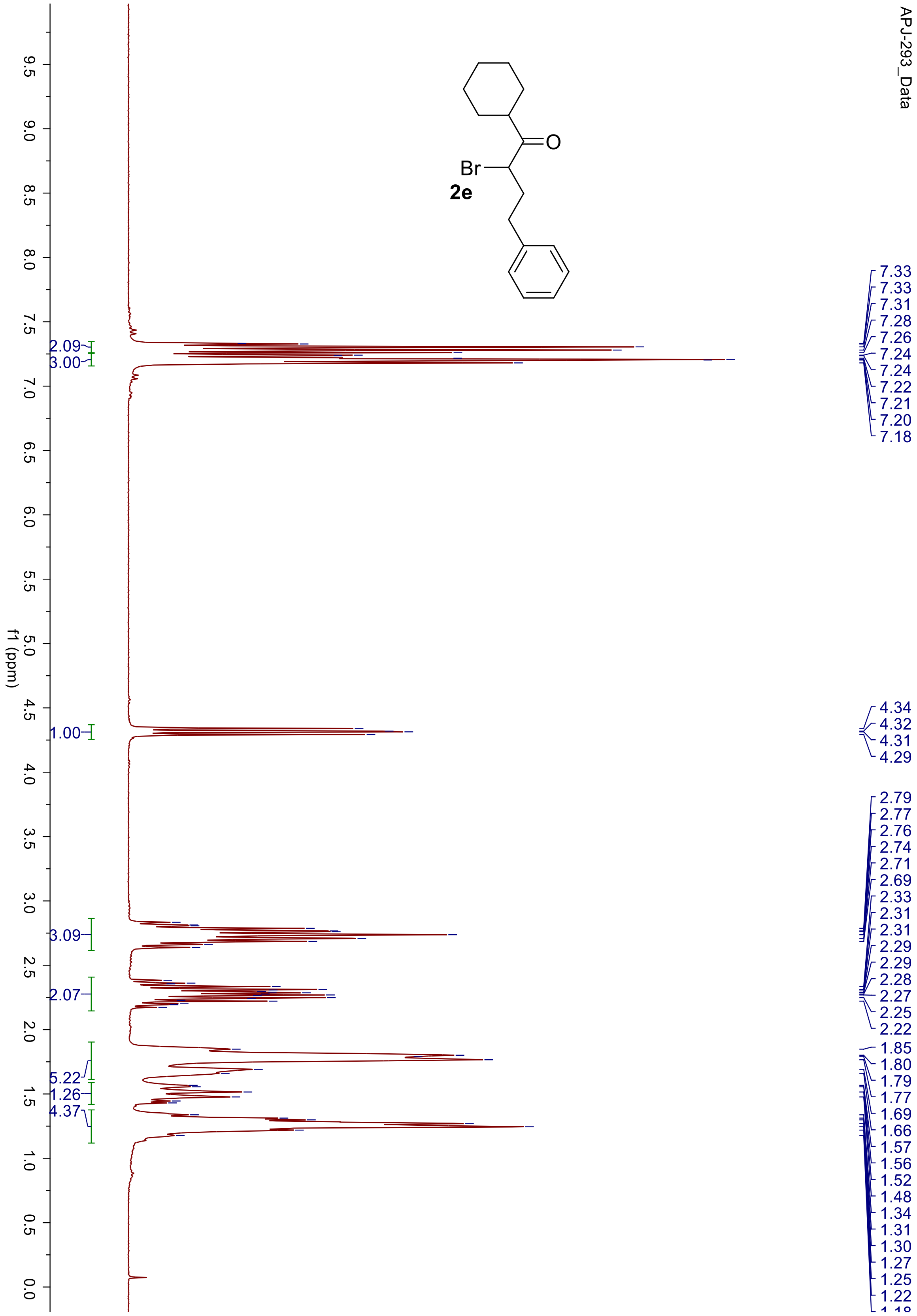
APJ-293_Data
Br
2e
O
7.33
7.33
7.31
7.28
7.26
7.24
7.24
7.22
7.21
7.20
7.18
4.34
4.32
4.31
4.29
2.79
2.77
2.76
2.74
2.71
2.69
2.33
2.31
2.31
2.29
2.29
2.28
2.27
2.25
2.22
1.85
1.80
1.79
1.77
1.69
1.66
1.57
1.56
1.52
1.48
1.34
1.31
1.30
1.27
1.25
1.22
2.09
3.00
1.00
3.09
2.07
5.22
1.26
4.37
f1 (ppm)

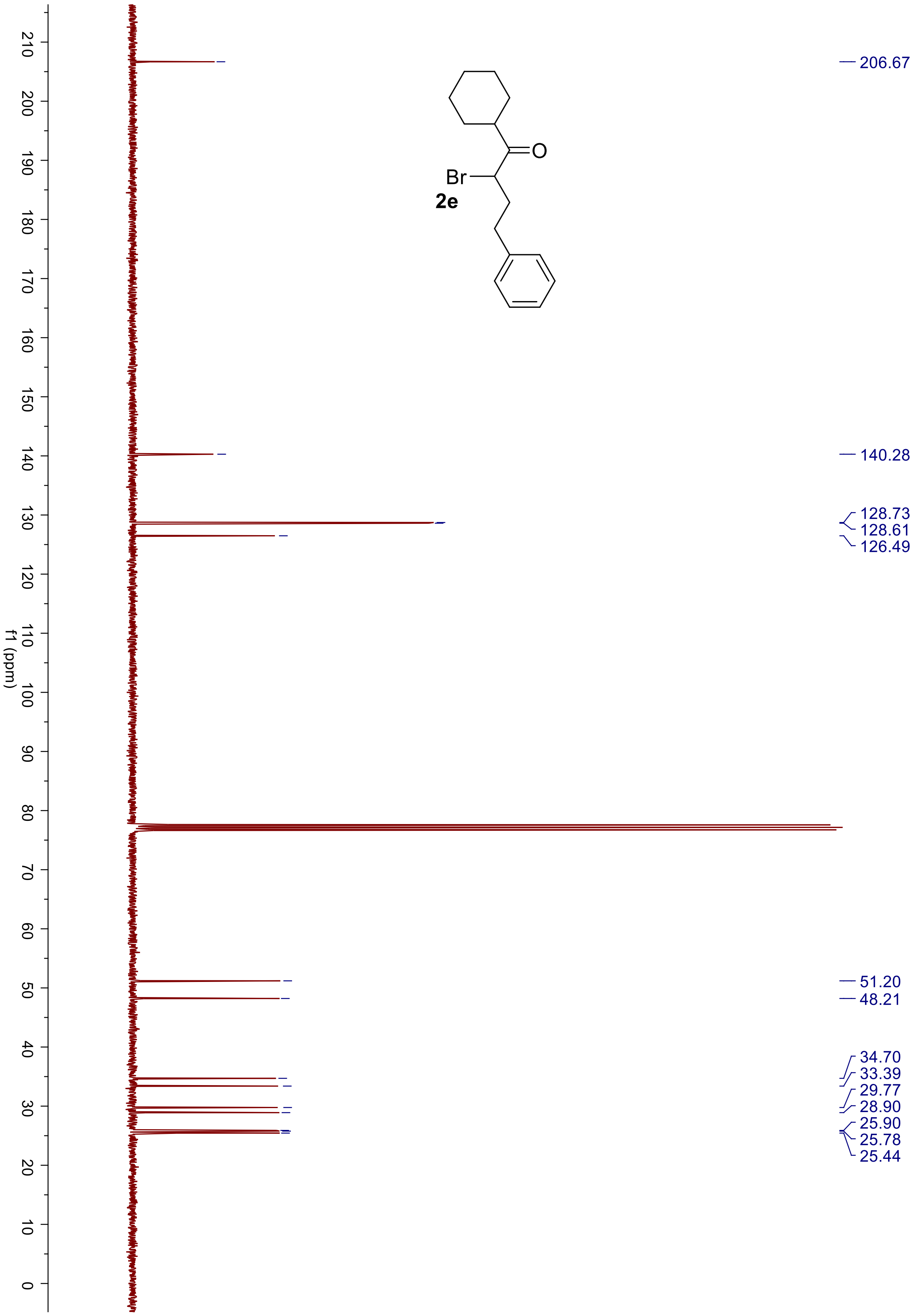

Br
2e
O
206.67
140.28
128.73
128.61
126.49
51.20
48.21
34.70
33.39
29.77
28.90
25.90
25.78
25.44
f1 (ppm)
210 200 190 180 170 160 150 140 130 120 110 100 90 80 70 60 50 40 30 20 10 0

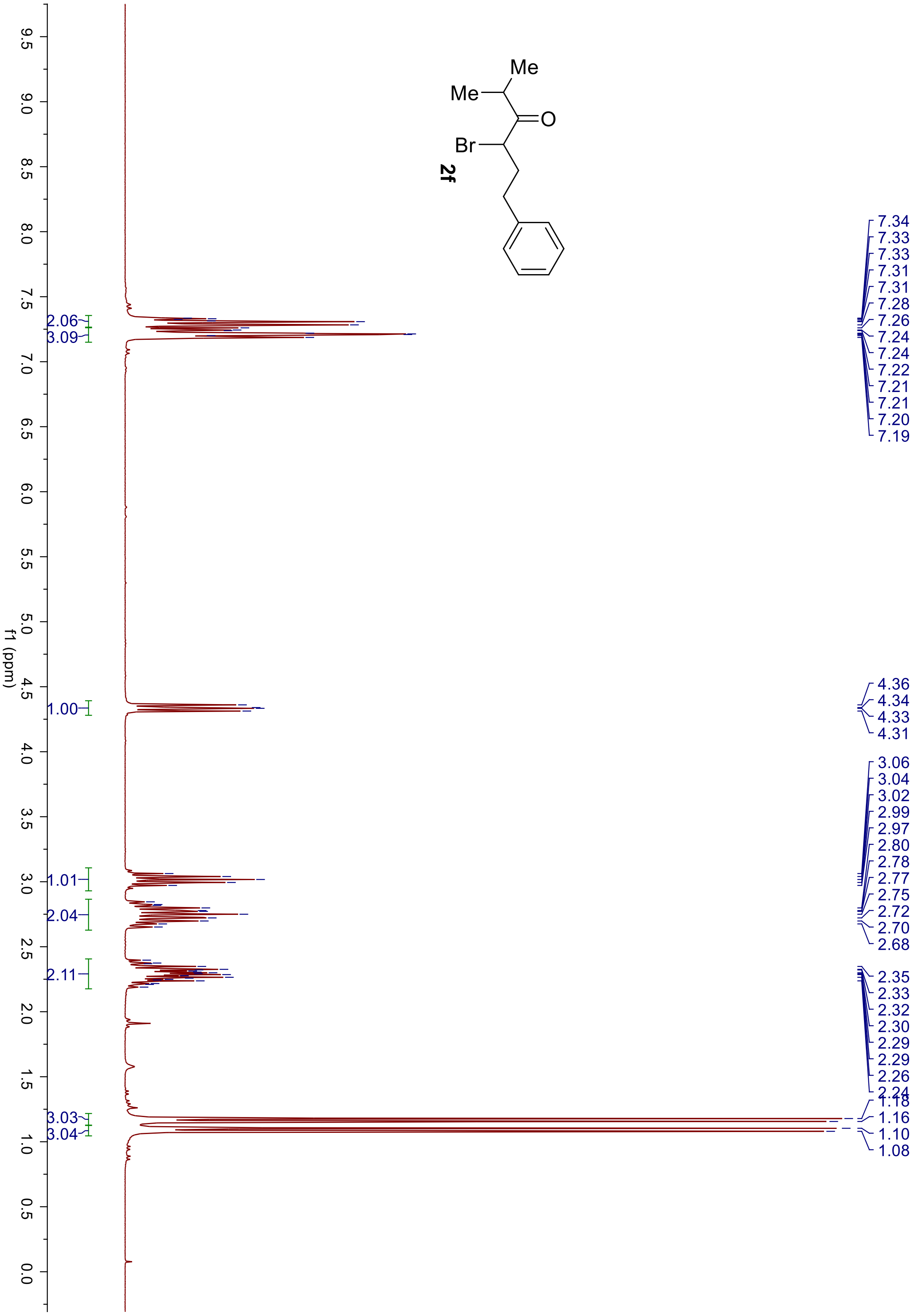

Me
Me
O
Br
2f
f1 (ppm)
9.5
9.0
8.5
8.0
7.5
7.0
6.5
6.0
5.5
5.0
4.5
4.0
3.5
3.0
2.5
2.0
1.5
1.0
0.5
0.0
2.06
3.09
1.00
1.01
2.04
2.11
3.03
3.04
7.34
7.33
7.33
7.31
7.31
7.28
7.26
7.24
7.24
7.22
7.21
7.21
7.20
7.19
4.36
4.34
4.33
4.31
3.06
3.04
3.02
2.99
2.97
2.80
2.78
2.77
2.75
2.72
2.70
2.68
2.35
2.33
2.32
2.30
2.29
2.29
2.26
2.24
1.18
1.16
1.10
1.08

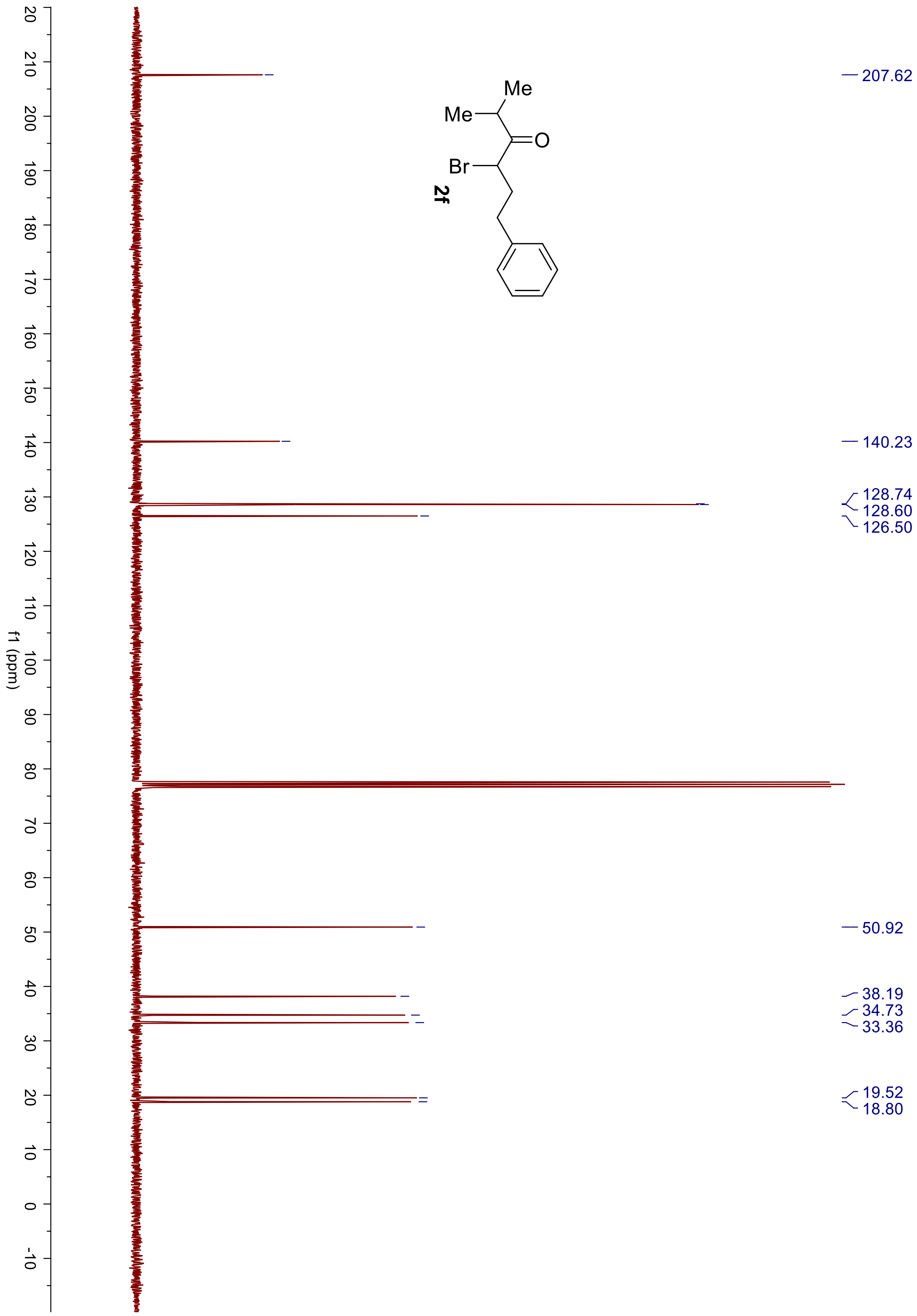
Me
Me
O
Br
2f
207.62
140.23
128.74
128.60
126.50
50.92
38.19
34.73
33.36
19.52
18.80
f1 (ppm)

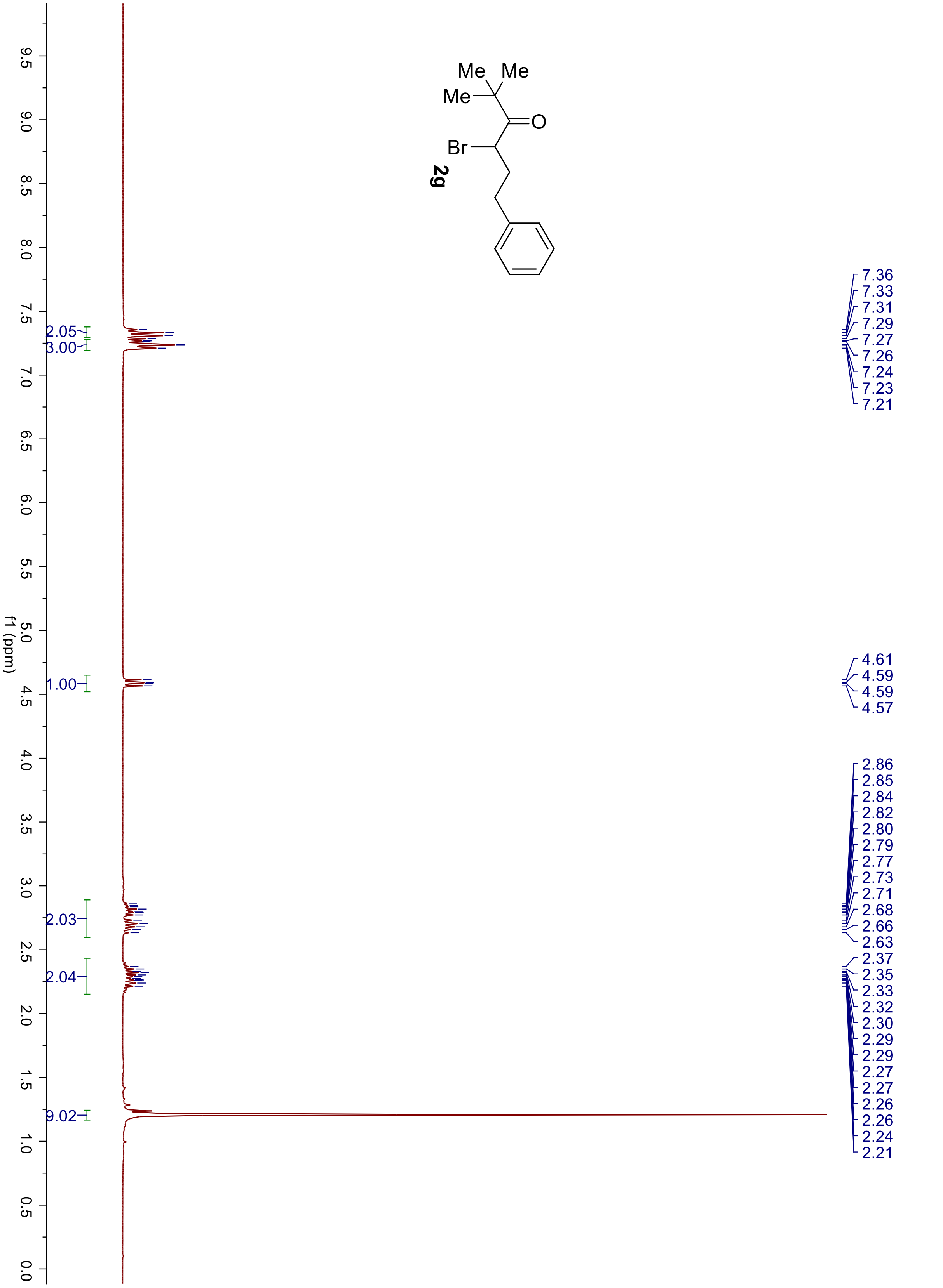
Me
Me
Me
O
Br
2g
f1 (ppm)
7.36
7.33
7.31
7.29
7.27
7.26
7.24
7.23
7.21
4.61
4.59
4.59
4.57
2.86
2.85
2.84
2.82
2.80
2.79
2.77
2.73
2.71
2.68
2.66
2.63
2.37
2.35
2.33
2.32
2.30
2.29
2.29
2.27
2.27
2.26
2.26
2.24
2.21
2.05
3.00
1.00
2.03
2.04
9.02

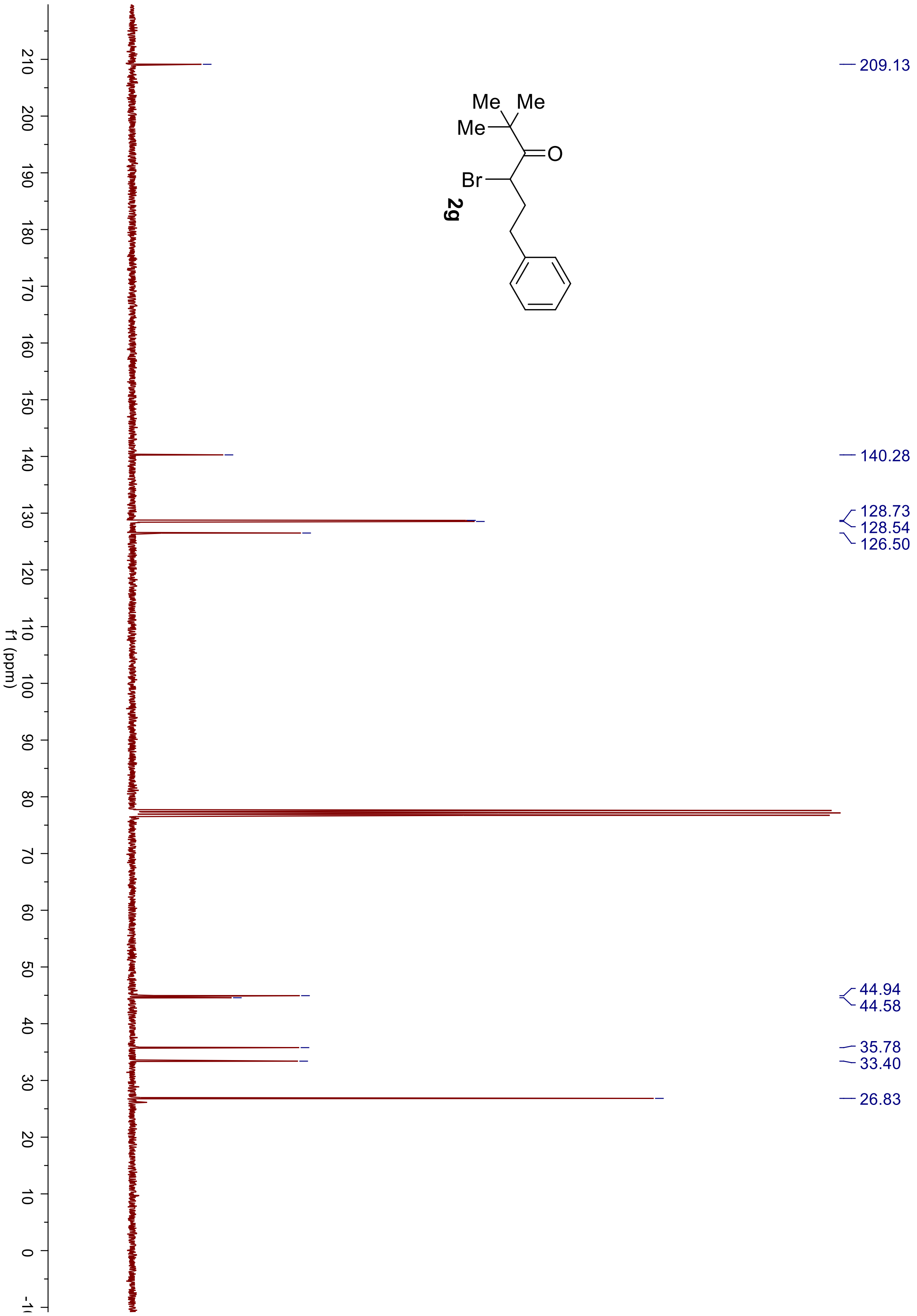
209.13
140.28
128.73
128.54
126.50
44.94
44.58
35.78
33.40
26.83
f1 (ppm)
2g
